# In silico analysis of phylogeny, structure, and function of arsenite oxidase from unculturable microbiome of arsenic contaminated soil

**DOI:** 10.1186/s43141-021-00146-x

**Published:** 2021-03-29

**Authors:** Siddhartha Pal, Kriti Sengupta

**Affiliations:** 1 National Centre for Cell Science, Ganeshkhind, Pune, 411007 India; 2 Bioenergy Group, Agharkar Research Institute, Gopal Ganesh Agarkar Road, Pune, 411004 India

**Keywords:** Arsenite oxidase, In silico, Homology modelling, *Rhizobiales*, Protein interactome

## Abstract

**Background:**

Arsenite oxidase (EC 1.20.2.1) is a metalloenzyme that catalyzes the oxidation of arsenite into lesser toxic arsenate. In this study, 78 amino acid sequences of arsenite oxidase from unculturable bacteria available in metagenomic data of arsenic-contaminated soil have been characterized by using standard bioinformatics tools to investigate its phylogenetic relationships, three-dimensional structure and functional parameters.

**Results:**

The phylogenetic relationship of all arsenite oxidase from unculturable microorganisms was revealed their closeness to bacterial order *Rhizobiales*. The higher aliphatic content showed that these enzymes are thermostable and could be used for in situ bioremediation. A representative protein from each phylogenetic cluster was analysed for secondary structure arrangements which indicated the presence of α-helices (~63%), β-sheets (57–60%) and turns (13–15%). The validated 3D models suggested that these proteins are hetero-dimeric with two chains whereas alpha chain is the main catalytic subunit which binds with arsenic oxides. Three representative protein models were deposited in Protein Model Database. The query enzymes were predicted with two conserved motifs, one is Rieske 3Fe-4S and the other is molybdopterin protein.

**Conclusions:**

Computational analysis of protein interactome revealed the protein partners might be involved in the whole process of arsenic detoxification by *Rhizobiales*. The overall report is unique to the best of our knowledge, and the importance of this study is to understand the theoretical aspects of the structure and functions of arsenite oxidase in unculturable bacteria residing in arsenic-contaminated sites.

**Supplementary Information:**

The online version contains supplementary material available at 10.1186/s43141-021-00146-x.

## Background

One of the greatest global threats to human health is arsenic contamination due to its high carcinogenic effect. Anthropogenic activities such as mining, agrochemical usage and industrial drainage into water bodies increase the risk of arsenic pollution in soil and water [1–3]. Arsenic polluted groundwater or soil refers to the existence of two soluble forms of arsenic commonly known as organo-arsenical anions which are arsenite As (III) and arsenate As (V). Certain soil microorganisms have the necessary enzyme system to detoxify the arsenic in presence of oxygen. Most commonly arsenite is oxidized by a microbial enzyme into arsenate which has lesser toxicity than the arsenite. Arsenite oxidase (EC 1.20.9.1) is the key enzyme that oxidizes arsenite, and it is located in the periplasmic membrane of several arsenic detoxifying bacteria [4]. Arsenite oxidase is a soluble metalloenzyme which requires molybdenum for its catalytic properties. It is a large heterodimer containing one large catalytic subunit which binds to arsenic and another small subunit-containing iron-sulfur Rieske protein [5]. It has been proposed that the electrons from arsenite oxidation are transferred to the molybdenum centre in a large subunit followed by a transfer to the 3Fe-4S Rieske cluster and finally to an electron acceptor such as cytochrome proteins [6].

Arsenite oxidase has been characterised in several uncultured bacteria in addition to few known bacterial genera such as *Aeromonas*, *Acinetobacter*, *Alcaligenes*, *Bosea*, *Pseudomonas*, and *Rhizobium*, mostly belonging to order *Rhizobiales.* These microorganisms generate energy deduced from the transition of the oxidation state of arsenic [7–9]. Recently several metagenomic studies of arsenic-contaminated soil suggested that there is a huge availability of arsenite oxidase in soil bacterium which are unculturable or unexplored to grow in laboratory conditions. The researchers have claimed a huge diversity and novelty in the gene sequence of this enzyme present in uncultured species of contaminated soil. Previously, few molecular enzyme assay methods were available to measure the arsenite oxidase activity in the cell-free filtrate by silver diethyl-dithiocarbamate method and Fiske-Subbarow method [7]. Membrane-bound arsenite oxidase from the bacterial cell was isolated by using polyacrylamide gel electrophoresis (SDS-PAGE) followed by identification through matrix-assisted laser desorption mass spectrometry (MALDI-TOF MS). Also, purified arsenite oxidase activity was checked by native PAGE [10]. The researchers are actively involved in searching for new molecular techniques to isolate such enzymes from unusual bacterial taxa [8, 11, 12]. Hence, arsenic-contaminated soil is the environment where arsenic detoxifying microorganisms are evolving under toxic environmental stress. The enzymology and genetic evidences of a bacterial process for arsenite oxidation facilitate the scientific approach regarding the arsenic bioremediation by using soil-derived bioactive compounds [13].

The arsenic oxidation capability of bacteria has been recently employed toward the removal of arsenic pollution in soil due to its effectiveness [9, 14]. However, enzyme technology has overpowered the use of bacterial cells due to its speedy and effective approach. The commercialization of important enzymes has encouraged the study of enzyme function and molecular structure to achieve a stable and improved enzymatic process. The application of pollutant degrading enzymes has been introduced as an eco-friendly alternative to several costly chemical treatment methods [15]. On the other hand, many soil microorganisms have not been characterized, because of its difficulty in cultivation under standard culture conditions. Thus, the soil eco flora is a prodigious reservoir for the hunting of novel microbial enzymes and bioactive molecules [16]. Isolation of novel enzymes from contaminated sites could be possible with combined efforts of computational analysis of biological parameters and high-throughput techniques along with laboratory experiments of analytical chemistry. Thus, various bioinformatics study of protein homology modelling to infer the functional structure have been coming into the scenario for biochemical characterization of such proteins with applications. Apart from isolating and characterizing numerous types of arsenite oxidase, extensive computational investigations of these enzymes have been successful to determine several unknown properties lying within the amino acid sequences which are often helpful prior to laboratory based studies. The consequences of these investigations are biotechnologically beneficial to employ them in environmental bioremediation perspectives. Hence, this study is focussing on computational analysis of phylogenetic, physicochemical properties, structural and functional analyses of the arsenite oxidase of unculturable bacteria and their closest relative bacteria to understand their unique properties essential for its applications in the field of arsenic bioremediation.

## Methods

### Sequence retrieval from databases

The amino acid sequences of arsenite oxidase of 60 uncultured bacteria already reported from arsenic-contaminated soil metagenome data available in databases were retrieved. The protein sequences of 18 classified strains reported for arsenic detoxification were also retrieved. Accession numbers of proteins and their respective cDNA sequences are provided in Supplementary table 1. All the peptide sequences and their respective cDNA sequences were retrieved in FASTA format from NCBI (National Center for Biotechnology Information) database (www.ncbi.nlm.nih.gov) for computational analysis.

### Phylogeny of arsenite oxidase

The phylogenetic tree of arsenite oxidase was constructed by using MEGA-X and Neighbor-joining model was used to calculate the distance between sequences by 500 bootstrapping method. The bootstrap values are indicated in the tree which was used to confirm how many times out of 500, the same branch was generated on repeating the phylogeny reconstructions. The higher bootstrap values refer to a high level of confidence of constructed clades in the tree. Phylogeny of the enzyme was deduced for both amino acid and their respective cDNA sequences. The sequences were aligned and trimmed for unmatched tail-end residues or base pairs prior to the construction of a phylogenetic tree by using in-built MEGA-X tools [17].

### Primary sequence analysis

The amino acid sequence analyses included determination of amino acid composition and physicochemical properties such as isoelectric point, molecular weight, instability index, aliphatic index, extinction coefficient, grand average of hydropathicity (GRAVY), positively charged and negatively charged residues. The physicochemical properties of arsenite oxidase were analysed by Expasy Protparam online tool (http://web.expasy.org/protparam).

### Secondary structure prediction

Protein folding prediction was performed by determining the number of α-helix, β-sheet and turns present in arsenite oxidase of a representative uncultured bacterium, and the same was compared with their closest known genera. Secondary structure prediction was achieved by PSIPRED (http://bioinf.cs.ucl.ac.uk/psipred) and CFSSP server (http://www.biogem.org/tool/chou-fasman) [18].

### Protein homology modelling and evaluation of protein 3D model

Arsenite oxidase of represented proteins was further selected as a query for computational analysis of protein structure. SWISS-Model workspace (https://swissmodel.expasy.org) was used to predict the 3D models of the enzyme by selecting the most suitable template [19]. Also, the predicted 3D structures were further visualized for its hydrophobic regions into the Swiss PDB Viewer (https://spdbv.vital-it.ch) [20]. The predicted protein model of arsenite oxidase was evaluated and verified from both QMEAN and SAVES v6.0 server (http:/nihserver.mbi.ucla.edu/SAVES). Ramachandran plot, VERIFY 3-D, ERRAT server and PROCHECK were assessed from SAVES v6.0 [21].

### Functional analysis: ligand binding and protein interactome

Ligand binding site was predicted by PrankWeb server (http://prankweb.cz) [22]. Cofactor of the enzyme was predicted by Cofactory 1.0 (https://services.healthtech.dtu.dk/service.php/Cofactory-1.0) [23]. Protein-protein interactome was predicted by STRING database version 11.0 (https://string-db.org) [24]. SignalP-5.0 server (http://www.cbs.dtu.dk/services/SignalP) was used to predict the signal peptide present in the protein which indicates the protein localization. TMHMM server 2.0 (http://www.cbs.dtu.dk/services/TMHMM) was used to predict the transmembrane helices in proteins. Additionally, a MOTIF search was performed to identify the protein family of this enzyme (https://www.genome.jp/tools/motif) [25].

## Results

### Phylogenetic analysis

**Fig. 1:**
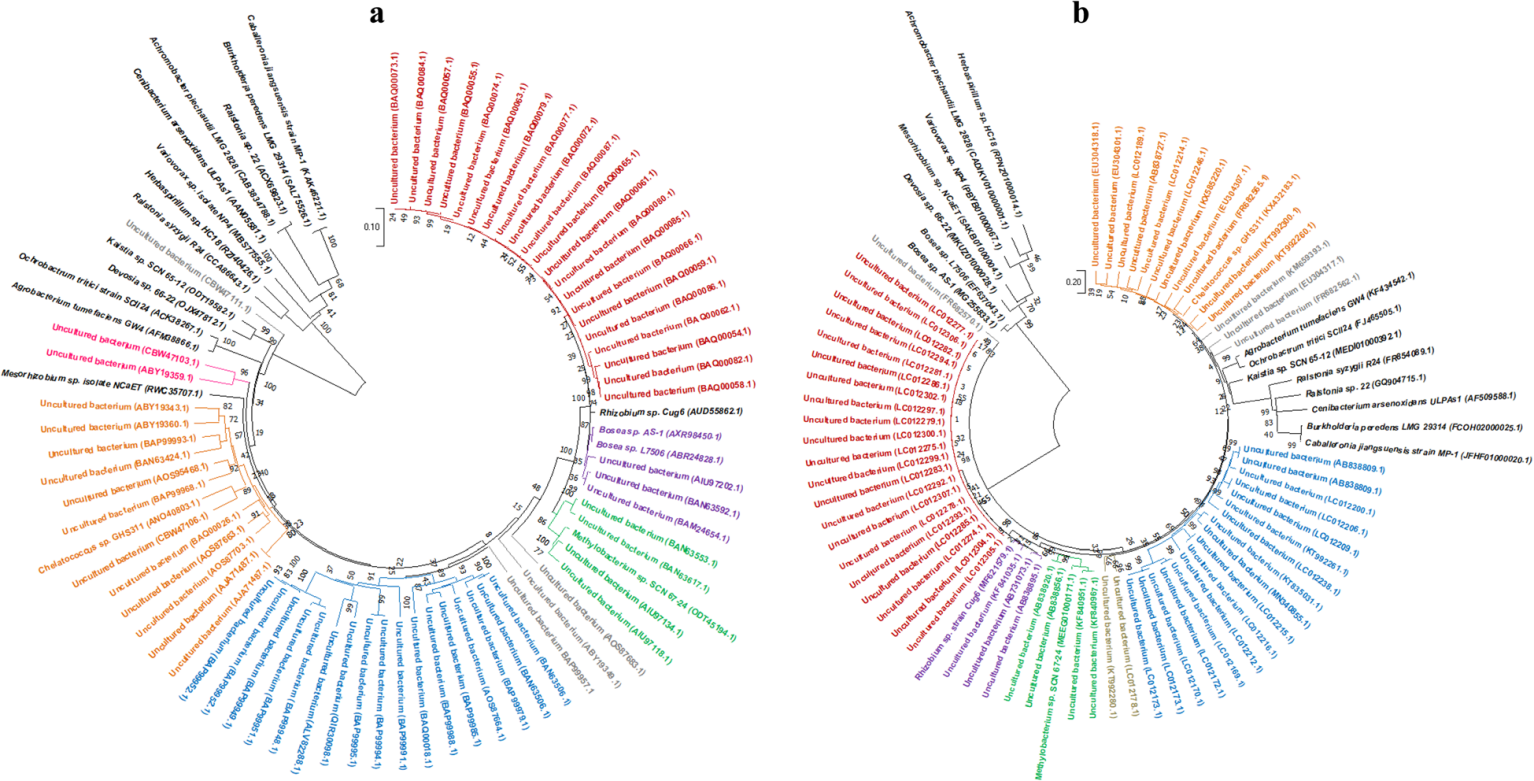
Amino acid-based phylogenetic analysis of 78 arsenite oxidase from uncultured bacteria and known bacterial genera (**a**) and gene sequence-based phylogenetic analysis of all selected arsenite oxidase from uncultured bacteria and known bacterial genera (**b**)

A total of 78 amino acid sequences of arsenite oxidase were retrieved from the NCBI proteins which included 60 proteins of uncultured bacteria reported in arsenic-contaminated soil metagenome and 18 proteins of several arsenic detoxifying bacteria. Phylogenetic relationship of arsenite oxidase was deduced among uncultured bacterium, and selected strains of known genera which are reported to detoxify arsenic such as *Achromobacter* sp. LMG 2828 (CAB3834788), *Agrobacterium* sp. GW4 (AFM38866), *Bosea* sp. strains AS-1, L7506 (AXR98450, ABR24828), *Burkholderia* sp. LMG 29314 (SAL75526), *Caballeronia* sp. MP-1 (KAK46221), *Cenibacterium* sp. ULPAs1 (AAN05581), *Chelatococcus* sp. GHS311 (ANO40803), *Devosia* sp. 66-22 (OJX47812), *Herbaspirillum* sp. HC18 (RZI40426), *Kaistia* sp. SCN 65-12 (ODT19582), *Mesorhizobium* sp. NCaET (RWC35707), *Methylobacterium* sp. SCN 67-24 (ODT45194), *Ralstonia* sp. strains 22, R24 (ACX69823, CCA86643), *Rhizobium* sp. Cug6 (AUD55862), *Ochrobactrum* sp. SCII24 (ACK38267) and *Variovorax* sp. NP4 (MBS77555). The selection of taxonomically known genera was based on BLASTp search for closely related genera and position-specific PSI-BLAST for distant relative genera which are involved in arsenic detoxification as per available reports [26–28]. The phylogeny based on amino acid sequences portrayed two large clusters of arsenite oxidase of uncultured bacteria shown in Fig. 1a (shown in red and blue color) which did not show match with the known classified genera. On the other hand, three clusters of uncultured bacterial proteins were identified which showed the closest neighbor as *Bosea*, *Chelatococcus* and *Methylobacterium* (clusters shown in purple, orange and green in Fig. 1b).

### Physicochemical parameters

**Fig. 2:**
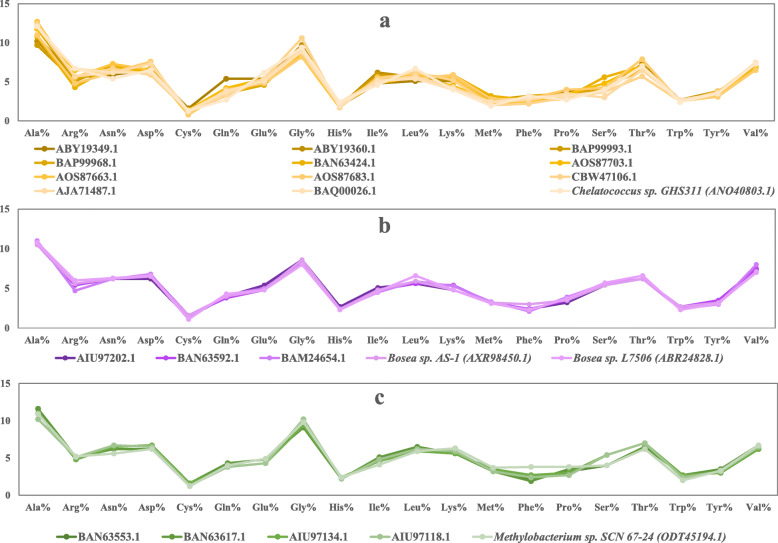
Comparative representation of differences in amnio acid composition of three phylogenetic cluster of uncultured bacteria with their closest neighbours *Chelatococcus* (**a**), *Bosea* (**b**) and *Methylobacterium* (**c**) (clusters shown in orange, purple and green in Fig. 2)

The theoretical physicochemical characters of arsenite oxidase were obtained from the linear amino acid sequence to predict the enzymatic functions. The parameters included composition and length of amino acid chain, molecular weight (kDa), isoelectric point (pI), extinction coefficient (EC), instability index (II), aliphatic index (AI) and grand average of hydropathicity (GRAVY). In this study, all the 78 arsenite oxidase sequences were characterized based on their physicochemical features by several computational tools (Table 1). The differences in the amino acid composition of arsenite oxidase present in different clusters of the related phylogenetic group are represented in Fig. 2 which are significantly (*P* < 0.05) similar in all strains of uncultured bacterium and classified strains of known culturable genera. The median length of the amino acid chain varied between 354 and 372 residues and molecular weight was in the range of 36 to 41 kDa in all uncultured bacterium. The enzyme arsenite oxidase is a large heterodimeric protein with two chains, one nearly 825 amino acid residues and other 133 residues [29], but the available sequences in the database were incomplete (maximum 400 residues) containing only alpha chain of arsenite oxidase designated as AroA which is the catalytic subunit. In this study, analyses are based on the catalytic subunit of alpha chain which possesses an arsenic binding conserved region. This study would be helpful to determine the properties of those enzymes whose source microorganisms are unculturable. The pI range varied widely between 5.5 and 8 among all the strains whereas mode pI was 6.5 among all uncultured bacterial enzymes. The analysis indicated that the isoelectric point is 5.5 to 8 for the enzyme which is in acidic to slight alkaline range. The pI are those values where amphoteric amino acid molecules show net zero charges and probably lose its ionic strength and consecutively affect the solubility of the protein in an aqueous environment. This information could be useful in the isolation of these enzymes from its environment by isoelectric focussing used in microbial metaproteome analysis [30]. The basic principle for isoelectric focusing lies in the pH dependence of the charges on the constituent amino acid side chains, non-proteinaceous adducts and prosthetic groups of proteins. By exposing proteins to electrophoresis in pH gradients, they become separated and focused on sharp and well-defined zones at pH values conforming to their individual pI. Since the metaproteome is the mixture of several proteins with a wide range of pI, knowledge of unique pI value for a specific protein is essential to be isolated by isoelectric focussing. The EC of the mentioned proteins were within a range of 60390 to 76110 M^-1^cm^-1^. The EC is proportional to absorption maxima which refers to the amount of light absorbed by the protein concentration at a certain wavelength. Here, the EC was calculated at 280 nm measured in water assuming all pair of Cys residues are reduced. The aliphatic index of all proteins was ranging from 69 to 75 which indicated their high thermostability. The GRAVY, calculated from ExPASy, showed in negative range (−0.394 to −0.499), which infers that the proteins have better interactions with water molecules. Also, the instability index for all proteins were lower than 40 and thus indicated that they were stable [31]**.**

**Table 1:** Amino acid sequence-based analysis of physicochemical features of all selected arsenite oxidase

	Bacterial source	Protein accession no.	AA	MW	PI	II	Al	EC	Gravy
1	Uncultured	BAQ00061.1	372	41.15	6.55	28.28	72.15	72880	−0.415
2	Uncultured	BAQ00063.1	372	41.12	6.55	27.87	72.15	73255	−0.413
3	Uncultured	BAQ00066.1	372	41.09	6.55	27.65	73.20	73255	−0.408
4	Uncultured	BAQ00080.1	372	41.09	6.55	26.87	73.20	73255	−0.403
5	Uncultured	BAQ00079.1	371	40.95	7.24	27.41	73.35	72880	−0.396
6	Uncultured	BAQ00085.1	372	41.08	6.83	26.03	73.00	72880	−0.424
7	Uncultured	BAQ00087.1	372	41.13	6.34	29.28	73.20	72880	−0.419
8	Uncultured	BAQ00072.1	372	41.10	6.55	28.39	73.98	72880	−0.410
9	Uncultured	BAQ00059.1	372	41.03	6.34	27.87	73.20	72880	−0.390
10	Uncultured	BAQ00065.1	372	41.08	6.50	28.51	73.47	74370	−0.398
11	Uncultured	BAQ00086.1	372	40.98	6.50	27.57	73.91	72880	−0.394
12	Uncultured	BAQ00077.1	372	41.13	6.83	26.54	74.25	73130	−0.422
13	Uncultured	BAQ00073.1	372	40.89	6.83	28.67	71.37	67755	−0.415
14	Uncultured	BAQ00057.1	372	40.99	6.55	29.42	71.10	67380	−0.430
15	Uncultured	BAQ00055.1	373	40.92	6.83	28.49	70.59	67380	−0.424
16	Uncultured	BAQ00084.1	372	41.03	7.76	28.22	71.69	67380	−0.410
17	Uncultured	BAQ00082.1	371	41.21	7.46	28.92	74.52	72880	−0.451
18	Uncultured	BAQ00062.1	372	40.93	6.45	28.15	72.10	78380	−0.443
19	Uncultured	BAQ00058.1	371	41.24	6.89	29.25	74.52	78380	−0.442
20	Uncultured	BAQ00054.1	372	41.03	7.46	29.18	72.88	78380	−0.461
21	Uncultured	BAQ00074.1	354	38.92	6.17	26.53	74.15	65890	−0.389
22	Uncultured	BAQ00026.1	371	40.91	6.44	24.00	75.00	74620	−0.370
23	Uncultured	BAQ00018.1	372	41.24	6.35	23.10	73.74	72880	−0.486
24	Uncultured	BAN63592.1	372	41.12	6.34	29.60	73.20	74370	−0.420
25	Uncultured	BAN63553.1	372	40.86	6.83	30.00	75.08	74370	−0.390
26	Uncultured	BAN63617.1	372	40.90	6.78	32.63	75.35	74370	−0.403
27	Uncultured	BAN63506.1	372	41.24	6.12	30.25	72.69	72880	−0.466
28	Uncultured	BAN63424.1	372	40.83	5.95	30.03	71.40	70360	−0.422
29	Uncultured	BAM24654.1	337	36.87	5.79	29.64	74.72	58900	−0.411
30	Uncultured	AIU97202.1	372	41.18	6.23	31.18	73.47	74370	−0.432
31	Uncultured	AIU97134.1	372	40.73	6.55	30.67	69.84	71390	−0.428
32	Uncultured	AIU97118.1	372	40.65	7.78	27.93	71.90	67380	−0.442
33	Uncultured	BAP99952.1	372	41.18	6.21	23.97	72.39	75860	−0.422
34	Uncultured	BAP99979.1	372	41.18	6.29	25.87	73.23	72880	−0.454
35	Uncultured	BAP99985.1	372	41.12	6.04	29.46	74.52	72880	−0.453
36	Uncultured	BAP99994.1	372	41.21	5.94	28.00	71.34	72880	−0.437
37	Uncultured	BAP99988.1	372	40.99	5.96	30.75	70.59	67380	−0.463
38	Uncultured	BAP99995.1	371	41.08	5.86	28.00	71.54	73130	−0.429
39	Uncultured	BAP99957.1	372	40.99	5.89	20.81	71.13	72880	−0.450
40	Uncultured	BAP99949.1	372	41.10	6.03	23.86	72.39	74620	−0.419
41	Uncultured	BAP99948.1	372	41.15	6.03	22.24	73.71	74370	−0.424
42	Uncultured	BAP99991.1	372	41.05	5.96	27.92	67.96	69120	−0.540
43	Uncultured	BAP99951.1	372	41.27	5.87	22.80	73.17	74370	−0.428
44	Uncultured	BAP99993.1	372	41.14	6.44	23.64	75.03	72880	−0.404
45	Uncultured	BAP99968.1	372	40.87	6.73	25.97	73.25	74370	−0.398
46	Uncultured	QIR30098.1	372	41.19	5.57	23.34	73.98	75860	−0.481
47	Uncultured	AJA71487.1	371	40.80	5.43	26.43	76.00	74370	−0.379
48	Uncultured	AOS95468.1	372	41.00	6.10	24.72	72.69	76110	−0.413
49	Uncultured	AOS87683.1	354	39.02	5.80	24.32	70.62	65890	−0.501
50	Uncultured	AOS87664.1	354	39.04	6.74	27.96	72.80	67380	−0.483
51	Uncultured	AOS87703.1	354	39.01	6.44	30.59	72.00	65890	−0.473
52	Uncultured	AOS87663.1	354	38.97	6.37	24.96	76.72	67380	−0.437
53	Uncultured	ABY19349.1	372	41.00	5.87	25.22	70.59	74370	−0.440
54	Uncultured	ABY19360.1	372	41.04	6.03	30.48	75.83	75860	−0.397
55	Uncultured	ABY19343.1	372	40.87	6.49	24.51	71.67	74370	−0.427
56	Uncultured	ABY19359.1	371	41.06	5.26	27.16	69.73	72880	−0.518
57	Uncultured	CBW47111.1	404	44.19	5.73	22.04	73.44	74370	−0.405
58	Uncultured	CBW47103.1	403	44.41	6.83	28.34	70.40	75860	−0.499
59	Uncultured	CBW47106.1	404	43.91	6.44	21.04	79.28	77350	−0.344
60	Uncultured	ALV82288.1	367	40.76	5.96	29.19	74.17	68870	−0.500
61	*Bosea sp.* AS-1	AXR98450.1	372	41.14	6.34	30.02	72.69	72880	−0.406
62	*Bosea sp.* L7506	ABR24828.1	351	38.69	6.49	33.66	74.81	60390	−0.443
63	*Rhizobium sp.* Cug6	AUD55862.1	377	41.62	7.24	27.51	71.72	74370	−0.436
64	*Chelatococcus sp.* GHS311	ANO40803.1	372	40.90	5.78	24.91	77.18	68870	−0.347
65	*Achromobacter piechaudii* LMG 2828	CAB3834788.1	827	92.31	7.25	30.12	70.23	145650	−0.499
66	*Burkholderia peredens* LMG 29314	SAL75526.1	825	91.79	6.80	27.78	67.31	137545	−0.502
67	*Caballeronia jiangsuensis* MP-1	KAK46221.1	825	91.82	6.98	27.77	67.78	137170	−0.505
68	*Ralstonia sp.* 22	ACX69823.1	827	92.33	6.81	30.79	69.88	145650	−0.508
69	*Cenibacterium arsenoxidans* ULPAs1	AAN05581.1	826	91.64	8.43	36.06	68.44	146680	−0.479
70	*Agrobacterium tumefaciens* GW4	AFM38866.1	845	93.26	6.40	30.85	71.73	125710	−0.469
71	*Ochrobactrum tritici* SCII24	ACK38267.1	846	93.61	6.09	35.16	70.96	124220	−0.468
72	*Ralstonia syzygii* R24	CCA86643.1	824	92.27	7.96	31.54	73.19	130180	−0.459
73	*Variovorax sp.* NP4	MBS77555.1	827	91.18	8.66	23.34	68.60	144160	−0.451
74	*Herbaspirillum sp.* HC18	RZI40426.1	827	92.14	8.42	34.82	70.46	134650	−0.469
75	*Methylobacterium sp.* SCN 67-24	ODT45194.1	820	90.23	8.29	30.19	69.30	128230	−0.431
76	*Mesorhizobium sp.* NCaET	RWC35707.1	869	95.66	5.90	34.85	70.31	132700	−0.499
77	*Devosia sp.* 66-22	OJX47812.1	821	90.27	5.32	31.16	72.80	141180	−0.400
78	*Kaistia sp.* SCN 65-12	ODT19582.1	821	90.23	5.33	27.75	72.20	141180	−0.419

### Secondary structure analysis

The phylogeny and amino acid composition variation helped in clustering a large amount of closely similar sequences, and only three proteins of each three clusters of uncultured bacteria were selected based on their lower instability index. Here, these putative arsenite oxidase enzymes of representative strains were named as ‘uncultured-1 (AOS87703), uncultured-2 (BAM24654) and uncultured-3 (AIU97134)’ which showed sequence similarity with *Bosea*, *Chelatococcus* and *Methylobacterium*, respectively. Secondary structure arrangements in these proteins were consisting of mainly three types of secondary components, α-helices, β-sheets and turns (Supplementary data 3). The helical content (Table 2) was maximum in *Methylobacterium* sp. SCN 67-24 (66.5%), followed by query proteins 1 (62.7%), 2 (62.9%), 3 (62.9%), *Bosea* sp. AS-1 (62.4%) and *Chelatococcus* sp. GHS311 (59.1%). There are 10 α-helix regions were predicted by PSIPRED in three query proteins, i.e., in secondary structure of query 1 (AOS87703), residue positions 70-80 (VRGARMAEMSY), 109-125 (WDDALDLVARVTCAVINDQ'), 150-157 (GKLYFEAM), 173-180 (SEVHATRD), 128-137 (SEVHATRD), 254-264 (TVNACEVEAGK), 276-290 (TDLALFNAWFTHINE), 306-313 (LDKALAAN), 318-324 (LDEAARIT) and 327-339 (VDQIRQSAEWIA). The similar α regions were also predicted in other two query proteins shown in supplementary data 3. The median percentage of β-sheet was 59% which also significantly indicated the thermodynamic stability of the protein structure. The β-sheets are the most prevalent secondary element involved in the functional structure of metalloproteins [32].

**Table 2:** Comparison of secondary and tertiary protein structural properties among selected uncultured bacterium and their closest bacterial genera

Sl. No.	Name and affiliation of bacterium	Protein accession no.	Secondary structure organization			Tertiary structure quality	
			α-helix (%)	β-sheet (%)	Turns (%)	QMEAN score	Amino acid in favoured region in Ramachandran plot (%) ^#^
1	Uncultured-1	AOS87703.1	62.7	57.3	13.8	-1.55	95.58
2	Uncultured-2	BAM24654.1	62.9	59.9	14.8	-0.68	96.25
3	Uncultured-3	AIU97134.1	62.9	58.6	15.1	-1.14	95.27
4	*Chelatococcus* sp. GHS311	ANO40803.1	59.1	59.7	13.4	-1.43	95.14
5	*Bosea* sp. AS-1	AXR98450.1	62.4	61.0	14.5	-0.84	95.14
6	*Methylobacterium* sp. SCN 67-24	ODT45194.1	66.5	38.3	14.8	-1.04	93.80

Acceptable QMEAN score; ^#^a good quality model would be expected to have over 90% in the most favoured regions

### Protein homology modelling and evaluation

**Fig. 3:**
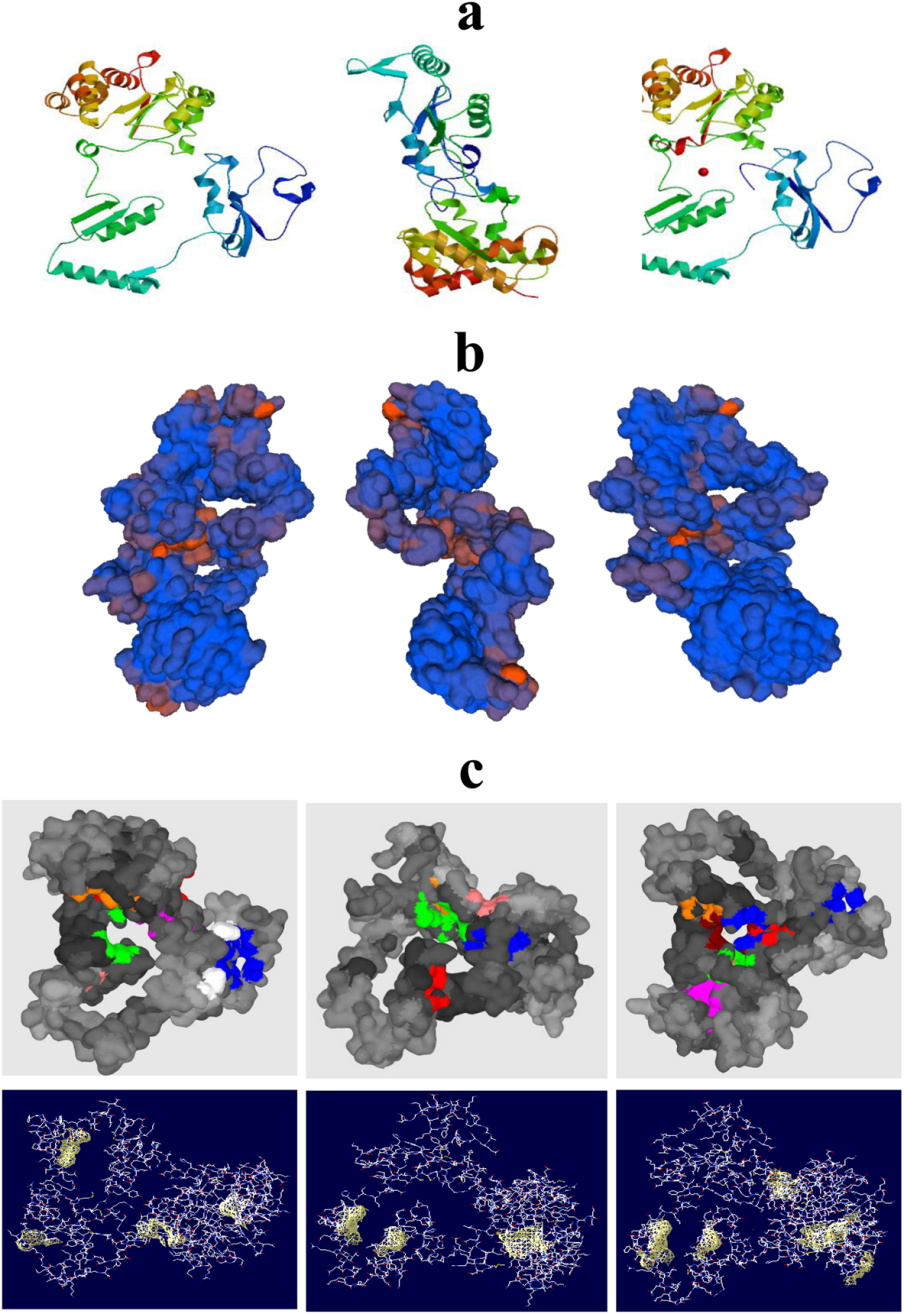
Three-dimensional models of monomeric chain A of arsenite oxidase of three query proteins (**a** query proteins 1, 2 and 3 are shown in the left, middle and right, respectively), surface views of heterodimeric predicted 3D models of three representative arsenite oxidase (**b** query proteins 1, 2 and 3 are shown in the left, middle and right, respectively), PrankWeb prediction of ligand binding pockets (upper) and yellow patches are hydrophobic regions (lower) present in heterodimeric structure of three representative arsenite oxidase (**c** query proteins 1, 2 and 3 are shown in the left, middle and right, respectively)

Homology modelling was performed for all three representative proteins of uncultured bacteria and their respective closest known genera by selecting their most suitably matched template 3D protein model suggested by SWISS MODEL. The predicted 3D structures were evaluated on the basis of permissible QMEAN score, an overall quality parameter from SAVES v6.0 server and maximum amino acid percentage in favoured region of Ramachandran plot (Table 2, supplementary data 6). Arsenite oxidase (PDB ID 5NQD) was selected as the most suitable template on the basis of its high sequence similarity (53–54%) with the query proteins. The homology modelling was performed to draw a hypothesis of the function of protein sequence available in unclassified metagenome sequences of the arsenic-contaminated environment. The QMEAN score of predicted models 1, 2 and 3 were −1.55, −0.68 and −1.14, respectively, which are statistically acceptable for valid 3D structures [19]. Visualization of alignments and QMEAN score (shown in blue bars) of models 1, 2 and 3 with template 5NQD is provided in supplementary data 7. These alignments suggested that the 3D structure of query protein 1 has been predicted from amino acid residue position 61 to 332 (length 272 residues) while residues from 35 to 395 (361 residues) and 60 to 383 (323 residues) in queries 2 and 3, respectively. The predicted 3D model suggested that the protein is composed of an alpha chain which has been corroborated with previous report that the enzyme arsenite oxidase is hetero-dimeric protein with two chains where the alpha chain is the main catalytic subunit which actively binds with arsenic oxides [29]. The predicted 3D model of the monomeric alpha chain of three representative proteins of uncultured bacteria is shown in Fig. 3a whereas surface views of constructed dimeric proteins depicted the overall cavities and grooves present in the functional structure for several molecular associations (Fig. 3b). PrankWeb prediction of ligand binding sites revealed the presence of several ligand-binding pockets present in the representative proteins and a model visualizing tool swiss PDB viewer constructed the hydrophobic regions of the enzyme shown in yellow patches in Fig. 3c. The cofactory 1.0 server suggested that the proteins have FAD/NADP binding specificity above score 0.5 (Supplementary data 3). Assessment and quality check of the built 3D (.pdb) model was executed, and Ramachandran plot was constructed to show the locations assigned for each amino acid residues in favoured regions (Table 2). An acceptable QMEAN score (−0.68 to −1.55) and the presence of more than 95–96% amino acid residues in Ramachandran plot was determined in all representative proteins. The existence of more than 90% amino acids in the favoured region of Ramachandran plot of a protein could be considered as the good quality protein model [33]. The *Z*-score of the query protein sequence was within an acceptable range, i.e. 1<[*Z*-score] <2, in comparison with PDB non-redundant protein matches. The protein model evaluation by VERIFY 3D and PROCHECK suggested that there are very less unfavourable conformations, and it was predicted with better resolution (1.5 to 2 Å) along with best-fit planarity (Supplementary data 4, 5 and 8). A similar type of model validation was also conducted for other families of enzymes [18]. The predicted models were also qualified for the quality assessment by the ERRAT server where a good resolution structure generally produces a quality factor value around 95% or higher. In this assessment, models 1, 2 and 3 produced a quality factor value of 99.11, 98.72 and 99.71, respectively, which reconfirmed their structural high resolution. The three representative protein models were deposited in Protein Model Database (in .pdb format), and its accession numbers obtained were PM0083212, PM0083213 and PM0083214. The mentioned models are now available in a public database.

### Function prediction

**Fig. 4:**
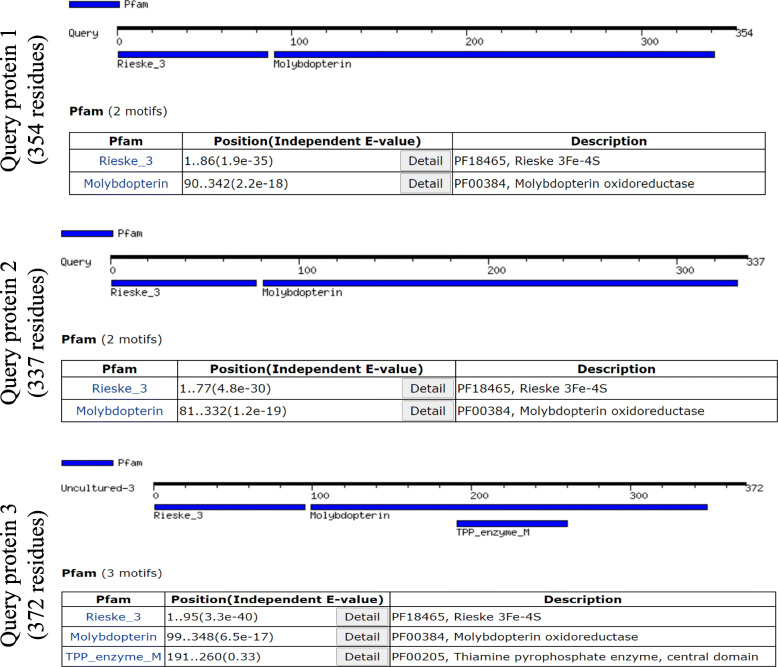
Motifs predicted in three query protein sequences of arsenite oxidase from uncultured bacteria

**Fig. 5:**
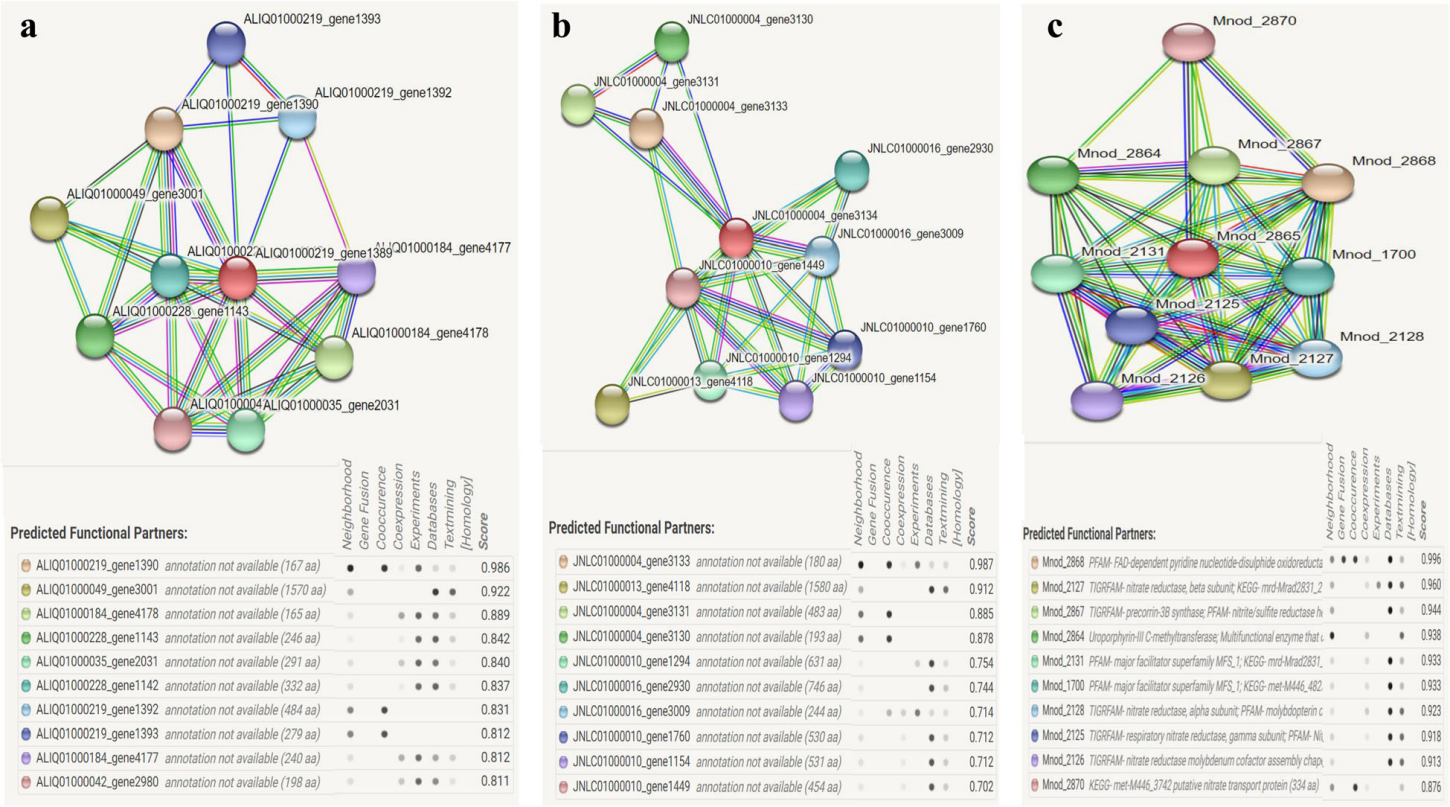
STRING analysis of protein interacting network of arsenite oxidase from *Chelatococcus* sp. (**a**), *Bosea* sp. (**b**), and *Methylobacterium* sp. (**c**)

The function of the query enzyme was determined primarily by identifying the conserved motif search which suggested that the query proteins possess two conserved motifs, one is Rieske 3Fe-4S (Pfam ID: PF18465) and the other is molybdopterin (PF00384) shown in Fig. 4. Previous reports suggested that Rieske 3Fe-4S domain is responsible for maintaining redox potential for arsenic detoxification, and the other motif is for molybdenum binding as a cofactor for the functional enzyme [6, 29]. Protein localization was predicted by detecting the presence of a signal peptide which confirms that these proteins are membrane-bound (Supplementary data 9). Interactive pockets also have identified in the 3D models (Fig. 3c), and models 1 and 3 contain an interactive groove within the first 50 amino acid residues and having a lesser evolutionary conservation score of 0.55 which indicated its promiscuity toward several molecular interactions [22]. STRING server predicted that the query protein directly interacts with several proteins in known genera such as *Bosea*, *Chelatococcus* and *Methylobacterium* which were the closest neighbours of the representative proteins. The protein interactome of arsenite oxidase included arsenite reductase, glutamate synthase, nitrate reductase, cytochrome c, 4Fe-4S di-cluster containing protein, histidine kinase, cytochrome c3 and formate dehydrogenase (Fig. 5). The presented protein-protein network included cytochrome (c3) that indicated towards its function of arsenite oxidation process where oxidation of arsenite is coupled with ATP formation by c3 [6].

## Discussion

**Fig. 6:**
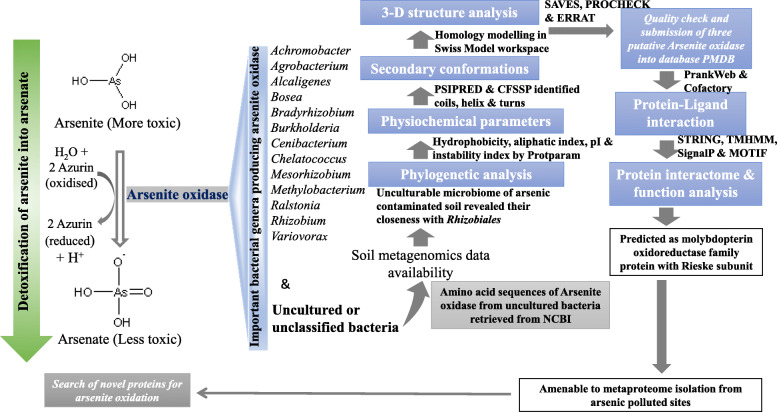
Flow diagram to represent the in silico study of arsenite oxidase of unculturable bacteria of arsenic-contaminated sites

In this study, the phylogenetic clustering helped in deducing the sequence similarity of the unknown bacterium with known *Rhizobiales* order which generally includes rhizospheric soil habitant bacteria. Thus, three clusters that indicated amino acid sequence homology with three taxonomically classified genera were selected for further analysis. Essentially, the primary linear chain of an amino acid sequence is a translated message from its coding gene sequence, but DNA sequences are much likely to recombination or mutations especially in prokaryotes by transposons or horizontal gene transfer within relative species [34]. The gene diversity is more frequent in comparison to diversity in protein sequences. Therefore, the cDNA sequence of each protein was also implicated for phylogenetic analysis to presume the diversity based on the coding DNA sequence of the arsenite oxidase. In this case, the gene-based phylogeny of the enzyme was similar to amino acid-based phylogeny where the largest clade included the various uncultured strains which did not show sequence similarity to any classified bacterial strains used in this study. The equivalent phylogenetic analysis was also performed by some earlier researchers to interpret the evolutionary significance of arsenite oxidase of different taxa based on their gene and primary protein sequences [35–37]. However, the availability of huge metagenomic data in recent years has been understudied for enzyme phylogeny, structure and function analysis of putative arsenite oxidase present in an uncultured microbial community. The computational analysis of such enzymes would be amenable to meta-proteomics study towards expedition of novel enzymes of environmental origin. Further amino acid sequence-based physiochemical parameters directed toward the isolation and purification of such enzymes. The theoretical information regarding arsenite oxidase enzymes such as its net charge, stability, hydrophobicity and thermostability could be useful in further application-based studies. Importantly, the higher aliphatic residue content indicated that these enzymes are thermostable and could be used for in situ bioremediation application. It is globally believed that the poor thermostability of enzymes has limited application as biocatalysts in process development, and thus, researchers are actively involved in hunting for highly thermostable enzymes for application purpose [14]. Additionally, predicted functional activities of these enzymes based on their secondary, tertiary structure and co-factor binding sites corroborated with previous studied [5, 6, 10]. In present study, query enzymes were heterodimeric in nature with molybdopterin which is so far commonly found in all aerobic arsenite oxidase. The presence of ligand molybdopterin in predicted structure like other available structure of arsenite oxidase is mainly involved in the enzymatic mechanism which related to interaction with coordinated metal. The RCSB PDB database survey of arsenite oxidase suggested that major ligand binding sites contribute to the enzymatic detoxification of arsenic in *Rhizobium* sp. are 3**Fe-4S cluster** (FCXHZBQOKRZXKS-MZMDZPPWAW), **molybdenum (IV) ion** (ZIKKVZAYJJZBGE-UHFFFAOYSA-N) and **oxygen atom** (XLYOFNOQVPJJNP-UHFFFAOYSA-N). These domains are not commonly conserved within the bacterial genus. Generally, functional site of arsenite oxidase lies at the Mo-site where arsenite is oxidized to arsenate and reducing the Mo (oxidation state from +VI to +IV). Since 3Fe-4S cluster is a one electron acceptor site, it is expected that it receives one electron from the molybdopterin and then transfers single electron to the Rieske 2Fe-2S cluster of the B subunit. In aerobic arsenotrophic microorganism, the electron is consecutively transferred from the Rieske centre to cytochrome *c* (physiological electron acceptor), and lastly, oxygen is reduced to water. However, the function of the molybdopterin is not yet fully understood [10]. Most significantly, these three query proteins have been predicted as extracellular and supposed to be active when exposed to extracellular pH or salinity. The predicted protein structures in this study are classified as molybdopterin oxidoreductase family protein with Rieske subunit. However, there is no evidence of the presence of molybdopterin guanine dinucleotide cofactors and Mo-interactive conserved domain Cys21-X_2_-Cys24-X_3_-Cys28-X_70_-Ser99 in our predicted models unlike other available structures of arsenite oxidase belonging to betaproteobacteria [6]. Although the present study includes some theoretical analysis of protein structure and function, it carries significance in terms of strengthening the future experimental design of protein-protein interaction and check the feasibility of enzymatic reactions. This study might be helpful to design experiments in a sorted way as the important information regarding thermostability index, instability index, isoelectric point, extinction coefficient and alpha-helical content might be helpful to separate and purify the enzyme from its sources. A summarization of application of bioinformatics tools is represented schematically in Fig. 6 to visualize this present study of arsenite oxidase belonging to unculturable bacteria of arsenic contaminated sites.

## Conclusion

In silico characterization of arsenite oxidase enzyme of uncultured bacteria from arsenic-contaminated soil revealed that the enzyme is a heterodimer with two subunits, and it is a molybdopterin protein. The phylogeny-based clustering of selected proteins of uncultured bacteria with known genera suggested that most of these proteins were close to *Bosea*, *Chelatococcus* and *Methylobacterium*, genera belonging to order *Rhizobiales*. This clustering provided the phylogenetic relationship between putative arsenite oxidase of unculturable bacteria and known bacterial groups found in arsenic-contaminated sites. The physicochemical properties suggested that they are thermostable and might be potential for biotechnological applications and detoxification of arsenic. Signal peptide prediction and transmembrane helix suggested that they are localized in the cellular membrane. Ligand binding pockets and hydrophobic regions in these proteins make them soluble and thus capable of secreting out in periplasmic space for oxidation-reduction reactions. Computational analysis of protein-protein interaction proposed that its protein partners might be involved in the whole process of arsenic detoxification. One of the interacting protein partners was predicted as cytochrome which indicated that these uncultured bacteria might be oxidizing arsenite to arsenate for ATP formation. Hence, this useful summation of bioinformatical information regarding the arsenite oxidase enzyme from unculturable bacteria residing in arsenic-contaminated soil could be helpful in searching for novel bioactive enzymes.

## Supplementary Information

**Additional file 1.** List of accession numbers of proteins and cDNA sequences for all selected 78 arsenite oxidase that was retrieved from NCBI for computational analysis in present study.

**Additional file 2.** Secondary structure analysis of representative proteins.

**Additional file 3.** Cofactors predicted by Cofactory 1.0 for query proteins.

**Additional file 4.** Homology modelling of representative enzyme obtained from SWISS MODEL.

**Additional file 5.** Evaluation reports predicted protein models of representative enzyme obtained from SAVES server.

**Additional file 6.** Graphical representation for amino acid distribution in Ramachandran plot of three predicted model.

**Additional file 7.** Visualization of alignments and QMEAN score (shown in blue bars) of model 1, 2, and 3 with template 5NQD.

**Additional file 8.** PROCHECK model evaluation report for query proteins.

**Additional file 9.** Signal peptide prediction using SignalP 5.0 server in query proteins and their closest phylogenetic members.

## Data Availability

Additional data is provided as supplementary material.

## Abbreviations

AI: Aliphatic index
Aro: Arsenite oxidase
cDNA: Complementary deoxyribonucleic acid
EC: Extinction coefficient
GRAVY: Grand average of hydropathicity
II: Instability index
MEGA: Molecular evolutionary genetic analysis
NCBI: National center for biotechnology information
PDB: Protein data bank
PMD: Protein model database
