# Supplementary material for: In silico analysis of phylogeny, structure, and function of arsenite oxidase from unculturable microbiome of arsenic contaminated soil
Source: J Genet Eng Biotechnol. 2021 Mar 29;19:47. doi: 10.1186/s43141-021-00146-x (PMC8006529; doi:10.1186/s43141-021-00146-x)
Supplement: Supplementary file 1 — Additional file 1. List of accession numbers of proteins and cDNA sequences for all selected 78 arsenite oxidase that was retrieved from NCBI for computational analysis in present study. [file 43141_2021_146_MOESM1_ESM.docx]

**Supplementary table 1:** Accession numbers of all arsenite oxidase selected in this study.

| Sl. No. | Arsenite oxidase A chain | NCBI accession numbers | |
| --- | --- | --- | --- |
|  |  | Protein | cDNA |
| 1 | Uncultured bacterium | BAQ00061.1 | LC012281.1 |
| 2 | [from metagenome of arsenite contaminated soil] | BAQ00063.1 | LC012283.1 |
| 3 |  | BAQ00066.1 | LC012286.1 |
| 4 |  | BAQ00080.1 | LC012300.1 |
| 5 |  | BAQ00079.1 | LC012299.1 |
| 6 |  | BAQ00085.1 | [LC012305.1](https://www.ncbi.nlm.nih.gov/nuccore/LC012305.1) |
| 7 |  | BAQ00087.1 | LC012307.1 |
| 8 |  | BAQ00072.1 | LC012292.1 |
| 9 |  | BAQ00059.1 | LC012279.1 |
| 10 |  | BAQ00065.1 | LC012285.1 |
| 11 |  | BAQ00086.1 | LC012306.1 |
| 12 |  | BAQ00077.1 | LC012297.1 |
| 13 |  | BAQ00073.1 | LC012293.1 |
| 14 |  | BAQ00057.1 | LC012277.1 |
| 15 |  | BAQ00055.1 | LC012275.1 |
| 16 |  | BAQ00084.1 | LC012304.1 |
| 17 |  | BAQ00082.1 | LC012302.1 |
| 18 |  | BAQ00062.1 | LC012282.1 |
| 19 |  | BAQ00058.1 | LC012278.1 |
| 20 |  | BAQ00054.1 | LC012274.1 |
| 21 |  | BAQ00074.1 | LC012294.1 |
| 22 |  | BAQ00026.1 | LC012246.1 |
| 23 |  | BAQ00018.1 | LC012238.1 |
| 24 |  | BAN63592.1 | AB838895.1 |
| 25 | Uncultured bacterium | BAN63553.1 | AB838856.1 |
| 26 | [from lake sediment] | BAN63617.1 | AB838920.1 |
| 27 |  | BAN63506.1 | AB838809.1 |
| 28 |  | BAN63424.1 | AB838727.1 |
| 29 |  | BAM24654.1 | AB731073.1 |
| 30 | Uncultured bacterium | AIU97202.1 | KF841035.1 |
| 31 | [from aquifers] | AIU97134.1 | KF840967.1 |
| 32 |  | AIU97118.1 | KF840951.1 |
| 33 |  | BAP99952.1 | LC012173.1 |
| 34 | Uncultured bacterium | BAP99979.1 | LC012200.1 |
| 35 | [from metagenome of arsenite contaminated soil] | BAP99985.1 | LC012206.1 |
| 36 |  | BAP99994.1 | LC012215.1 |
| 37 |  | BAP99988.1 | LC012209.1 |
| 38 |  | BAP99995.1 | LC012216.1 |
| 39 |  | BAP99957.1 | LC012178.1 |
| 40 |  | BAP99949.1 | LC012170.1 |
| 41 |  | BAP99948.1 | LC012169.1 |
| 42 |  | BAP99991.1 | LC012212.1 |
| 43 |  | BAP99951.1 | LC012172.1 |
| 44 |  | BAP99993.1 | LC012214.1 |
| 45 |  | BAP99968.1 | LC012189.1 |
| 46 | Uncultured bacterium (unpublished) | QIR30098.1 | MN340855.1 |
| 47 | Unculturable, As contaminated paddy soil | AJA71487.1 | KM659393.1 |
| 48 |  | AOS95468.1 | KX585220.1 |
| 49 | Uncultured bacterium | AOS87683.1 | KT992280.1 |
| 50 | [hot spring sediment] | AOS87664.1 | KT992261.1 |
| 51 |  | AOS87703.1 | KT992300.1 |
| 52 |  | AOS87663.1 | KT992260.1 |
| 53 | Uncultured bacterium [from As contaminated soil] | ABY19349.1 | EU304307.1 |
| 54 |  | ABY19360.1 | EU304318.1 |
| 55 |  | ABY19343.1 | EU304301.1 |
| 56 |  | ABY19359.1 | EU304317.1 |
| 57 |  | CBW47111.1 | FR682570.1 |
| 58 | Gold and arsenic mines | CBW47103.1 | FR682562.1 |
| 59 | Tomczyk-Żak et al., 2013 | CBW47106.1 | FR682565.1 |
| 60 | As cont soil (Sanyal et al., 2016) | ALV82288.1 | KT835031.1 |
| 61 | *Bosea* sp. AS-1 | AXR98450.1 | MG255833.1 |
| 62 | *Bosea* sp. L7506 | ABR24828.1 | EF637043.1 |
| 63 | *Rhizobium* sp. strain Cug6 | AUD55862.1 | MF621579.1 |
| 64 | *Chelatococcus* sp. GHS311 | ANO40803.1 | KX432183.1 |
| 65 | *Achromobacter piechaudii* LMG 2828 | CAB3834788.1 | CADIKV010000001.1 |
| 66 | *Burkholderia peredens* LMG 29314 | SAL75526.1 | FCOH02000025.1 |
| 67 | *Caballeronia jiangsuensis* strain MP-1 | KAK46221.1 | JFHF01000020.1 |
| 68 | *Ralstonia* sp. 22 | ACX69823.1 | GQ904715.1 |
| 69 | *Cenibacterium arsenoxidans* ULPAs1 | AAN05581.1 | AF509588.1 |
| 70 | *Agrobacterium tumefaciens* strain GW4 | AFM38866.1 | KF434542.1 |
| 71 | *Ochrobactrum tritici* strain SCII24 | ACK38267.1 | FJ465505.1 |
| 72 | *Ralstonia syzygii* R24 | CCA86643.1 | FR854089.1 |
| 73 | *Variovorax* sp. isolate NP4 | MBS77555.1 | PBYB01000067.1 |
| 74 | *Herbaspirillum* sp. HC18 | RZI40426.1 | RPNZ01000014.1 |
| 75 | *Methylobacterium* sp. SCN 67-24 | ODT45194.1 | MEEG01000171.1 |
| 76 | *Mesorhizobium* sp. isolate NCaET | RWC35707.1 | SAKB01000004.1 |
| 77 | *Devosia* sp. 66-22 | OJX47812.1 | MKUZ01000028.1 |
| 78 | *Kaistia* sp. SCN 65-12 | ODT19582.1 | MEDI01000392.1 |
