# Supplementary figures and images for: In silico analysis of phylogeny, structure, and function of arsenite oxidase from unculturable microbiome of arsenic contaminated soil
Source: J Genet Eng Biotechnol. 2021 Mar 29;19:47. doi: 10.1186/s43141-021-00146-x (PMC8006529; doi:10.1186/s43141-021-00146-x)

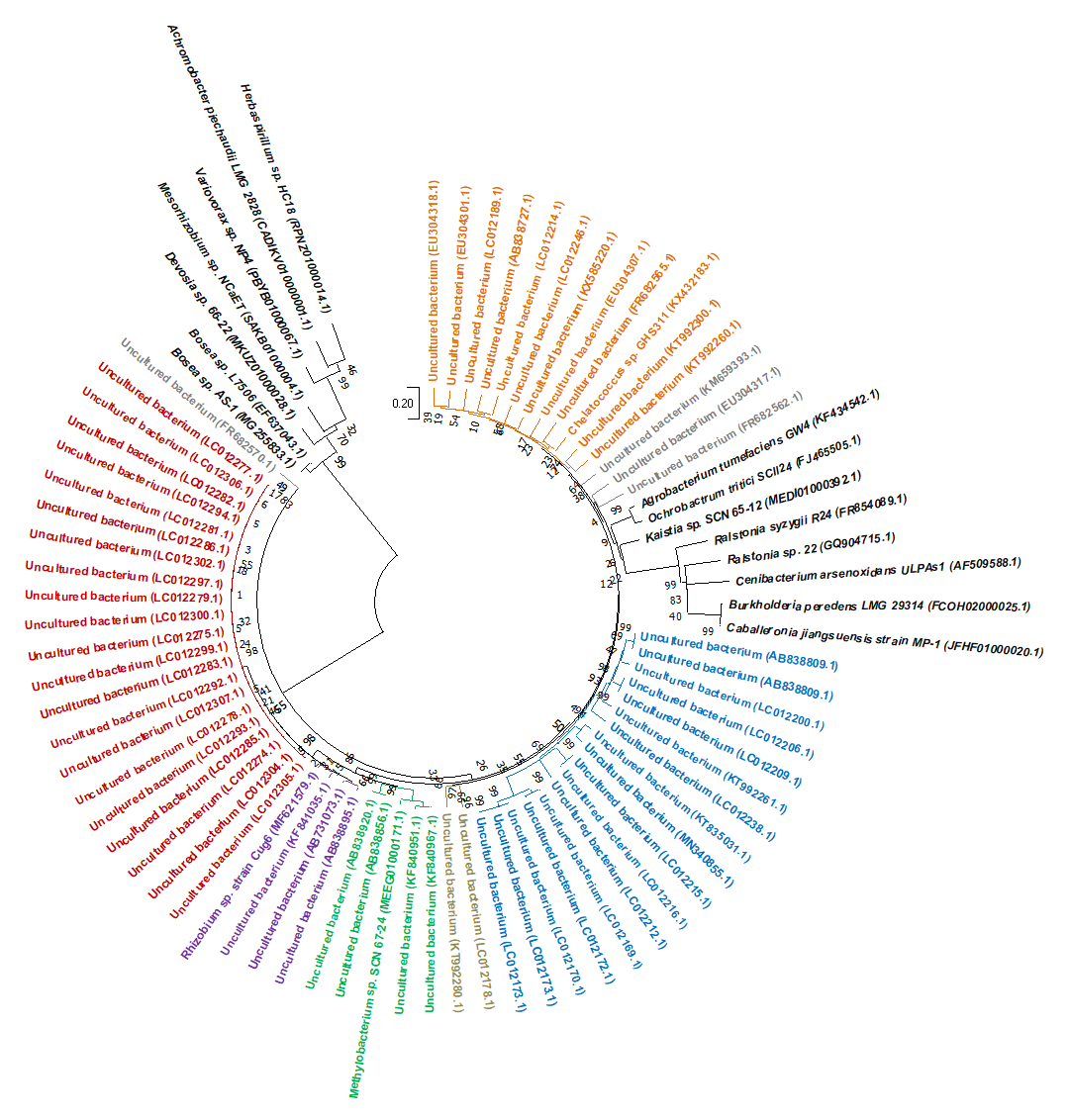

Supplement: Supplementary file 2 — Additional file 2. Secondary structure analysis of representative proteins. [file 43141_2021_146_MOESM2_ESM.tif]
