# Supplementary material for: In silico analysis of phylogeny, structure, and function of arsenite oxidase from unculturable microbiome of arsenic contaminated soil
Source: J Genet Eng Biotechnol. 2021 Mar 29;19:47. doi: 10.1186/s43141-021-00146-x (PMC8006529; doi:10.1186/s43141-021-00146-x)
Supplement: Supplementary file 3 — Additional file 3. Cofactors predicted by Cofactory 1.0 for query proteins. [file 43141_2021_146_MOESM3_ESM.pdf]

Name : AOS87703.1

Copy Link: http://bioinf.cs.ucl.ac.uk/psipr

Sequence Plot

Show psipred | Show memsat | Show aatypes

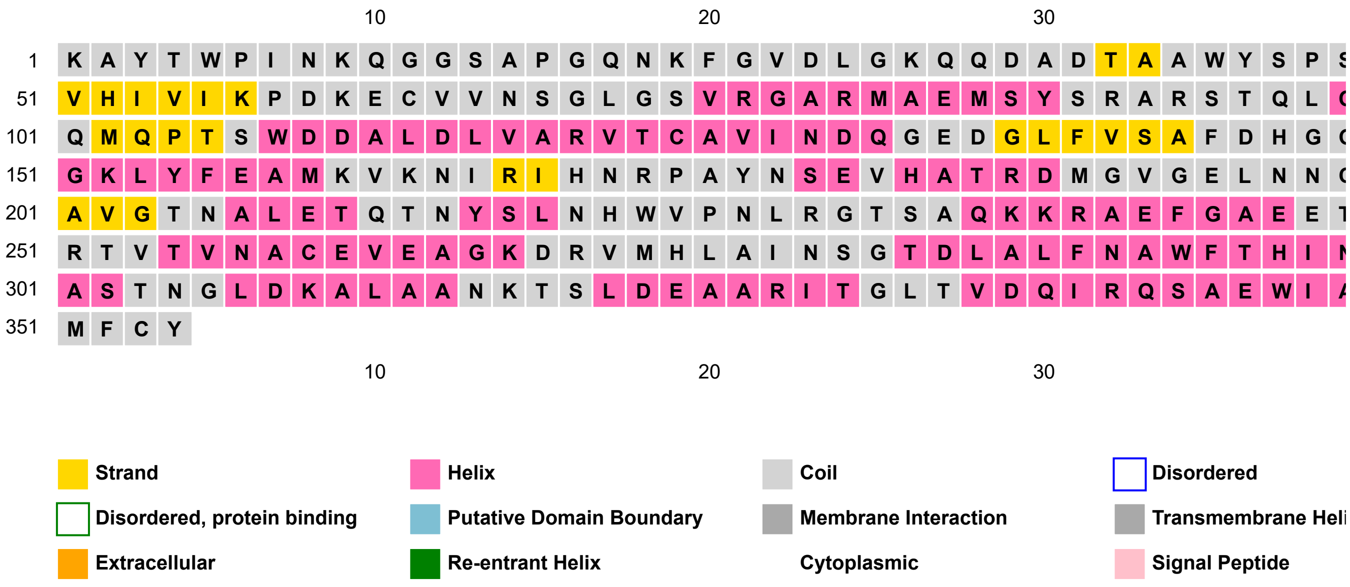

PSIPRED Cartoon

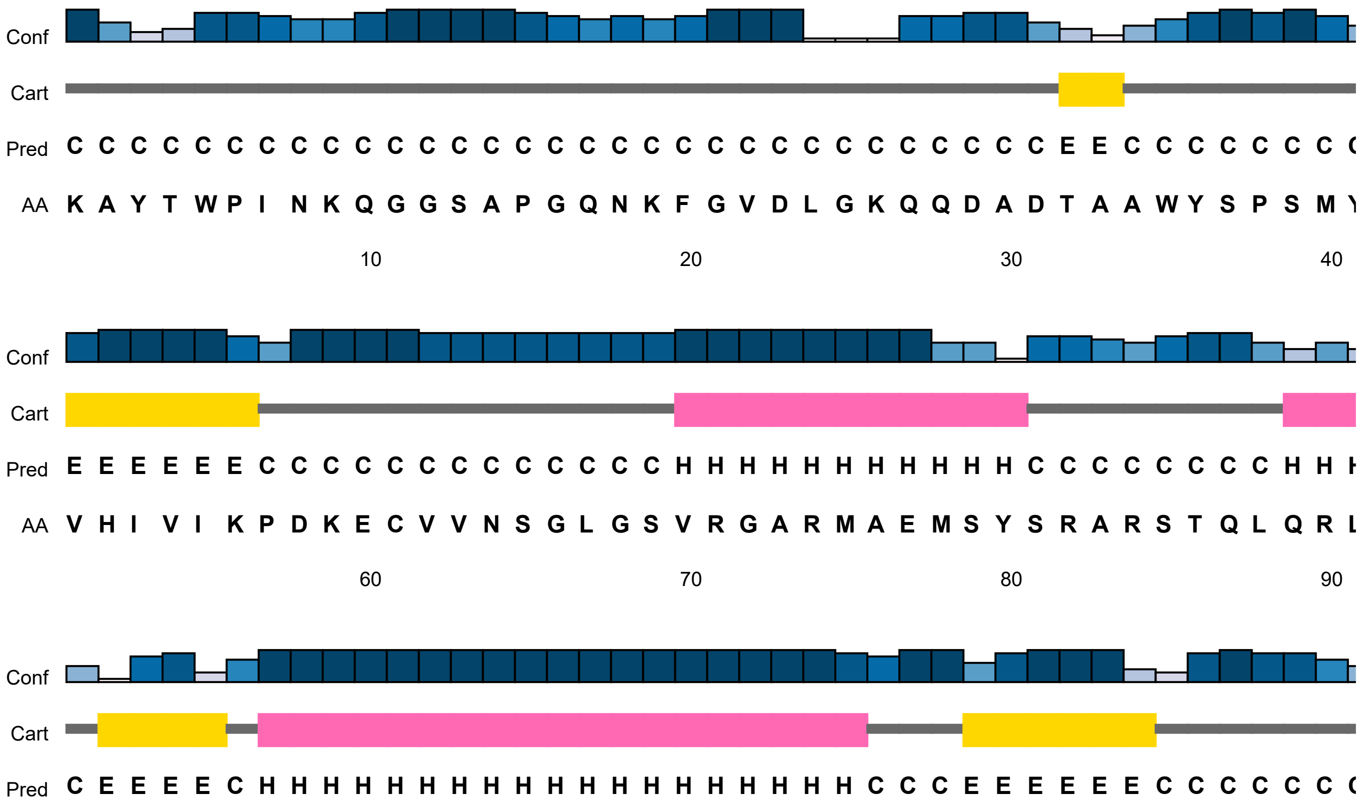

AA Q M Q P T S W D D A L D L V A R V T C A V I N D Q G E D G L F V S A F D H G G A

110 120 130 140

Conf

Cart

Pred H H H H H H H H C C C C C E E C C C C C C C H H C H H H H H C C C C C C C C C H

AA G K L Y F E A M K V K N I R I H N R P A Y N S E V H A T R D M G V G E L N N C Y

160 170 180 190

Conf

Cart

Pred E E E C C H H H H C C C H H H C C C C C C C C C C C C H H H H H H H H H H C C C

AA A V G T N A L E T Q T N Y S L N H W V P N L R G T S A Q K K R A E F G A E E T P

210 220 230 240

Conf

Cart

Pred C C C H H H H H H H H H H H C C C C C C C C C C C H H H H H H H H H H H H H H H

AA R T V T V N A C E V E A G K D R V M H L A I N S G T D L A L F N A W F T H I N E

260 270 280 290

Conf

Cart

Pred H H C C C H H H H H H H C C C C H H H H H H H H C C C H H H H H H H H H H H H C

AA A S T N G L D K A L A A N K T S L D E A A R I T G L T V D Q I R Q S A E W I A Q

310 320 330 340

Conf

Cart

Pred C C C C

AA M F C Y

360 370 380 390

Legend:

Strand
Helix
Coil

Conf: - + Confidence of prediction
Cart: 3-state assignment cartoon
Pred: 3-state prediction
AA: Target Sequence

Downloads −

ZIP FILE

Get Zip file ()

JOB CONFIGURATION

Get Job Details ()

PSIPRED V4.0 DOWNLOADS

Horiz Format Output (http://bioinf.cs.ucl.ac.uk/psipred/api/submissions/6403bd2c-a036-11ea-bca0-00163e100d53.horiz)
SS2 Format Output (http://bioinf.cs.ucl.ac.uk/psipred/api/submissions/6403bd2c-a036-11ea-bca0-00163e100d53.ss2)

Segment Resubmission

+

Name : Uncultured-2

Copy Link: http://bioinf.cs.ucl.ac.uk/psipr

Sequence Plot

Show psipred Show memsat Show aatypes

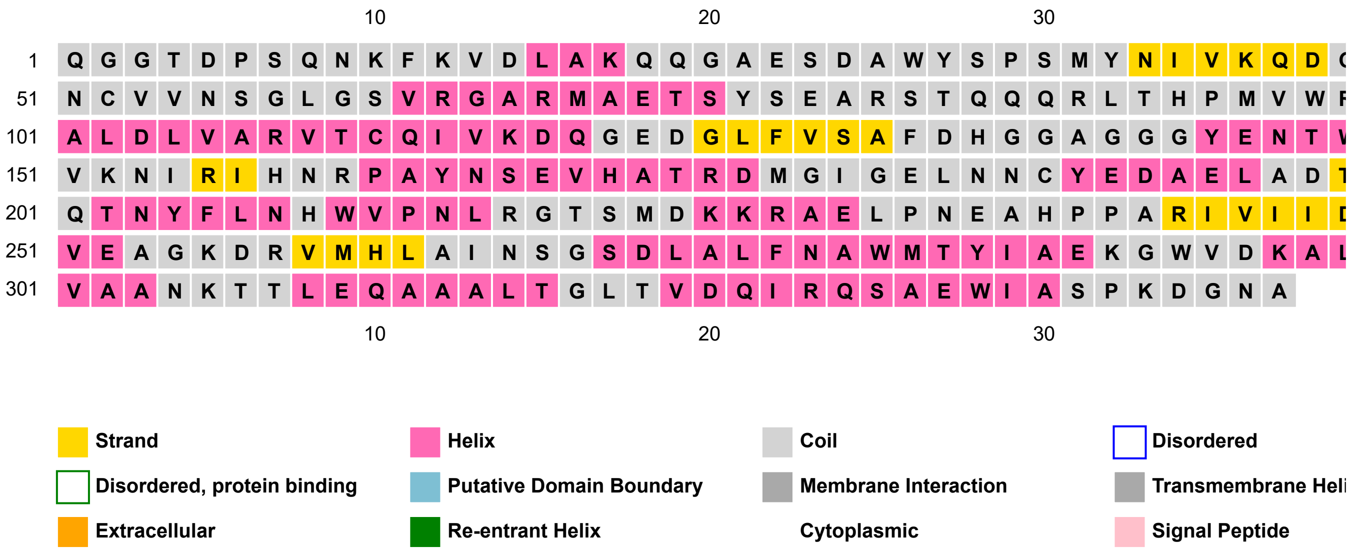

PSIPRED Cartoon

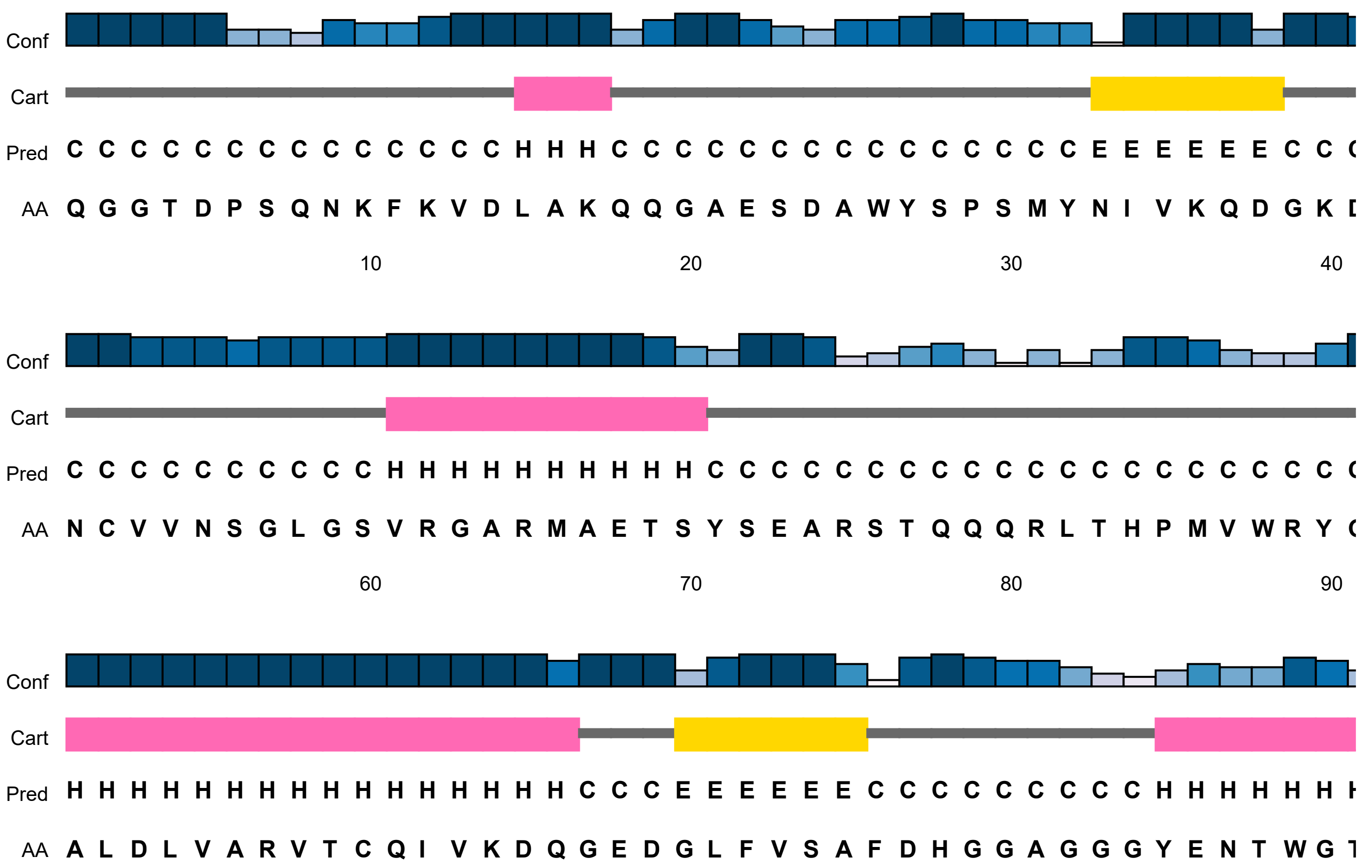

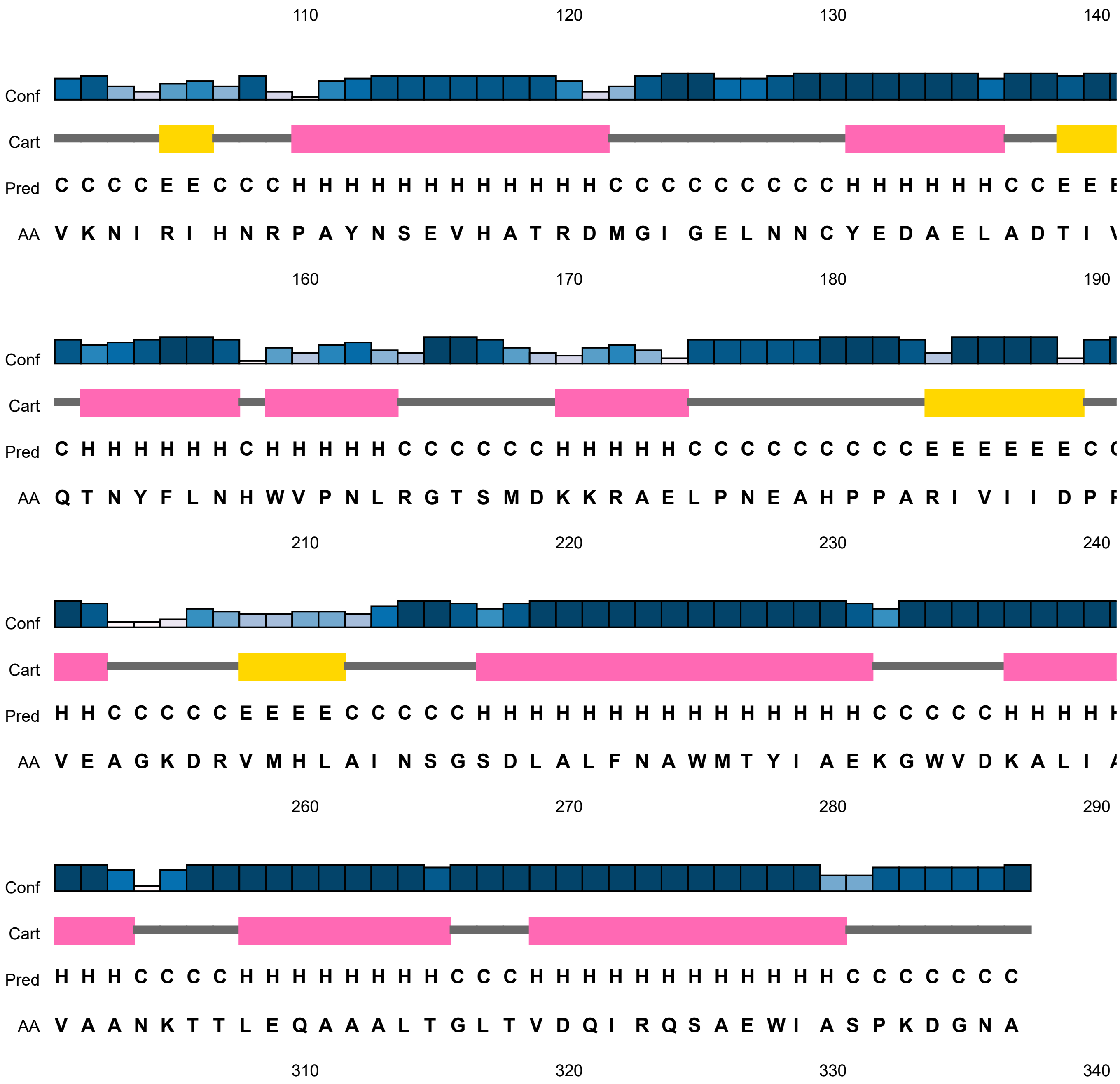

Legend:

- Strand
- Helix
- Coil

Conf: - + Confidence of prediction
Cart: 3-state assignment cartoon
Pred: 3-state prediction
AA: Target Sequence

Downloads

ZIP FILE

Get Zip file ()

JOB CONFIGURATION

Get Job Details ()

PSIPRED V4.0 DOWNLOADS

Horiz Format Output (http://bioinf.cs.ucl.ac.uk/psipred/api/submissions/8d154dea-a039-11ea-b84e-00163e100d53.horiz)
SS2 Format Output (http://bioinf.cs.ucl.ac.uk/psipred/api/submissions/8d154dea-a039-11ea-b84e-00163e100d53.ss2)

Segment Resubmission

+

Name : Uncultured-3

Copy Link: http://bioinf.cs.ucl.ac.uk/psipr

Sequence Plot

Show psipred | Show memsat | Show aatypes

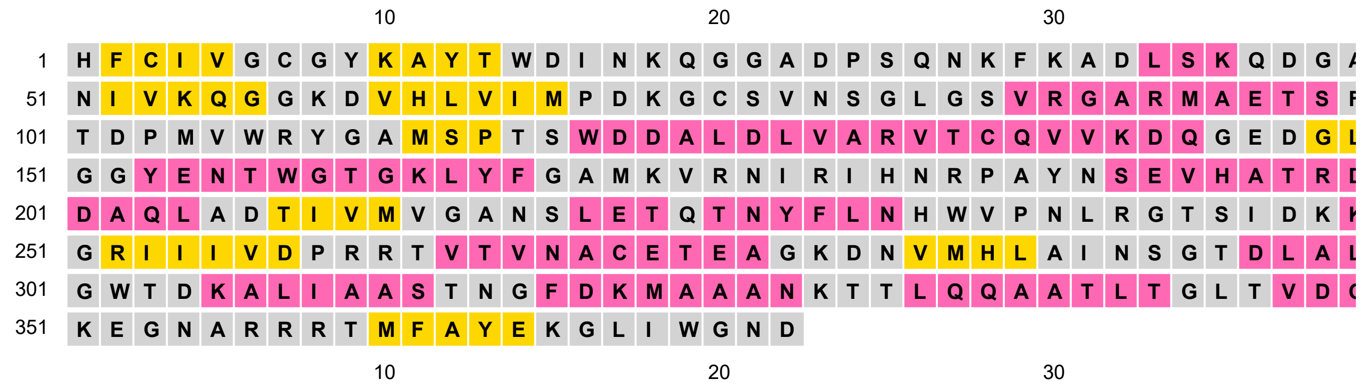

Strand | Helix | Coil | Disordered
Disordered, protein binding | Putative Domain Boundary | Membrane Interaction | Transmembrane Heli
Extracellular | Re-entrant Helix | Cytoplasmic | Signal Peptide

PSIPRED Cartoon

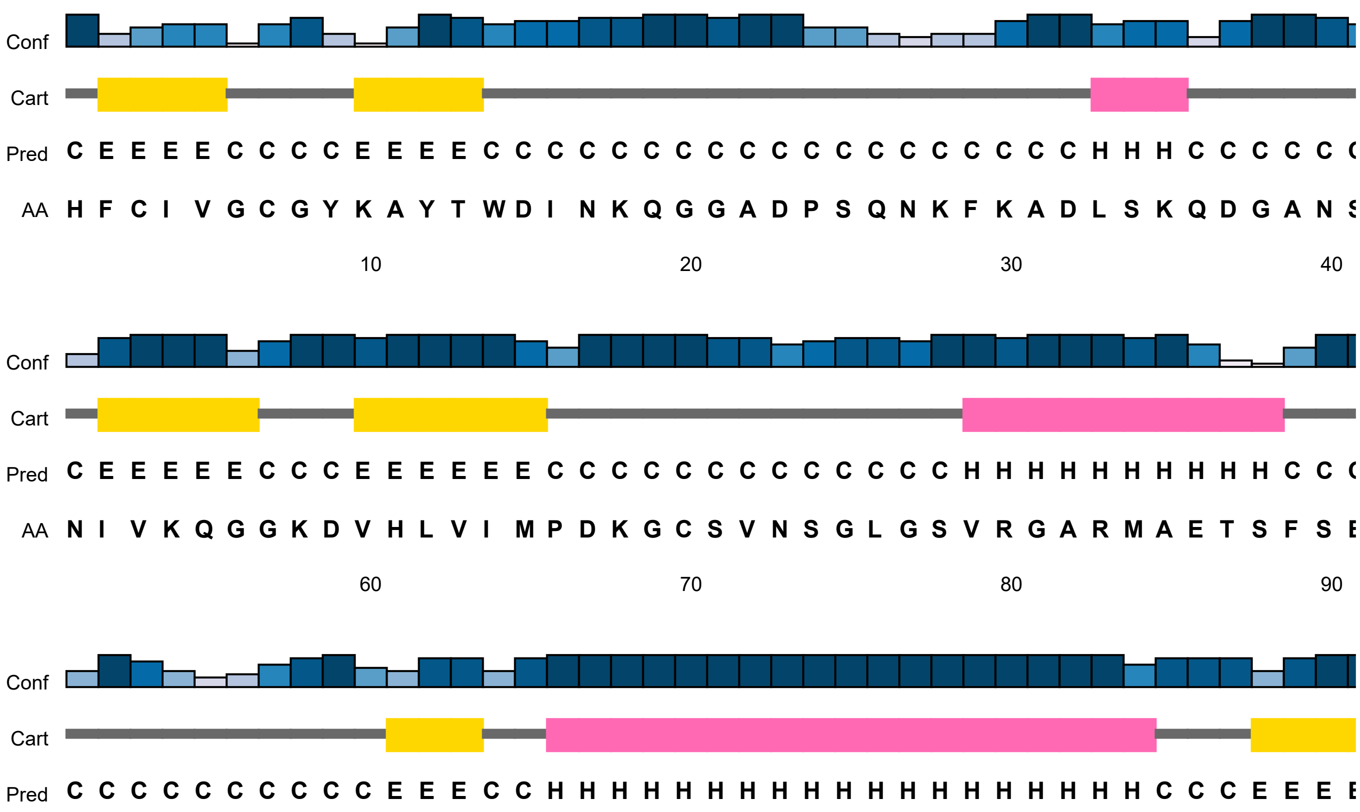

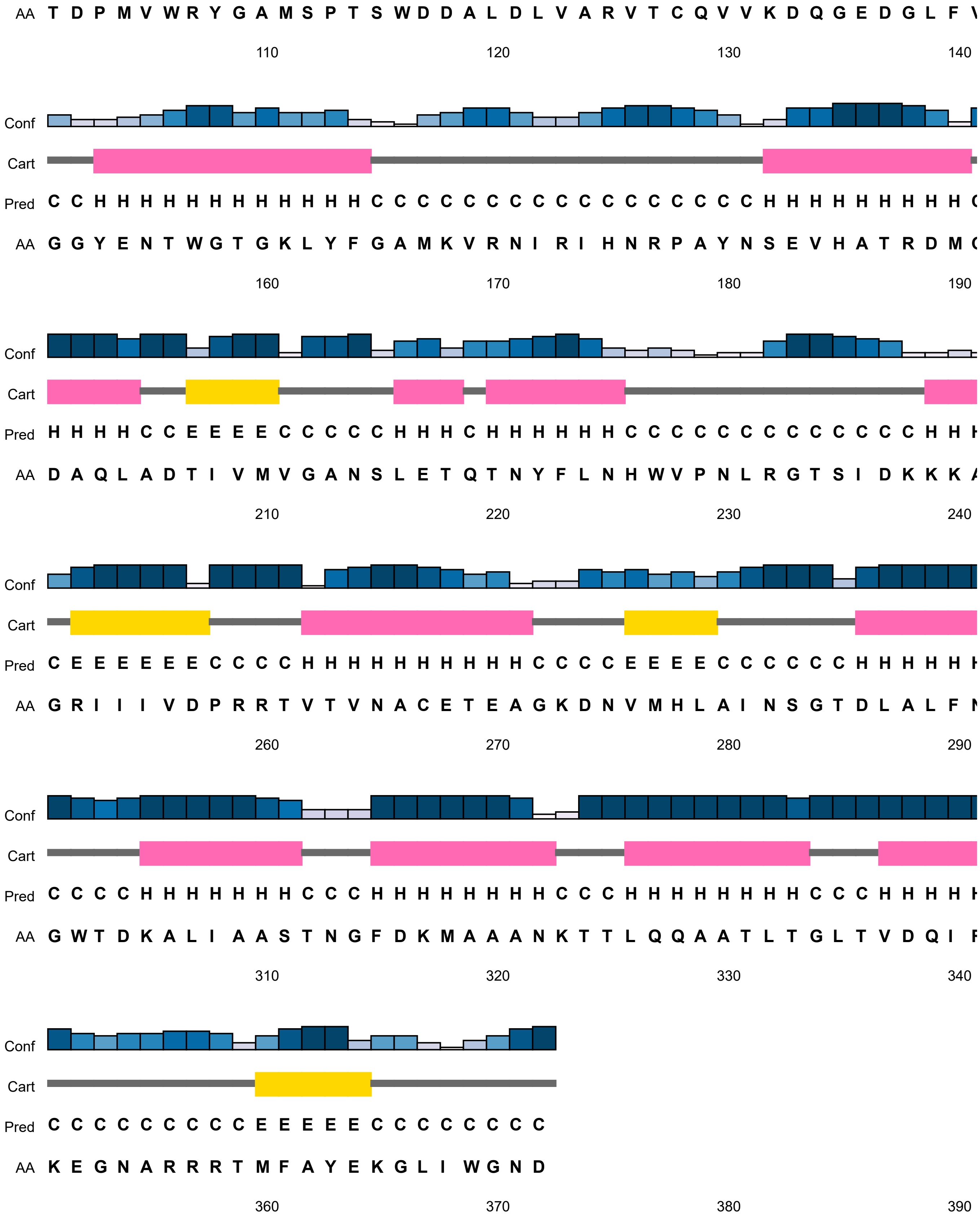

Legend:

Strand
Helix
Coil

Conf: - + Confidence of prediction
Cart: 3-state assignment cartoon
Pred: 3-state prediction
AA: Target Sequence

Downloads

ZIP FILE

Get Zip file ()

JOB CONFIGURATION

Get Job Details ()

PSIPRED V4.0 DOWNLOADS

Horiz Format Output (http://bioinf.cs.ucl.ac.uk/psipred/api/submissions/bfd7de70-a041-11ea-bcdc-00163e100d53.horiz)
SS2 Format Output (http://bioinf.cs.ucl.ac.uk/psipred/api/submissions/bfd7de70-a041-11ea-bcdc-00163e100d53.ss2)

Segment Resubmission

Home Blog Tools Academic Contact Mail

Target Sequence:

```
        10         20         30         40         50         60         70
KAYTWPINKQ GGSAPGQNKF GVDLGKQQDA DTAAWYSPSM YNIVRQNGED VHIVIKPDKE CVVNSGLGSV

        80         90        100        110        120        130        140
RGARMAEMSY SRARSTQLQR LTDPMVWRYG QMQPTSWDDA LDLVARVTCA VINDQGEDGL FVSAFDHGGA

       150        160        170        180        190        200        210
GGGYENTWGT GKLYFEAMKV KNIRIHNRPA YNSEVHATRD MGVGELNNCY EDAELADTIV AVGTNALETQ

       220        230        240        250        260        270        280
TNYSLNHWVP NLRGTSAQKK RAEFGAEETP PARIIIVDPR RTVTVNACEV EAGKDRVMHL AINSGTDLAL

       290        300        310        320        330        340        350
FNAWFTHINE RGWTDKAFIG ASTNGLDKAL AANKTSLDEA ARITGLTVDQ IRQSAEWIAQ PKAANARRRT

MFCY
```

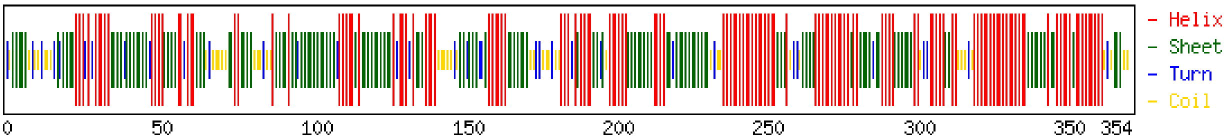

Secondary Structure:

```
                   *         *         *         *         *         *
Query 1   KAYTWPINKQGGSAPGQNKFGVDLGKQQDADTAAWYSPSMYNIVRQNGEDVHIVIKPDKECVVNSGLGSV 70
Helix 1       HH          HHHHHHHHHHHHHHHHHH           HHHHHHHHHHHHHHHHH         70
Sheet 1     EEEEE         EEEEEEEEEEE    EEEEEEEEEEEEEE     EEEE      EEE        70
Turns 1   T       T  T   T T       T T         T       T  T       T T     T      70
Struc 1   TCEEEEECTCCTCCCTETEEEEHHHTHTHHHHHEEEETEEEEEEETHHHHEEEEHHTHHHEEECTCCCCC 70

                   *         *         *         *         *         *
Query 71  RGARMAEMSYSRARSTQLQRLTDPMVWRYGQMQPTSWDDALDLVARVTCAVINDQGEDGLFVSAFDHGGA 140
Helix 71    HH          HHHHHH             HHHHHHHHHHHHHHHHHHHHHHHHHHHHHHHHH     140
Sheet 71  EEEEEEEE       EEEEEEEEEEEEEEEEEEEEE    EEEEEEEEEEEEEE     EEEEE       140
Turns 71             T          T           T   T              T   T             140
Struc 71  EEHHEEEECCCTCCHEEEEHEETEEEEEEEEEEETHHHHHEHEEEEEEEEEEHTHHHTHEEEHHHHCCCC 140

                   *         *         *         *         *         *
Query 141 GGGYENTWGTGKLYFEAMKVKNIRIHNRPAYNSEVHATRDMGVGELNNCYEDAELADTIVAVGTNALETQ 210
Helix 141            HHHHHHHHHHHHH           HHHHHHHHHH     HHHHHHHHHHHHHHHHHHH  210
Sheet 141    EEEEEEEEEEEEEEEEEEEEEE            EEEEEEEEEEEE     EEEEEEEEEEEEEEEE 210
Turns 141  T   T   TT                TT   T     T        T   T                   210
Struc 141 CTCEETEEETTEHHHHHHEEEEEEECCTTCCCTCCHHHTHEHHHHEETECHHHHHHEEEEEEEEHHHHEE 210

                   *         *         *         *         *         *
Query 211 TNYSLNHWVPNLRGTSAQKKRAEFGAEETPPARIIIVDPRRTVTVNACEVEAGKDRVMHLAINSGTDLAL 280
Helix 211                 HHHHHHHHHHHHHHHHHHHHH      HHHHHHHHHHHHHHHHHH     HHHH 280
Sheet 211 EEEEEEEEEEEE                     EEE     EEEEEE         EEEEEEEEEEEEEE 280
Turns 211 T            T    TT                  TT             TT        T       280
Struc 211 TEEEEEEEEEEECTCCHHHHHHHHHHHHHHHHHEEEHCTTCEEEEHHHHHHHHHHHEHHEEEETEEHHHH 280

                   *         *         *         *         *         *
Query 281 FNAWFTHINERGWTDKAFIGASTNGLDKALAANKTSLDEAARITGLTVDQIRQSAEWIAQPKAANARRRT 350
Helix 281 HHHHHHHH  HHHHHHHHHH     HHHHHHHHHHHHHHHHHHHHHHHHHHHHHHHHHHHHHHHHH     350
Sheet 281 EEEEEEE     EEEEEE                        EEEEEEEEEEEEEEEEE          E 350
Turns 281          TT   TT       T   T          T             T T     T      T   350
Struc 281 EEEEEEHHCTTHHHHHEEHHCCCTCHHHHHHHHHHHHHHHHHEEEEEEHEEHHHHHEHHHHHHHHHCTCE 350
```

```
Query 351 MFCY 354
Helix 351      354
Sheet 351 EE   354
Turns 351      354
Struc 351 EECC 354


Total Residues: H: 222   E: 203   T: 49
       Percent: H: 62.7  E: 57.3  T: 13.8
```

Copyright © 2009 - 2020 BioGem.Org. All Rights Reserved.

Home Blog Tools Academic Contact Mail

Target Sequence:

```
        10         20         30         40         50         60         70
QGGTDPSQNK FKVDLAKQQG AESDAWYSPS MYNIVKQDGK DVHVVIMPDK NCVVNSGLGS VRGARMAETS

        80         90        100        110        120        130        140
YSEARSTQQQ RLTHPMVWRY GAMSPTSWDD ALDLVARVTC QIVKDQGEDG LFVSAFDHGG AGGGYENTWG

       150        160        170        180        190        200        210
TGKLYFGAMK VKNIRIHNRP AYNSEVHATR DMGIGELNNC YEDAELADTI VVVGANPLET QTNYFLNHWV

       220        230        240        250        260        270        280
PNLRGTSMDK KRAELPNEAH PPARIVIIDP RRTVTVNACE VEAGKDRVMH LAINSGSDLA LFNAWMTYIA

       290        300        310        320        330
EKGWVDKALI AASTNGFDKM VAANKTTLEQ AAALTGLTVD QIRQSAEWIA SPKDGNA
```

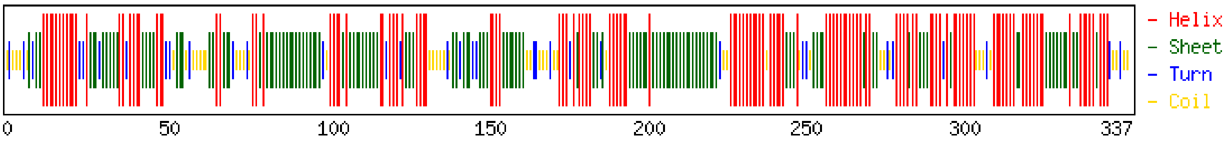

Secondary Structure:

```
                   *         *         *         *         *         *
Query 1   QGGTDPSQNKFKVDLAKQQGAESDAWYSPSMYNIVKQDGKDVHVVIMPDKNCVVNSGLGSVRGARMAETS 70
Helix 1          HHHHHHHHHHHHHHHHHH     HHHHHHHHHHHHHHHHHHH              HH      70
Sheet 1          EEEEEEEEEEE       EEEEEEEEEEE     EEEEE     EEE       EEEEEEEE  70
Turns 1    T   T  T        TT   TT    T       T  T        TT     T            T  70
Struc 1   CTCCCTCETEEHHHHHHHHHHHTTHEEETEEEEEHHTHHHHEEEEHHHTTCEEECTCCCCCEEHHEEETC 70

                   *         *         *         *         *         *
Query 71  YSEARSTQQQRLTHPMVWRYGAMSPTSWDDALDLVARVTCQIVKDQGEDGLFVSAFDHGGAGGGYENTWG 140
Helix 71      HHHHHHH                HHHHHHHHHHHHHHHHHHHHHHHHHHHHHH              140
Sheet 71        EEEEEEEEEEEEEEEEEEE      EEEEEEEEEEEEEE     EEEEE         EEEEEE 140
Turns 71    T     TT               T   T              T   T             T   T    140
Struc 71  CCTCHHEHEEEEEEEEEEEEEEEEETCHHHHEHEEEEEEEEEHHTHHHTHEEEHHHHCCCCCTCEETEEE 140

                   *         *         *         *         *         *
Query 141 TGKLYFGAMKVKNIRIHNRPAYNSEVHATRDMGIGELNNCYEDAELADTIVVVGANPLETQTNYFLNHWV 210
Helix 141   HHHHHHHHHHHHH           HHHHHHHHHH     HHHHHHHHHHHHH          HH     210
Sheet 141 EEEEEEEEEEEEEEEE            EEEEEE EEEEE     EEEEEEEEEEEEEEEEEEEEEEEEE 210
Turns 141 TT                TT   T     T        T   T                  T         210
Struc 141 TTEEEHHHHEEEEEEECCTTCCCTCCHHHTHEHHHHEETECHHHHHHEEEEEEHEEEEEEEEEEEEEEEE 210

                   *         *         *         *         *         *
Query 211 PNLRGTSMDKKRAELPNEAHPPARIVIIDPRRTVTVNACEVEAGKDRVMHLAINSGSDLALFNAWMTYIA 280
Helix 211        HHHHHHHHHHH HHHHHHHHH      HHHHHHHHHHHHHHHHHH     HHHHHHHHHHHHH 280
Sheet 211 EEEEE                   EEE     EEEEEE         EEEEE      EEEEEEEEEE   280
Turns 211     T    TT     T            TT             TT        T T              280
Struc 211 EEEETCCHHHHHHHHHHHCHHHHHEEEHCTTCEEEEHHHHHHHHHHHEHHEECCTCTHHHHEHHEEEHHH 280

                   *         *         *         *         *
Query 281 EKGWVDKALIAASTNGFDKMVAANKTTLEQAAALTGLTVDQIRQSAEWIASPKDGNA 337
Helix 281 HHHHHHHHHHH     HHHHHHHHHHHHHHHHHHHHHHHHHHHHHHHHHHHH      337
Sheet 281    EEEEEEE      EEEEEEEEEEEEEEEEEEEEEEEEEEEEEEEE         337
Turns 281  T    T       T              T             T T     T  T   337
Struc 281 HTHEHHHHHHHCCCTCHHHHHHHEEHHHHHHHEEEEEEEHEEHHHHHEHHHTCCTCC 337


Total Residues: H: 212   E: 202   T: 50
       Percent: H: 62.9  E: 59.9  T: 14.8
```

Copyright © 2009 - 2020 BioGem.Org. All Rights Reserved.

Target Sequence:

```
        10         20         30         40         50         60         70
HFCIVGCGYK AYTWDINKQG GADPSQNKFK ADLSKQDGAN SDAWYSPSMH NIVKQGGKDV HLVIMPDKGC

        80         90        100        110        120        130        140
SVNSGLGSVR GARMAETSFS EARSTQAQRL TDPMVWRYGA MSPTSWDDAL DLVARVTCQV VKDQGEDGLF

       150        160        170        180        190        200        210
VSAFDHGGAG GGYENTWGTG KLYFGAMKVR NIRIHNRPAY NSEVHATRDM GVGELNNCYE DAQLADTIVM

       220        230        240        250        260        270        280
VGANSLETQT NYFLNHWVPN LRGTSIDKKK AELPNEPHAA GRIIIVDPRR TVTVNACETE AGKDNVMHLA

       290        300        310        320        330        340        350
INSGTDLALF NAWMTYIAEK GWTDKALIAA STNGFDKMAA ANKTTLQQAA TLTGLTVDQI RQSAEWIAMP

       360        370
KEGNARRRTM FAYEKGLIWG ND
```

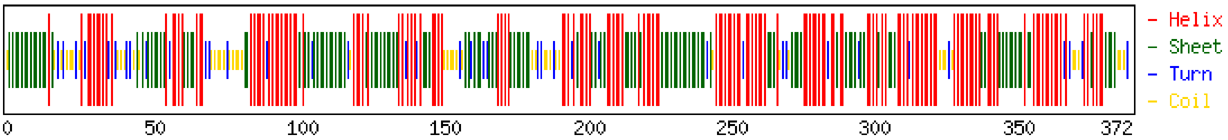

Secondary Structure:

```
                   *         *         *         *         *         *
Query 1   HFCIVGCGYKAYTWDINKQGGADPSQNKFKADLSKQDGANSDAWYSPSMHNIVKQGGKDVHLVIMPDKGC 70
Helix 1                HH          HHHHHHHHHHH            HHHHHHHHHHHHHHHHHHH    70
Sheet 1    EEEEEEEEEEEEEEE                           E EEEEEEEEE     EEEEE       70
Turns 1                    T T   T  T   T   T T   TT    T       T  T        TT   70
Struc 1   CEEEEEEEEEEEEEHECTCTCCCTCHTHHHHHHHTHTCCCTTCECETEEEEEEHTHHHHEEEEHHHTTCC 70

                   *         *         *         *         *         *
Query 71  SVNSGLGSVRGARMAETSFSEARSTQAQRLTDPMVWRYGAMSPTSWDDALDLVARVTCQVVKDQGEDGLF 140
Helix 71             HHHHHHHHHHHHHHHHHH                HHHHHHHHHHHHHHHHHHHHHHHHH 140
Sheet 71           EEEEEEEE       EEEEEEEEEEEEEEEEEEE      EEEEEEEEEEEEEE     EE 140
Turns 71     T            T   T          T           T   T              T   T    140
Struc 71  CCCTCCCCCEEHHHHHTHHHHHHHHHHEHEETEEEEEEEEEEETCHHHHEHEEEEEEEEEEHHTHHHTHEE 140

                   *         *         *         *         *         *
Query 141 VSAFDHGGAGGGYENTWGTGKLYFGAMKVRNIRIHNRPAYNSEVHATRDMGVGELNNCYEDAQLADTIVM 210
Helix 141 HHHHH               HHHHHHHHHHHH            HHHHHHHHHH     HHHHHHHHHHH 210
Sheet 141 EEE         EEEEEEEEEEEEEEEEEEEEEE            EEEEEEEEEEEEEEEEEEEEEEEE 210
Turns 141           T   T   TT                 TT   T     T        T   T         210
Struc 141 EHHHHCCCCCTCEETEEETTEEEHHHHEEEEEEEECCTTCCCTCCHHHTHEHHHHEETEEHHHHHHEEEEH 210

                   *         *         *         *         *         *
Query 211 VGANSLETQTNYFLNHWVPNLRGTSIDKKKAELPNEPHAAGRIIIVDPRRTVTVNACETEAGKDNVMHLA 280
Helix 211 HHHHHHH     HH           HHHHHHHHHHHHHHHHHHHHH      HHHHHHHHHHHHHHHHHH 280
Sheet 211 EEEEEEEEEEEEEEEEEEEEEEEE                  EEE     EEEEEE         EEEEE 280
Turns 211          T            T    TT     TT    T      TT             TT       280
Struc 211 EHHHHHHEEEEEEEEEEEEEEEETECHHHHHHHHHTHHHHHHHEEEHCTTCEEEEHHHHHHHHTHHEHHEE 280

                   *         *         *         *         *         *
Query 281 INSGTDLALFNAWMTYIAEKGWTDKALIAASTNGFDKMAAANKTTLQQAATLTGLTVDQIRQSAEWIAMP 350
Helix 281      HHHHHHHHHHHHHHHHHHHHHHHHH    HHHHHHHHHHHHHHHHHHHHHHHHHHHHHHHHHHHH 350
Sheet 281 EEEEEEEEEEEEEEEE     EEEEEEE               EEEEEEEEEEEEEEEEEEEEEEEEE   350
Turns 281   T                T   TT       T              T             T T       350
Struc 281 EETEEHHHHEHHEEEHHHHTHHHHHHHHHCCCTCHHHHHHHHHEEHHHHEEEEEEEEEHEEHHHHHHHHHH 350

                   *         *
```

```
Query 351 KEGNARRRTMFAYEKGLIWGND 372
Helix 351 HH     HHHHHHHHHHH     372
Sheet 351         EEEEEEEEEE     372
Turns 351 T T   T       T      T 372
Struc 351 THTCCCTHHEHHHHEEEECCCT 372


Total Residues: H: 234   E: 218   T: 56
       Percent: H: 62.9  E: 58.6  T: 15.1
```

Copyright © 2009 - 2020 BioGem.Org. All Rights Reserved.
