# Supplementary material for: In silico analysis of phylogeny, structure, and function of arsenite oxidase from unculturable microbiome of arsenic contaminated soil
Source: J Genet Eng Biotechnol. 2021 Mar 29;19:47. doi: 10.1186/s43141-021-00146-x (PMC8006529; doi:10.1186/s43141-021-00146-x)
Supplement: Supplementary file 4 — Additional file 4. Homology modelling of representative enzyme obtained from SWISS MODEL. [file 43141_2021_146_MOESM4_ESM.pdf]

DTU Bioinformatics
Department of Bio and Health Informatics

Services are gradually being migrated to https://services.healthtech.dtu.dk/.
Please try out the new site.

Home

Output format

Short description:

Each identified Rossmann fold sequence domain is associated with three neural network scores, one for each of the three cofactors FAD, NAD and NADP. A score above 0.5 indicates that the domain is predicted to be specific for the particular cofactor. The prediction scores are followed by a summary of the predicted cofactor specificity. If multiple specificities are predicted the identifiers are separated with a slash e.g. NAD/NADP. The approximate Rossmann fold sequence boundaries are provided next to the summary and these are followed by the amino acid sequence. If no Rossmann fold sequence domains are identified the domain count is 0 and no scores or domain boundaries are reported.

Example:

```
# SEQUENCE ID             Domain FAD   NAD   NADP  Cofactor(s)  From  To    Sequence
Input_1                   1      0.670 0.387 0.585 FAD/NADP     5     46    SQKRVVVLGSGVIGLSSALILARKGYSVHILARDLPEDVSSQ
Input_2                   1      0.765 0.258 0.073 FAD          1     48    MRVVVIGAGVIGLSTALCIHERYHSVLQPLDVKVYADRFTPFTTTDVA
Input_3                   1      0.939 0.228 0.169 FAD          1     43    MKVIVLGSSHGGYEAVEELLNLHPDAEIQWYEKGDFISFLSGM
Input_3                   2      0.502 0.837 0.063 FAD/NAD      147   185   EVNNVVVIGSGYIGIEAAEAFAKAGKKVTVIDILDRPLG
Input_4                   0      -     -     -     -            -     -     -
```

GETTING HELP

Scientific problems: Henrik Marcus Geertz-Hansen   Technical problems: Thomas Nordahl Petersen

---

This file was last modified Wednesday 6th 2013f November 2013 23:01:46 GMT

DTU Bioinformatics
Department of Bio and Health Informatics

Services are gradually being migrated to https://services.healthtech.dtu.dk/. Please try out the new site.

Home

Output format

Short description:

Each identified Rossmann fold sequence domain is associated with three neural network scores, one for each of the three cofactors FAD, NAD and NADP. A score above 0.5 indicates that the domain is predicted to be specific for the particular cofactor. The prediction scores are followed by a summary of the predicted cofactor specificity. If multiple specificities are predicted the identifiers are separated with a slash e.g. NAD/NADP. The approximate Rossmann fold sequence boundaries are provided next to the summary and these are followed by the amino acid sequence. If no Rossmann fold sequence domains are identified the domain count is 0 and no scores or domain boundaries are reported.

Example:

```
# SEQUENCE ID             Domain FAD   NAD   NADP  Cofactor(s)  From  To    Sequence
Input_1                   1      0.670 0.387 0.585 FAD/NADP     5     46    SQKRVVVLGSGVIGLSSALILARKGYSVHILARDLPEDVSSQ
Input_2                   1      0.765 0.258 0.073 FAD          1     48    MRVVVIGAGVIGLSTALCIHERYHSVLQPLDVKVYADRFTPFTTTDVA
Input_3                   1      0.939 0.228 0.169 FAD          1     43    MKVIVLGSSHGGYEAVEELLNLHPDAEIQWYEKGDFISFLSGM
Input_3                   2      0.502 0.837 0.063 FAD/NAD      147   185   EVNNVVVIGSGYIGIEAAEAFAKAGKKVTVIDILDRPLG
Input_4                   0      -     -     -     -            -     -     -
```

GETTING HELP

Scientific problems: Henrik Marcus Geertz-Hansen   Technical problems: Thomas Nordahl Petersen

This file was last modified Wednesday 6th 2013f November 2013 23:01:46 GMT

DTU Bioinformatics
Department of Bio and Health Informatics

Services are gradually being migrated to https://services.healthtech.dtu.dk/.
Please try out the new site.

Home

Output format

Short description:

Each identified Rossmann fold sequence domain is associated with three neural network scores, one for each of the three cofactors FAD, NAD and NADP. A score above 0.5 indicates that the domain is predicted to be specific for the particular cofactor. The prediction scores are followed by a summary of the predicted cofactor specificity. If multiple specificities are predicted the identifiers are separated with a slash e.g. NAD/NADP. The approximate Rossmann fold sequence boundaries are provided next to the summary and these are followed by the amino acid sequence. If no Rossmann fold sequence domains are identified the domain count is 0 and no scores or domain boundaries are reported.

Example:

```
# SEQUENCE ID               Domain FAD   NAD   NADP  Cofactor(s)  From  To    Sequence
Input_1                     1      0.670 0.387 0.585 FAD/NADP     5     46    SQKRVVVLGSGVIGLSSALILARKGYSVHILARDLPEDVSSQ
Input_2                     1      0.765 0.258 0.073 FAD          1     48    MRVVVIGAGVIGLSTALCIHERYHSVLQPLDVKVYADRFTPFTTTDVA
Input_3                     1      0.939 0.228 0.169 FAD          1     43    MKVIVLGSSHGGYEAVEELLNLHPDAEIQWYEKGDFISFLSGM
Input_3                     2      0.502 0.837 0.063 FAD/NAD      147   185   EVNNVVVIGSGYIGIEAAEAFAKAGKKVTVIDILDRPLG
Input_4                     0      -     -     -     -            -     -     -
```

GETTING HELP

Scientific problems: Henrik Marcus Geertz-Hansen   Technical problems: Thomas Nordahl Petersen

---

This file was last modified Wednesday 6th 2013f November 2013 23:01:46 GMT
