# Supplementary material for: In silico analysis of phylogeny, structure, and function of arsenite oxidase from unculturable microbiome of arsenic contaminated soil
Source: J Genet Eng Biotechnol. 2021 Mar 29;19:47. doi: 10.1186/s43141-021-00146-x (PMC8006529; doi:10.1186/s43141-021-00146-x)
Supplement: Supplementary file 5 — Additional file 5. Evaluation reports predicted protein models of representative enzyme obtained from SAVES server. [file 43141_2021_146_MOESM5_ESM.pdf]

SWISS-MODEL Homology Modelling Report

Model Building Report

This document lists the results for the homology modelling project "Untitled Project" submitted to SWISS-MODEL workspace on May 28, 2020, 1:25 p.m..The submitted primary amino acid sequence is given in Table T1.

If you use any results in your research, please cite the relevant publications:

- Waterhouse, A., Bertoni, M., Bienert, S., Studer, G., Tauriello, G., Gumienny, R., Heer, F.T., de Beer, T.A.P., Rempfer, C., Bordoli, L., Lepore, R., Schwede, T. SWISS-MODEL: homology modelling of protein structures and complexes. Nucleic Acids Res. 46(W1), W296-W303 (2018). doi>
- Guex, N., Peitsch, M.C., Schwede, T. Automated comparative protein structure modeling with SWISS-MODEL and Swiss-PdbViewer: A historical perspective. Electrophoresis 30, S162-S173 (2009). doi>
- Bienert, S., Waterhouse, A., de Beer, T.A.P., Tauriello, G., Studer, G., Bordoli, L., Schwede, T. The SWISS-MODEL Repository - new features and functionality. Nucleic Acids Res. 45, D313-D319 (2017). doi>
- Studer, G., Rempfer, C., Waterhouse, A.M., Gumienny, G., Haas, J., Schwede, T. QMEANDisCo - distance constraints applied on model quality estimation. Bioinformatics 36, 1765-1771 (2020). doi>
- Bertoni, M., Kiefer, F., Biasini, M., Bordoli, L., Schwede, T. Modeling protein quaternary structure of homo- and hetero-oligomers beyond binary interactions by homology. Scientific Reports 7 (2017). doi>

Results

The SWISS-MODEL template library (SMTL version 2020-05-27, PDB release 2020-05-22) was searched with BLAST (Camacho et al.) and HHBlits (Remmert et al.) for evolutionary related structures matching the target sequence in Table T1. For details on the template search, see Materials and Methods. Overall 162 templates were found (Table T2).

Models

The following model was built (see Materials and Methods "Model Building"):

Model #01	File	Built with	Oligo-State	Ligands	GMQE	QMEAN
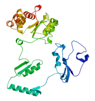	PDB	ProMod3 3.0.0	monomer (matching prediction)	None	0.89	-1.78

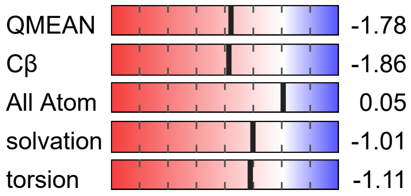

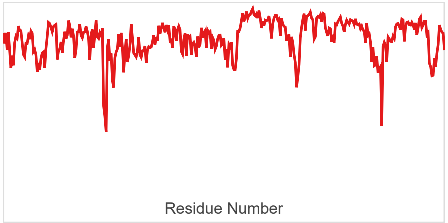

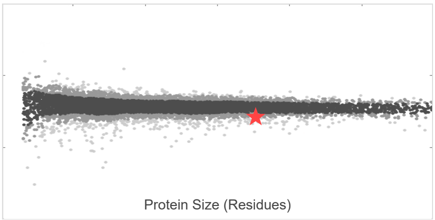

Template	Seq Identity	Oligo-state	QSQE	Found by	Method	Resolution	Seq Similarity	Range	Coverage	Description
5nqd.1.A	73.94	homo-dimer	0.69	BLAST	X-ray	2.20Å	0.53	2 - 354	1.00	AroA

Excluded ligands

Ligand Name.Number	Reason for Exclusion	Description
4MO.4	Binding site not conserved.	MOLYBDENUM(IV) ION
4MO.17	Binding site not conserved.	MOLYBDENUM(IV) ION
EDO.13	Not biologically relevant.	1,2-ETHANEDIOL
F3S.5	Binding site not conserved.	FE3-S4 CLUSTER
F3S.18	Binding site not conserved.	FE3-S4 CLUSTER
FES.12	Binding site not conserved.	FE2/S2 (INORGANIC) CLUSTER

Ligand Name.Number	Reason for Exclusion	Description
FES.22	Binding site not conserved.	FE2/S2 (INORGANIC) CLUSTER
GOL.11	Not biologically relevant.	GLYCEROL
GOL.21	Not biologically relevant.	GLYCEROL
MGD.1	Binding site not conserved.	2-AMINO-5,6-DIMERCAPTO-7-METHYL-3,7,8A,9-TETRAHYDRO-8-OXA-1,3,9,10-TETRAAZA-ANTHRACEN-4-ONE GUANOSINE DINUCLEOTIDE
MGD.2	Binding site not conserved.	2-AMINO-5,6-DIMERCAPTO-7-METHYL-3,7,8A,9-TETRAHYDRO-8-OXA-1,3,9,10-TETRAAZA-ANTHRACEN-4-ONE GUANOSINE DINUCLEOTIDE
MGD.14	Binding site not conserved.	2-AMINO-5,6-DIMERCAPTO-7-METHYL-3,7,8A,9-TETRAHYDRO-8-OXA-1,3,9,10-TETRAAZA-ANTHRACEN-4-ONE GUANOSINE DINUCLEOTIDE
MGD.15	Binding site not conserved.	2-AMINO-5,6-DIMERCAPTO-7-METHYL-3,7,8A,9-TETRAHYDRO-8-OXA-1,3,9,10-TETRAAZA-ANTHRACEN-4-ONE GUANOSINE DINUCLEOTIDE
O.3	Binding site not conserved.	OXYGEN ATOM
O.16	Binding site not conserved.	OXYGEN ATOM
PGE.10	Not biologically relevant.	TRIETHYLENE GLYCOL
SO4.6	Not biologically relevant.	SULFATE ION
SO4.7	Not biologically relevant.	SULFATE ION
SO4.8	Not biologically relevant.	SULFATE ION
SO4.9	Not biologically relevant.	SULFATE ION
SO4.19	Not biologically relevant.	SULFATE ION
SO4.20	Not biologically relevant.	SULFATE ION

```
Target     KAYTWPINKQGGSAPGQNKFGVDLGKQQDADTAAWYSPSMYNIVRQNGEDVHIVIKPDKECVVNSGLGSVRGARMAEMSY
5nqd.1.A   -AYTWPINKQGGTDPQNNIFGVDLSEQQQAESDAWYSPSMYNVVKQDGRDVHVVIKPDHECVVNSGLGSVRGARMAETSF

Target     SRARSTQLQRLTDPMVWRYGQMQPTSWDDALDLVARVTCAVINDQGEDGLFVSAFDHGGAGGGYENTWGTGKLYFEAMKV
5nqd.1.A   SEARNTQQQRLTDPLVWRYGQMQPTSWDDALDLVARVTAKIVKEKGEDALIVSAFDHGGAGGGYENTWGTGKLYFEAMKV

Target     KNIRIHNRPAYNSEVHATRDMGVGELNNCYEDAELADTIVAVGTNALETQTNYSLNHWVPNLRGTSAQKKRAEFGAEETP
5nqd.1.A   KNIRIHNRPAYNSEVHGTRDMGVGELNNCYEDAELADTIVAVGTNALETQTNYFLNHWIPNLRGESLGKKKELMPEEPHE

Target     PARIIIVDPRRTVTVNACEVEAGKDRVMHLAINSGTDLALFNAWFTHINERGWTDKAFIGAST-----------------
5nqd.1.A   AGRIIIVDPRRTVTVNACEQTAGADNVLHLAINSGTDLALFNALFTYIADKGWVDRDFIDKSTLREGTARPPLYPARGVS

Target     -------NGLDKALAANKTSLDEAARITGLTVDQIRQSAEWIAQPKAANARRRTMFCY
5nqd.1.A   EANPGHLSSFEDAVEGCRMSIEEAAEITGLDAAQIIKAAEWIGMPKEGGKRRRVMFGY
```

Materials and Methods

Template Search

Template search with BLAST and HHBlits has been performed against the SWISS-MODEL template library (SMTL, last update: 2020-05-27, last included PDB release: 2020-05-22).

The target sequence was searched with BLAST against the primary amino acid sequence contained in the SMTL. A total of 13 templates were found.

An initial HHblits profile has been built using the procedure outlined in (Remmert et al.), followed by 1 iteration of HHblits against NR20. The obtained profile has then be searched against all profiles of the SMTL. A total of 150 templates were

found.

Model Building

Models are built based on the target-template alignment using ProMod3. Coordinates which are conserved between the target and the template are copied from the template to the model. Insertions and deletions are remodelled using a fragment library. Side chains are then rebuilt. Finally, the geometry of the resulting model is regularized by using a force field. In case loop modelling with ProMod3 fails, an alternative model is built with PROMOD-II (Guex et al.).

Model Quality Estimation

The global and per-residue model quality has been assessed using the QMEAN scoring function (Studer et al.).

Ligand Modelling

Ligands present in the template structure are transferred by homology to the model when the following criteria are met: (a) The ligands are annotated as biologically relevant in the template library, (b) the ligand is in contact with the model, (c) the ligand is not clashing with the protein, (d) the residues in contact with the ligand are conserved between the target and the template. If any of these four criteria is not satisfied, a certain ligand will not be included in the model. The model summary includes information on why and which ligand has not been included.

Oligomeric State Conservation

The quaternary structure annotation of the template is used to model the target sequence in its oligomeric form. The method (Bertoni et al.) is based on a supervised machine learning algorithm, Support Vector Machines (SVM), which combines interface conservation, structural clustering, and other template features to provide a quaternary structure quality estimate (QSQE). The QSQE score is a number between 0 and 1, reflecting the expected accuracy of the interchain contacts for a model built based a given alignment and template. Higher numbers indicate higher reliability. This complements the GMQE score which estimates the accuracy of the tertiary structure of the resulting model.

References

- **BLAST**
  Camacho, C., Coulouris, G., Avagyan, V., Ma, N., Papadopoulos, J., Bealer, K., Madden, T.L. BLAST+: architecture and applications. BMC Bioinformatics 10, 421-430 (2009). doi>
- **HHblits**
  Remmert, M., Biegert, A., Hauser, A., Söding, J. HHblits: lightning-fast iterative protein sequence searching by HMM-HMM alignment. Nat Methods 9, 173-175 (2012). doi>

Table T1:

Primary amino acid sequence for which templates were searched and models were built.

```
KAYTWPINKQGGSAPGQNKFGVDLGKQQDADTAAWYSPSMYNIVRQNGEDVHIVIKPDKECVVNSGLGSVRGARMAEMSYSRARSTQLQRLTDPMVWRYG
QMQPTSWDDALDLVARVTCAVINDQGEDGLFVSAFDHGGAGGGYENTWGTGKLYFEAMKVKNIRIHNRPAYNSEVHATRDMGVGELNNCYEDAELADTIV
AVGTNALETQTNYSLNHWVPNLRGTSAQKKRAEFGAEETPPARIIIVDPRRTVTVNACEVEAGKDRVMHLAINSGTDLALFNAWFTHINERGWTDKAFIG
ASTNGLDKALAANKTSLDEAARITGLTVDQIRQSAEWIAQPKAANARRRTMFCY
```

Table T2:

Template	Seq Identity	Oligo-state	QSQE	Found by	Method	Resolution	Seq Similarity	Coverage	Description
5nqd.1.A	73.94	homo-dimer	0.69	BLAST	X-ray	2.20Å	0.53	1.00	AroA
4aay.1.A	73.94	homo-dimer	0.67	BLAST	X-ray	2.70Å	0.53	1.00	AROA
5nqd.1.A	73.30	homo-dimer	0.66	HHblits	X-ray	2.20Å	0.52	0.99	AroA
4aay.1.A	73.50	homo-dimer	0.66	HHblits	X-ray	2.70Å	0.52	0.99	AROA
1g8k.1.A	51.14	monomer	-	BLAST	X-ray	1.64Å	0.45	0.99	ARSENITE OXIDASE
1g8j.1.A	51.14	monomer	-	BLAST	X-ray	2.03Å	0.45	0.99	ARSENITE OXIDASE
1g8k.1.A	49.29	monomer	-	HHblits	X-ray	1.64Å	0.44	0.99	ARSENITE OXIDASE
1g8j.1.A	49.43	monomer	-	HHblits	X-ray	2.03Å	0.44	0.99	ARSENITE OXIDASE
1q16.1.A	19.30	monomer	-	HHblits	X-ray	1.90Å	0.29	0.64	Respiratory nitrate reductase 1 alpha chain
3ir5.1.A	19.30	monomer	-	HHblits	X-ray	2.30Å	0.29	0.64	Respiratory nitrate reductase 1 alpha chain
3egw.1.A	19.30	monomer	-	HHblits	X-ray	1.90Å	0.29	0.64	Respiratory nitrate reductase 1 alpha chain

Template	Seq Identity	Oligo-state	QSQE	Found by	Method	Resolution	Seq Similarity	Coverage	Description
3ir7.1.A	18.86	monomer	-	HHblits	X-ray	2.50Å	0.29	0.64	Respiratory nitrate reductase 1 alpha chain
1y4z.1.A	19.30	monomer	-	HHblits	X-ray	2.00Å	0.29	0.64	Respiratory nitrate reductase 1 alpha chain
3ir6.1.A	19.30	monomer	-	HHblits	X-ray	2.80Å	0.29	0.64	Respiratory nitrate reductase 1 alpha chain
5o31.1.8	18.91	monomer	-	HHblits	EM	4.13Å	0.28	0.57	NADH-ubiquinone oxidoreductase 75 kDa subunit, mitochondrial
6qcf.1.C	19.40	monomer	-	HHblits	EM	NA	0.28	0.57	NADH:ubiquinone oxidoreductase core subunit S1
6qc5.1.C	19.40	monomer	-	HHblits	EM	NA	0.28	0.57	NADH:ubiquinone oxidoreductase core subunit S1
5lnk.1.C	19.40	monomer	-	HHblits	EM	3.90Å	0.28	0.57	Mitochondrial complex I, 75 kDa subunit
5gpn.24.A	19.40	monomer	-	HHblits	EM	NA	0.28	0.57	NADH-ubiquinone oxidoreductase 75 kDa subunit
2ev3.1.B	14.71	monomer	-	HHblits	X-ray	2.68Å	0.27	0.19	Hypothetical protein Rv1264/MT1302
2ev2.1.A	14.71	monomer	-	HHblits	X-ray	2.35Å	0.27	0.19	Hypothetical protein Rv1264/MT1302
2ev4.1.B	14.71	monomer	-	HHblits	X-ray	2.28Å	0.27	0.19	Hypothetical protein Rv1264/MT1302
2ev3.1.A	14.71	monomer	-	HHblits	X-ray	2.68Å	0.27	0.19	Hypothetical protein Rv1264/MT1302
2ev4.1.A	14.71	monomer	-	HHblits	X-ray	2.28Å	0.27	0.19	Hypothetical protein Rv1264/MT1302
2ev1.1.A	14.71	monomer	-	HHblits	X-ray	1.60Å	0.27	0.19	Hypothetical protein Rv1264/MT1302
4twi.1.A	25.00	monomer	-	HHblits	X-ray	1.79Å	0.32	0.14	NAD-dependent protein deacylase 1
1ici.1.A	26.09	monomer	-	HHblits	X-ray	2.10Å	0.32	0.13	TRANSCRIPTIONAL REGULATORY PROTEIN, SIR2 FAMILY
1m2g.1.A	25.00	monomer	-	HHblits	X-ray	1.70Å	0.32	0.14	Silent Information Regulator 2
1m2n.1.A	26.67	monomer	-	HHblits	X-ray	2.60Å	0.33	0.13	Silent Information Regulator 2
1m2n.1.B	26.67	monomer	-	HHblits	X-ray	2.60Å	0.33	0.13	Silent Information Regulator 2
1m2k.1.A	26.09	monomer	-	HHblits	X-ray	1.47Å	0.32	0.13	Silent Information Regulator 2
1m2h.1.A	26.09	monomer	-	HHblits	X-ray	1.80Å	0.32	0.13	Silent Information Regulator 2
1m2j.1.A	22.92	monomer	-	HHblits	X-ray	1.70Å	0.31	0.14	Silent Information Regulator 2
3egw.1.A	15.22	monomer	-	HHblits	X-ray	1.90Å	0.30	0.13	Respiratory nitrate reductase 1 alpha chain
3ir5.1.A	13.04	monomer	-	HHblits	X-ray	2.30Å	0.28	0.13	Respiratory nitrate reductase 1 alpha chain
3ir7.1.A	13.04	monomer	-	HHblits	X-ray	2.50Å	0.28	0.13	Respiratory nitrate reductase 1 alpha chain
3ir6.1.A	13.04	monomer	-	HHblits	X-ray	2.80Å	0.29	0.13	Respiratory nitrate reductase 1 alpha chain
1y4z.1.A	15.22	monomer	-	HHblits	X-ray	2.00Å	0.30	0.13	Respiratory nitrate reductase 1 alpha chain
1q16.1.A	13.04	monomer	-	HHblits	X-ray	1.90Å	0.28	0.13	Respiratory nitrate reductase 1 alpha chain

The table above shows the top 39 filtered templates. A further 75 templates were found which were considered to be less suitable for modelling than the filtered list.
3trj.1.A, 4aay.1.A, 1wcl.1.A, 6g2j.1.G, 6rfr.1.A, 5t5i.1.B, 1ogy.1.A, 6amk.1.A, 5tma.1.A, 3fkj.1.A, 2fug.2.C, 5gpn.24.A, 2v45.1.A, 2v3v.1.A, 6cz7.1.A, 3m9s.1.C, 5xtb.1.L, 2vpx.1.D, 6s6y.1.B, 6b9s.1.B, 1u9l.1.A, 3oe1.1.A, 6qcf.1.C, 6tg9.1.A, 5by2.1.A, 1tmo.1.A, 5e7o.1.A, 1aa6.1.A, 6btm.1.B, 4zp1.1.A, 6qc5.1.C, 3o5a.1.A, 1dmr.1.A, 6f0k.1.B, 6amk.1.B, 2iv2.1.A, 5lnk.1.C, 2nya.1.A, 3dt5.1.A, 1u9l.2.A, 1dms.1.A, 2xbl.1.A, 4ydd.1.A, 6sdv.1.A, 2vpz.1.A, 1kqf.1.A, 4u6n.1.A, 4v4c.1.A, 5nqd.1.A, 6b9s.1.A, 6b9t.1.A, 1g8j.1.A, 5o31.1.8, 4dmr.1.A, 6sdr.1.A, 2ivf.1.A, 5lu7.1.A, 2e7z.1.A, 1g8k.1.A, 3iwf.1.A, 1e60.1.A, 2o3f.1.A, 2wva.1.A, 4k9k.1.A, 1eu1.1.A, 5ahk.1.A, 6gcs.1.A, 1h0h.1.A, 1fdo.1.A, 6ib8.1.C, 1wcn.1.A, 1y6u.1.A, 1e18.1.A, 2jzb.1.B, 6rfq.1.A

SWISS-MODEL Homology Modelling Report

Model Building Report

This document lists the results for the homology modelling project "Untitled Project" submitted to SWISS-MODEL workspace on May 28, 2020, 1:35 p.m..The submitted primary amino acid sequence is given in Table T1.

If you use any results in your research, please cite the relevant publications:

- Waterhouse, A., Bertoni, M., Bienert, S., Studer, G., Tauriello, G., Gumienny, R., Heer, F.T., de Beer, T.A.P., Rempfer, C., Bordoli, L., Lepore, R., Schwede, T. SWISS-MODEL: homology modelling of protein structures and complexes. Nucleic Acids Res. 46(W1), W296-W303 (2018). doi>
- Guex, N., Peitsch, M.C., Schwede, T. Automated comparative protein structure modeling with SWISS-MODEL and Swiss-PdbViewer: A historical perspective. Electrophoresis 30, S162-S173 (2009). doi>
- Bienert, S., Waterhouse, A., de Beer, T.A.P., Tauriello, G., Studer, G., Bordoli, L., Schwede, T. The SWISS-MODEL Repository - new features and functionality. Nucleic Acids Res. 45, D313-D319 (2017). doi>
- Studer, G., Rempfer, C., Waterhouse, A.M., Gumienny, G., Haas, J., Schwede, T. QMEANDisCo - distance constraints applied on model quality estimation. Bioinformatics 36, 1765-1771 (2020). doi>
- Bertoni, M., Kiefer, F., Biasini, M., Bordoli, L., Schwede, T. Modeling protein quaternary structure of homo- and hetero-oligomers beyond binary interactions by homology. Scientific Reports 7 (2017). doi>

Results

The SWISS-MODEL template library (SMTL version 2020-05-27, PDB release 2020-05-22) was searched with BLAST (Camacho et al.) and HHBlits (Remmert et al.) for evolutionary related structures matching the target sequence in Table T1. For details on the template search, see Materials and Methods. Overall 162 templates were found (Table T2).

Models

The following model was built (see Materials and Methods "Model Building"):

Model #01	File	Built with	Oligo-State	Ligands	GMQE	QMEAN
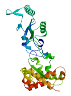	PDB	ProMod3 3.0.0	monomer (matching prediction)	None	0.91	-0.91

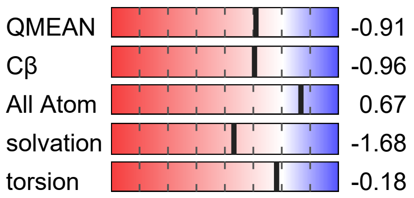

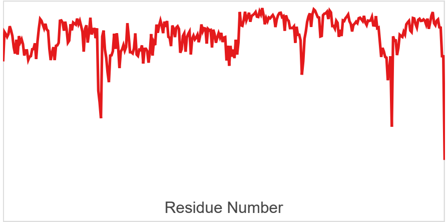

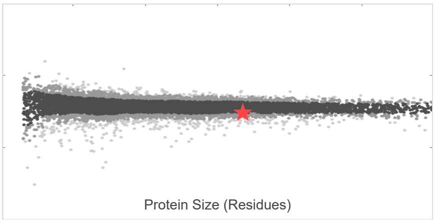

Template	Seq Identity	Oligo-state	QSQE	Found by	Method	Resolution	Seq Similarity	Range	Coverage	Description
5nqd.1.A	75.22	homo-dimer	0.60	BLAST	X-ray	2.20Å	0.53	1 - 335	0.99	AroA

Excluded ligands

Ligand Name.Number	Reason for Exclusion	Description
4MO.4	Binding site not conserved.	MOLYBDENUM(IV) ION
4MO.17	Binding site not conserved.	MOLYBDENUM(IV) ION
EDO.13	Not biologically relevant.	1,2-ETHANEDIOL
F3S.5	Binding site not conserved.	FE3-S4 CLUSTER
F3S.18	Binding site not conserved.	FE3-S4 CLUSTER
FES.12	Binding site not conserved.	FE2/S2 (INORGANIC) CLUSTER

Ligand Name.Number	Reason for Exclusion	Description
FES.22	Binding site not conserved.	FE2/S2 (INORGANIC) CLUSTER
GOL.11	Not biologically relevant.	GLYCEROL
GOL.21	Not biologically relevant.	GLYCEROL
MGD.1	Binding site not conserved.	2-AMINO-5,6-DIMERCAPTO-7-METHYL-3,7,8A,9-TETRAHYDRO-8-OXA-1,3,9,10-TETRAAZA-ANTHRACEN-4-ONE GUANOSINE DINUCLEOTIDE
MGD.2	Binding site not conserved.	2-AMINO-5,6-DIMERCAPTO-7-METHYL-3,7,8A,9-TETRAHYDRO-8-OXA-1,3,9,10-TETRAAZA-ANTHRACEN-4-ONE GUANOSINE DINUCLEOTIDE
MGD.14	Binding site not conserved.	2-AMINO-5,6-DIMERCAPTO-7-METHYL-3,7,8A,9-TETRAHYDRO-8-OXA-1,3,9,10-TETRAAZA-ANTHRACEN-4-ONE GUANOSINE DINUCLEOTIDE
MGD.15	Binding site not conserved.	2-AMINO-5,6-DIMERCAPTO-7-METHYL-3,7,8A,9-TETRAHYDRO-8-OXA-1,3,9,10-TETRAAZA-ANTHRACEN-4-ONE GUANOSINE DINUCLEOTIDE
O.3	Binding site not conserved.	OXYGEN ATOM
O.16	Binding site not conserved.	OXYGEN ATOM
PGE.10	Not biologically relevant.	TRIETHYLENE GLYCOL
SO4.6	Not biologically relevant.	SULFATE ION
SO4.7	Not biologically relevant.	SULFATE ION
SO4.8	Not biologically relevant.	SULFATE ION
SO4.9	Not biologically relevant.	SULFATE ION
SO4.19	Not biologically relevant.	SULFATE ION
SO4.20	Not biologically relevant.	SULFATE ION

```
Target    QGGTDPSQNKFKVDLAKQQGAESDAWYSPSMYNIVKQDGKDVHVVIMPDKNCVVNSGLGSVRGARMAETSYSEARSTQQQ
5nqd.1.A  QGGTDPQNNIFGVDLSEQQQAESDAWYSPSMYNVVKQDGRDVHVVIKPDHECVVNSGLGSVRGARMAETSFSEARNTQQQ

Target    RLTHPMVWRYGAMSPTSWDDALDLVARVTCQIVKDQGEDGLFVSAFDHGGAGGGYENTWGTGKLYFGAMKVKNIRIHNRP
5nqd.1.A  RLTDPLVWRYGQMQPTSWDDALDLVARVTAKIVKEKGEDALIVSAFDHGGAGGGYENTWGTGKLYFEAMKVKNIRIHNRP

Target    AYNSEVHATRDMGIGELNNCYEDAELADTIVVVGANPLETQTNYFLNHWVPNLRGTSMDKKRAELPNEAHPPARIVIIDP
5nqd.1.A  AYNSEVHGTRDMGVGELNNCYEDAELADTIVAVGTNALETQTNYFLNHWIPNLRGESLGKKKELMPEEPHEAGRIIIVDP

Target    RRTVTVNACEVEAGKDRVMHLAINSGSDLALFNAWMTYIAEKGWVDKALIAAST------------------------NG
5nqd.1.A  RRTVTVNACEQTAGADNVLHLAINSGTDLALFNALFTYIADKGWVDRDFIDKSTLREGTARPPLYPARGVSEANPGHLSS

Target    FDKMVAANKTTLEQAAALTGLTVDQIRQSAEWIASPKDGNA
5nqd.1.A  FEDAVEGCRMSIEEAAEITGLDAAQIIKAAEWIGMPKEG--
```

Materials and Methods

Template Search

Template search with BLAST and HHBlits has been performed against the SWISS-MODEL template library (SMTL, last update: 2020-05-27, last included PDB release: 2020-05-22).

The target sequence was searched with BLAST against the primary amino acid sequence contained in the SMTL. A total of 14 templates were found.

An initial HHblits profile has been built using the procedure outlined in (Remmert et al.), followed by 1 iteration of HHblits against NR20. The obtained profile has then be searched against all profiles of the SMTL. A total of 148 templates were

found.

Model Building

Models are built based on the target-template alignment using ProMod3. Coordinates which are conserved between the target and the template are copied from the template to the model. Insertions and deletions are remodelled using a fragment library. Side chains are then rebuilt. Finally, the geometry of the resulting model is regularized by using a force field. In case loop modelling with ProMod3 fails, an alternative model is built with PROMOD-II (Guex et al.).

Model Quality Estimation

The global and per-residue model quality has been assessed using the QMEAN scoring function (Studer et al.).

Ligand Modelling

Ligands present in the template structure are transferred by homology to the model when the following criteria are met: (a) The ligands are annotated as biologically relevant in the template library, (b) the ligand is in contact with the model, (c) the ligand is not clashing with the protein, (d) the residues in contact with the ligand are conserved between the target and the template. If any of these four criteria is not satisfied, a certain ligand will not be included in the model. The model summary includes information on why and which ligand has not been included.

Oligomeric State Conservation

The quaternary structure annotation of the template is used to model the target sequence in its oligomeric form. The method (Bertoni et al.) is based on a supervised machine learning algorithm, Support Vector Machines (SVM), which combines interface conservation, structural clustering, and other template features to provide a quaternary structure quality estimate (QSQE). The QSQE score is a number between 0 and 1, reflecting the expected accuracy of the interchain contacts for a model built based a given alignment and template. Higher numbers indicate higher reliability. This complements the GMQE score which estimates the accuracy of the tertiary structure of the resulting model.

References

- **BLAST**
  Camacho, C., Coulouris, G., Avagyan, V., Ma, N., Papadopoulos, J., Bealer, K., Madden, T.L. BLAST+: architecture and applications. BMC Bioinformatics 10, 421-430 (2009). doi>
- **HHblits**
  Remmert, M., Biegert, A., Hauser, A., Söding, J. HHblits: lightning-fast iterative protein sequence searching by HMM-HMM alignment. Nat Methods 9, 173-175 (2012). doi>

Table T1:

Primary amino acid sequence for which templates were searched and models were built.

```
QGGTDPSQNKFKVDLAKQQGAESDAWYSPSMYNIVKQDGKDVHVVIMPDKNCVVNSGLGSVRGARMAETSYSEARSTQQQRLTHPMVWRYGAMSPTSWDD
ALDLVARVTCQIVKDQGEDGLFVSAFDHGGAGGGYENTWGTGKLYFGAMKVKNIRIHNRPAYNSEVHATRDMGIGELNNCYEDAELADTIVVVGANPLET
QTNYFLNHWVPNLRGTSMDKKRAELPNEAHPPARIVIIDPRRTVTVNACEVEAGKDRVMHLAINSGSDLALFNAWMTYIAEKGWVDKALIAASTNGFDKM
VAANKTTLEQAAALTGLTVDQIRQSAEWIASPKDGNA
```

Table T2:

Template	Seq Identity	Oligo-state	QSQE	Found by	Method	Resolution	Seq Similarity	Coverage	Description
5nqd.1.A	75.22	homo-dimer	0.60	BLAST	X-ray	2.20Å	0.53	0.99	AroA
4aay.1.A	75.22	homo-dimer	0.61	BLAST	X-ray	2.70Å	0.53	0.99	AROA
5nqd.1.A	74.47	homo-dimer	0.58	HHblits	X-ray	2.20Å	0.53	0.99	AroA
4aay.1.A	74.47	homo-dimer	0.59	HHblits	X-ray	2.70Å	0.53	0.99	AROA
1g8k.1.A	49.85	monomer	-	BLAST	X-ray	1.64Å	0.45	0.98	ARSENITE OXIDASE
1g8j.1.A	49.85	monomer	-	BLAST	X-ray	2.03Å	0.45	0.98	ARSENITE OXIDASE
1g8k.1.A	48.35	monomer	-	HHblits	X-ray	1.64Å	0.44	0.99	ARSENITE OXIDASE
1g8j.1.A	48.35	monomer	-	HHblits	X-ray	2.03Å	0.44	0.99	ARSENITE OXIDASE
1aa6.1.A	28.04	monomer	-	HHblits	X-ray	2.30Å	0.34	0.80	FORMATE DEHYDROGENASE H
1fdo.1.A	28.04	monomer	-	HHblits	X-ray	2.80Å	0.34	0.80	FORMATE DEHYDROGENASE H
2iv2.1.A	28.04	monomer	-	HHblits	X-ray	2.27Å	0.34	0.80	Formate dehydrogenase H
4dmr.1.A	23.72	monomer	-	HHblits	X-ray	1.90Å	0.32	0.81	DMSO REDUCTASE

Template	Seq Identity	Oligo-state	QSQE	Found by	Method	Resolution	Seq Similarity	Coverage	Description
1dmr.1.A	23.36	monomer	-	HHblits	X-ray	1.82Å	0.31	0.81	DMSO REDUCTASE
1e18.1.A	23.72	monomer	-	HHblits	X-ray	2.00Å	0.32	0.81	DMSO REDUCTASE.
1e60.1.A	23.36	monomer	-	HHblits	X-ray	2.00Å	0.31	0.81	Dimethyl sulfoxide/trimethylamine N-oxide reductase
1dms.1.A	22.63	monomer	-	HHblits	X-ray	1.88Å	0.31	0.81	DMSO REDUCTASE
1aa6.1.A	30.67	monomer	-	BLAST	X-ray	2.30Å	0.36	0.67	FORMATE DEHYDROGENASE H
1fdo.1.A	30.67	monomer	-	BLAST	X-ray	2.80Å	0.36	0.67	FORMATE DEHYDROGENASE H
2iv2.1.A	30.67	monomer	-	BLAST	X-ray	2.27Å	0.36	0.67	Formate dehydrogenase H
1q16.1.A	17.98	monomer	-	HHblits	X-ray	1.90Å	0.29	0.68	Respiratory nitrate reductase 1 alpha chain
3egw.1.A	17.67	monomer	-	HHblits	X-ray	1.90Å	0.28	0.69	Respiratory nitrate reductase 1 alpha chain
3ir7.1.A	17.75	monomer	-	HHblits	X-ray	2.50Å	0.28	0.69	Respiratory nitrate reductase 1 alpha chain
1y4z.1.A	17.98	monomer	-	HHblits	X-ray	2.00Å	0.29	0.68	Respiratory nitrate reductase 1 alpha chain
3ir5.1.A	17.98	monomer	-	HHblits	X-ray	2.30Å	0.29	0.68	Respiratory nitrate reductase 1 alpha chain
3ir6.1.A	17.98	monomer	-	HHblits	X-ray	2.80Å	0.29	0.68	Respiratory nitrate reductase 1 alpha chain
2ev4.1.B	10.29	monomer	-	HHblits	X-ray	2.28Å	0.27	0.20	Hypothetical protein Rv1264/MT1302
2ev2.1.A	10.29	monomer	-	HHblits	X-ray	2.35Å	0.27	0.20	Hypothetical protein Rv1264/MT1302
2ev4.1.A	10.29	monomer	-	HHblits	X-ray	2.28Å	0.27	0.20	Hypothetical protein Rv1264/MT1302
2ev3.1.A	10.29	monomer	-	HHblits	X-ray	2.68Å	0.27	0.20	Hypothetical protein Rv1264/MT1302
2ev3.1.B	10.29	monomer	-	HHblits	X-ray	2.68Å	0.27	0.20	Hypothetical protein Rv1264/MT1302
2ev1.1.A	10.29	monomer	-	HHblits	X-ray	1.60Å	0.27	0.20	Hypothetical protein Rv1264/MT1302
4twi.1.A	22.92	monomer	-	HHblits	X-ray	1.79Å	0.31	0.14	NAD-dependent protein deacylase 1
1ici.1.A	22.92	monomer	-	HHblits	X-ray	2.10Å	0.31	0.14	TRANSCRIPTIONAL REGULATORY PROTEIN, SIR2 FAMILY
3ir7.1.A	17.39	monomer	-	HHblits	X-ray	2.50Å	0.29	0.14	Respiratory nitrate reductase 1 alpha chain
3egw.1.A	17.39	monomer	-	HHblits	X-ray	1.90Å	0.29	0.14	Respiratory nitrate reductase 1 alpha chain
3ir6.1.A	17.39	monomer	-	HHblits	X-ray	2.80Å	0.29	0.14	Respiratory nitrate reductase 1 alpha chain
3ir5.1.A	17.39	monomer	-	HHblits	X-ray	2.30Å	0.29	0.14	Respiratory nitrate reductase 1 alpha chain
1m2g.1.A	25.00	monomer	-	HHblits	X-ray	1.70Å	0.32	0.14	Silent Information Regulator 2
1m2k.1.A	25.00	monomer	-	HHblits	X-ray	1.47Å	0.32	0.14	Silent Information Regulator 2
1m2h.1.A	26.09	monomer	-	HHblits	X-ray	1.80Å	0.32	0.14	Silent Information Regulator 2
1m2n.1.A	24.44	monomer	-	HHblits	X-ray	2.60Å	0.31	0.13	Silent Information Regulator 2
1m2n.1.B	24.44	monomer	-	HHblits	X-ray	2.60Å	0.31	0.13	Silent Information Regulator 2
1m2j.1.A	22.92	monomer	-	HHblits	X-ray	1.70Å	0.31	0.14	Silent Information Regulator 2
1q16.1.A	17.39	monomer	-	HHblits	X-ray	1.90Å	0.29	0.14	Respiratory nitrate reductase 1 alpha chain
1y4z.1.A	17.39	monomer	-	HHblits	X-ray	2.00Å	0.29	0.14	Respiratory nitrate reductase 1 alpha chain

The table above shows the top 45 filtered templates. A further 74 templates were found which were considered to be less suitable for modelling than the filtered list.
4aay.1.A, 1wcl.1.A, 6g2j.1.G, 6rfr.1.A, 5t5i.1.B, 1ogy.1.A, 6amk.1.A, 5tma.1.A, 3fkj.1.A, 2fug.2.C, 5gpn.24.A, 2v45.1.A,

2v3v.1.A, 6cz7.1.A, 3m9s.1.C, 5xtb.1.L, 2vpx.1.D, 6s6y.1.B, 6b9s.1.B, 1u9l.1.A, 6qcf.1.C, 6tg9.1.A, 5by2.1.A, 1tmo.1.A, 5e7o.1.A, 6btm.1.B, 1aa6.1.A, 4zp1.1.A, 6qc5.1.C, 3o5a.1.A, 1dmr.1.A, 6f0k.1.B, 6amk.1.B, 2iv2.1.A, 5lnk.1.C, 2nya.1.A, 1b22.1.A, 3dt5.1.A, 1u9l.2.A, 1dms.1.A, 2xbl.1.A, 4ydd.1.A, 6sdv.1.A, 2vpz.1.A, 1kqf.1.A, 3g68.1.A, 4rjj.1.A, 4v4c.1.A, 5nqd.1.A, 6b9s.1.A, 6b9t.1.A, 1g8j.1.A, 5o31.1.8, 2ivf.1.A, 6sdr.1.A, 4dmr.1.A, 5lu7.1.A, 2e7z.1.A, 3knz.1.A, 4cok.1.A, 1g8k.1.A, 3iwf.1.A, 1e60.1.A, 5npu.1.A, 1eu1.1.A, 6gcs.1.A, 1h0h.1.A, 1fdo.1.A, 6ib8.1.C, 1wcn.1.A, 1y6u.1.A, 1e18.1.A, 2jzb.1.B, 6rfq.1.A

SWISS-MODEL Homology Modelling Report

Model Building Report

This document lists the results for the homology modelling project "Untitled Project" submitted to SWISS-MODEL workspace on May 28, 2020, 1:42 p.m..The submitted primary amino acid sequence is given in Table T1.

If you use any results in your research, please cite the relevant publications:

- Waterhouse, A., Bertoni, M., Bienert, S., Studer, G., Tauriello, G., Gumienny, R., Heer, F.T., de Beer, T.A.P., Rempfer, C., Bordoli, L., Lepore, R., Schwede, T. SWISS-MODEL: homology modelling of protein structures and complexes. Nucleic Acids Res. 46(W1), W296-W303 (2018). doi>
- Guex, N., Peitsch, M.C., Schwede, T. Automated comparative protein structure modeling with SWISS-MODEL and Swiss-PdbViewer: A historical perspective. Electrophoresis 30, S162-S173 (2009). doi>
- Bienert, S., Waterhouse, A., de Beer, T.A.P., Tauriello, G., Studer, G., Bordoli, L., Schwede, T. The SWISS-MODEL Repository - new features and functionality. Nucleic Acids Res. 45, D313-D319 (2017). doi>
- Studer, G., Rempfer, C., Waterhouse, A.M., Gumienny, G., Haas, J., Schwede, T. QMEANDisCo - distance constraints applied on model quality estimation. Bioinformatics 36, 1765-1771 (2020). doi>
- Bertoni, M., Kiefer, F., Biasini, M., Bordoli, L., Schwede, T. Modeling protein quaternary structure of homo- and hetero-oligomers beyond binary interactions by homology. Scientific Reports 7 (2017). doi>

Results

The SWISS-MODEL template library (SMTL version 2020-05-27, PDB release 2020-05-22) was searched with BLAST (Camacho et al.) and HHBlits (Remmert et al.) for evolutionary related structures matching the target sequence in Table T1. For details on the template search, see Materials and Methods. Overall 185 templates were found (Table T2).

Models

The following model was built (see Materials and Methods "Model Building"):

Model #01	File	Built with	Oligo-State	Ligands	GMQE	QMEAN
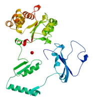	PDB	ProMod3 3.0.0	monomer (matching prediction)	1 x O: OXYGEN ATOM;	0.91	-1.53

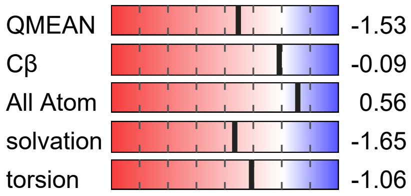

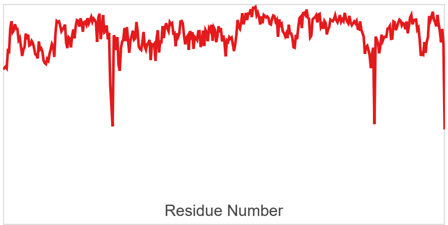

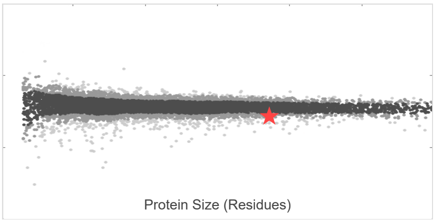

Template	Seq Identity	Oligo-state	QSQE	Found by	Method	Resolution	Seq Similarity	Range	Coverage	Description
5nqd.1.A	75.81	homo-dimer	0.62	BLAST	X-ray	2.20Å	0.54	1 - 372	1.00	AroA

Included Ligands

Ligand	Description
1 x O	OXYGEN ATOM

Excluded ligands

Ligand Name.Number	Reason for Exclusion	Description
4MO.4	Binding site not conserved.	MOLYBDENUM(IV) ION
4MO.17	Binding site not conserved.	MOLYBDENUM(IV) ION
EDO.13	Not biologically relevant.	1,2-ETHANEDIOL
F3S.5	Binding site not conserved.	FE3-S4 CLUSTER

Ligand Name.Number	Reason for Exclusion	Description
F3S.18	Binding site not conserved.	FE3-S4 CLUSTER
FES.12	Binding site not conserved.	FE2/S2 (INORGANIC) CLUSTER
FES.22	Binding site not conserved.	FE2/S2 (INORGANIC) CLUSTER
GOL.11	Not biologically relevant.	GLYCEROL
GOL.21	Not biologically relevant.	GLYCEROL
MGD.1	Binding site not conserved.	2-AMINO-5,6-DIMERCAPTO-7-METHYL-3,7,8A,9-TETRAHYDRO-8-OXA-1,3,9,10-TETRAAZA-ANTHRACEN-4-ONE GUANOSINE DINUCLEOTIDE
MGD.2	Binding site not conserved.	2-AMINO-5,6-DIMERCAPTO-7-METHYL-3,7,8A,9-TETRAHYDRO-8-OXA-1,3,9,10-TETRAAZA-ANTHRACEN-4-ONE GUANOSINE DINUCLEOTIDE
MGD.14	Binding site not conserved.	2-AMINO-5,6-DIMERCAPTO-7-METHYL-3,7,8A,9-TETRAHYDRO-8-OXA-1,3,9,10-TETRAAZA-ANTHRACEN-4-ONE GUANOSINE DINUCLEOTIDE
MGD.15	Binding site not conserved.	2-AMINO-5,6-DIMERCAPTO-7-METHYL-3,7,8A,9-TETRAHYDRO-8-OXA-1,3,9,10-TETRAAZA-ANTHRACEN-4-ONE GUANOSINE DINUCLEOTIDE
O.16	Binding site not conserved.	OXYGEN ATOM
PGE.10	Not biologically relevant.	TRIETHYLENE GLYCOL
SO4.6	Not biologically relevant.	SULFATE ION
SO4.7	Not biologically relevant.	SULFATE ION
SO4.8	Not biologically relevant.	SULFATE ION
SO4.9	Not biologically relevant.	SULFATE ION
SO4.19	Not biologically relevant.	SULFATE ION
SO4.20	Not biologically relevant.	SULFATE ION

```
Target    HFCIVGCGYKAYTWDINKQGGADPSQNKFKADLSKQDGANSDAWYSPSMHNIVKQGGKDVHLVIMPDKGCSVNSGLGSVR
5nqd.1.A  HFCIVGCGYHAYTWPINKQGGTDPQNNIFGVDLSEQQQAESDAWYSPSMYNVVKQDGRDVHVVIKPDHECVVNSGLGSVR

Target    GARMAETSFSEARSTQAQRLTDPMVWRYGAMSPTSWDDALDLVARVTCQVVKDQGEDGLFVSAFDHGGAGGGYENTWGTG
5nqd.1.A  GARMAETSFSEARNTQQQRLTDPLVWRYGQMQPTSWDDALDLVARVTAKIVKEKGEDALIVSAFDHGGAGGGYENTWGTG

Target    KLYFGAMKVRNIRIHNRPAYNSEVHATRDMGVGELNNCYEDAQLADTIVMVGANSLETQTNYFLNHWVPNLRGTSIDKKK
5nqd.1.A  KLYFEAMKVKNIRIHNRPAYNSEVHGTRDMGVGELNNCYEDAELADTIVAVGTNALETQTNYFLNHWIPNLRGESLGKKK

Target    AELPNEPHAAGRIIIVDPRRTVTVNACETEAGKDNVMHLAINSGTDLALFNAWMTYIAEKGWTDKALIAAST--------
5nqd.1.A  ELMPEEPHEAGRIIIVDPRRTVTVNACEQTAGADNVLHLAINSGTDLALFNALFTYIADKGWVDRDFIDKSTLREGTARP

Target    ----------------NGFDKMAAANKTTLQQAATLTGLTVDQIRQSAEWIAMPKEGNARRRTMFAYEKGLIWGND
5nqd.1.A  PLYPARGVSEANPGHLSSFEDAVEGCRMSIEEAAEITGLDAAQIIKAAEWIGMPKEGGKRRRVMFGYEKGLIWGND
```

Materials and Methods

Template Search

Template search with BLAST and HHBlits has been performed against the SWISS-MODEL template library (SMTL, last update: 2020-05-27, last included PDB release: 2020-05-22).

The target sequence was searched with BLAST against the primary amino acid sequence contained in the SMTL. A total of 12 templates were found.

An initial HHblits profile has been built using the procedure outlined in (Remmert et al.), followed by 1 iteration of HHblits against NR20. The obtained profile has then be searched against all profiles of the SMTL. A total of 173 templates were found.

Model Building

Models are built based on the target-template alignment using ProMod3. Coordinates which are conserved between the target and the template are copied from the template to the model. Insertions and deletions are remodelled using a fragment library. Side chains are then rebuilt. Finally, the geometry of the resulting model is regularized by using a force field. In case loop modelling with ProMod3 fails, an alternative model is built with PROMOD-II (Guex et al.).

Model Quality Estimation

The global and per-residue model quality has been assessed using the QMEAN scoring function (Studer et al.).

Ligand Modelling

Ligands present in the template structure are transferred by homology to the model when the following criteria are met: (a) The ligands are annotated as biologically relevant in the template library, (b) the ligand is in contact with the model, (c) the ligand is not clashing with the protein, (d) the residues in contact with the ligand are conserved between the target and the template. If any of these four criteria is not satisfied, a certain ligand will not be included in the model. The model summary includes information on why and which ligand has not been included.

Oligomeric State Conservation

The quaternary structure annotation of the template is used to model the target sequence in its oligomeric form. The method (Bertoni et al.) is based on a supervised machine learning algorithm, Support Vector Machines (SVM), which combines interface conservation, structural clustering, and other template features to provide a quaternary structure quality estimate (QSQE). The QSQE score is a number between 0 and 1, reflecting the expected accuracy of the interchain contacts for a model built based a given alignment and template. Higher numbers indicate higher reliability. This complements the GMQE score which estimates the accuracy of the tertiary structure of the resulting model.

References

- **BLAST**
  Camacho, C., Coulouris, G., Avagyan, V., Ma, N., Papadopoulos, J., Bealer, K., Madden, T.L. BLAST+: architecture and applications. BMC Bioinformatics 10, 421-430 (2009). doi>
- **HHblits**
  Remmert, M., Biegert, A., Hauser, A., Söding, J. HHblits: lightning-fast iterative protein sequence searching by HMM-HMM alignment. Nat Methods 9, 173-175 (2012). doi>

Table T1:

Primary amino acid sequence for which templates were searched and models were built.

```
HFCIVGCGYKAYTWDINKQGGADPSQNKFKADLSKQDGANSDAWYSPSMHNIVKQGGKDVHLVIMPDKGCSVNSGLGSVRGARMAETSFSEARSTQAQRL
TDPMVWRYGAMSPTSWDDALDLVARVTCQVVKDQGEDGLFVSAFDHGGAGGGYENTWGTGKLYFGAMKVRNIRIHNRPAYNSEVHATRDMGVGELNNCYE
DAQLADTIVMVGANSLETQTNYFLNHWVPNLRGTSIDKKKAELPNEPHAAGRIIIVDPRRTVTVNACETEAGKDNVMHLAINSGTDLALFNAWMTYIAEK
GWTDKALIAASTNGFDKMAAANKTTLQQAATLTGLTVDQIRQSAEWIAMPKEGNARRRTMFAYEKGLIWGND
```

Table T2:

Template	Seq Identity	Oligo-state	QSQE	Found by	Method	Resolution	Seq Similarity	Coverage	Description
5nqd.1.A	75.81	homo-dimer	0.62	BLAST	X-ray	2.20Å	0.54	1.00	AroA
4aay.1.A	75.81	homo-dimer	0.61	BLAST	X-ray	2.70Å	0.54	1.00	AROA
5nqd.1.A	75.14	homo-dimer	0.60	HHblits	X-ray	2.20Å	0.54	0.99	AroA
4aay.1.A	75.14	homo-dimer	0.60	HHblits	X-ray	2.70Å	0.54	0.99	AROA
1g8k.1.A	50.00	monomer	-	BLAST	X-ray	1.64Å	0.45	0.99	ARSENITE OXIDASE
1g8j.1.A	50.00	monomer	-	BLAST	X-ray	2.03Å	0.45	0.99	ARSENITE OXIDASE
1g8k.1.A	48.65	monomer	-	HHblits	X-ray	1.64Å	0.44	0.99	ARSENITE OXIDASE
1g8j.1.A	48.65	monomer	-	HHblits	X-ray	2.03Å	0.44	0.99	ARSENITE OXIDASE
1dmr.1.A	23.43	monomer	-	HHblits	X-ray	1.82Å	0.31	0.77	DMSO REDUCTASE
4dmr.1.A	23.43	monomer	-	HHblits	X-ray	1.90Å	0.31	0.77	DMSO REDUCTASE
1e60.1.A	23.08	monomer	-	HHblits	X-ray	2.00Å	0.31	0.77	Dimethyl sulfoxide/trimethylamine N-oxide reductase
1e18.1.A	23.43	monomer	-	HHblits	X-ray	2.00Å	0.31	0.77	DMSO REDUCTASE.

Template	Seq Identity	Oligo-state	QSQE	Found by	Method	Resolution	Seq Similarity	Coverage	Description
1dms.1.A	22.30	monomer	-	HHblits	X-ray	1.88Å	0.30	0.77	DMSO REDUCTASE
5o31.1.8	16.41	monomer	-	HHblits	EM	4.13Å	0.28	0.70	NADH-ubiquinone oxidoreductase 75 kDa subunit, mitochondrial
5xtb.1.L	17.62	monomer	-	HHblits	EM	NA	0.28	0.70	NADH-ubiquinone oxidoreductase 75 kDa subunit, mitochondrial
5gpn.24.A	16.79	monomer	-	HHblits	EM	NA	0.28	0.70	NADH-ubiquinone oxidoreductase 75 kDa subunit
5lnk.1.C	16.29	monomer	-	HHblits	EM	3.90Å	0.28	0.71	Mitochondrial complex I, 75 kDa subunit
6qc5.1.C	16.29	monomer	-	HHblits	EM	NA	0.28	0.71	NADH:ubiquinone oxidoreductase core subunit S1
6qcf.1.C	16.29	monomer	-	HHblits	EM	NA	0.28	0.71	NADH:ubiquinone oxidoreductase core subunit S1
1q16.1.A	18.42	monomer	-	HHblits	X-ray	1.90Å	0.29	0.61	Respiratory nitrate reductase 1 alpha chain
3ir5.1.A	18.42	monomer	-	HHblits	X-ray	2.30Å	0.29	0.61	Respiratory nitrate reductase 1 alpha chain
3egw.1.A	18.42	monomer	-	HHblits	X-ray	1.90Å	0.29	0.61	Respiratory nitrate reductase 1 alpha chain
3ir7.1.A	17.98	monomer	-	HHblits	X-ray	2.50Å	0.29	0.61	Respiratory nitrate reductase 1 alpha chain
1y4z.1.A	18.42	monomer	-	HHblits	X-ray	2.00Å	0.29	0.61	Respiratory nitrate reductase 1 alpha chain
3ir6.1.A	18.42	monomer	-	HHblits	X-ray	2.80Å	0.29	0.61	Respiratory nitrate reductase 1 alpha chain
2ev3.1.B	11.76	monomer	-	HHblits	X-ray	2.68Å	0.28	0.18	Hypothetical protein Rv1264/MT1302
2ev3.1.A	11.76	monomer	-	HHblits	X-ray	2.68Å	0.28	0.18	Hypothetical protein Rv1264/MT1302
2ev4.1.A	11.76	monomer	-	HHblits	X-ray	2.28Å	0.28	0.18	Hypothetical protein Rv1264/MT1302
2ev4.1.B	11.76	monomer	-	HHblits	X-ray	2.28Å	0.28	0.18	Hypothetical protein Rv1264/MT1302
2ev2.1.A	11.76	monomer	-	HHblits	X-ray	2.35Å	0.28	0.18	Hypothetical protein Rv1264/MT1302
1m2j.1.A	17.65	monomer	-	HHblits	X-ray	1.70Å	0.30	0.18	Silent Information Regulator 2
2ev1.1.A	11.76	monomer	-	HHblits	X-ray	1.60Å	0.28	0.18	Hypothetical protein Rv1264/MT1302
4twi.1.A	25.00	monomer	-	HHblits	X-ray	1.79Å	0.32	0.13	NAD-dependent protein deacylase 1
3f0p.1.A	12.00	monomer	-	HHblits	X-ray	1.64Å	0.27	0.13	Alkylmercury lyase
1ici.1.A	26.09	monomer	-	HHblits	X-ray	2.10Å	0.32	0.12	TRANSCRIPTIONAL REGULATORY PROTEIN, SIR2 FAMILY
5u7c.2.A	12.00	monomer	-	HHblits	X-ray	1.75Å	0.27	0.13	Alkylmercury lyase
5u7a.1.A	12.00	monomer	-	HHblits	X-ray	1.53Å	0.27	0.13	Alkylmercury lyase
5u7b.2.A	12.00	monomer	-	HHblits	X-ray	2.00Å	0.27	0.13	Alkylmercury lyase
5u83.1.A	12.00	monomer	-	HHblits	X-ray	1.61Å	0.27	0.13	Alkylmercury lyase
5u88.1.A	12.00	monomer	-	HHblits	X-ray	1.80Å	0.27	0.13	Alkylmercury lyase
4qvg.3.A	8.00	homo-dimer	-	HHblits	X-ray	2.90Å	0.24	0.13	SibL
4qvg.2.B	8.00	homo-dimer	-	HHblits	X-ray	2.90Å	0.24	0.13	SibL
1m2g.1.A	25.00	monomer	-	HHblits	X-ray	1.70Å	0.33	0.13	Silent Information Regulator 2
1m2n.1.A	26.67	monomer	-	HHblits	X-ray	2.60Å	0.33	0.12	Silent Information Regulator 2
1m2n.1.B	26.67	monomer	-	HHblits	X-ray	2.60Å	0.33	0.12	Silent Information Regulator 2
1m2h.1.A	26.09	monomer	-	HHblits	X-ray	1.80Å	0.32	0.12	Silent Information Regulator 2
1m2k.1.A	26.09	monomer	-	HHblits	X-ray	1.47Å	0.33	0.12	Silent Information Regulator 2

Template	Seq Identity	Oligo-state	QSQE	Found by	Method	Resolution	Seq Similarity	Coverage	Description
4u1q.1.B	8.00	homo-dimer	-	HHblits	X-ray	2.09Å	0.24	0.13	SibL
3f0p.2.A	12.00	monomer	-	HHblits	X-ray	1.64Å	0.27	0.13	Alkylmercury lyase
1s6l.1.A	12.00	monomer	-	HHblits	NMR	NA	0.27	0.13	Alkylmercury lyase

The table above shows the top 50 filtered templates. A further 94 templates were found which were considered to be less suitable for modelling than the filtered list.
1ogy.1.A, 2e7z.1.A, 4k9k.1.A, 5c0t.1.A, 6gcs.1.A, 3ir7.1.A, 1kqf.1.A, 5tma.1.A, 1aa6.1.A, 4qvg.1.A, 1wcn.1.A, 6g2j.1.G, 5t5i.1.B, 6btm.1.B, 1e18.1.A, 6f0k.1.B, 2a3n.1.A, 6sdv.1.A, 3ir5.1.A, 3fn8.2.A, 4zp1.1.A, 5nqd.1.A, 4aay.1.A, 5o31.1.8, 1eu1.1.A, 2jzb.1.B, 3iwf.1.A, 5u7b.1.A, 3o5a.1.A, 3dt5.1.A, 2nya.1.A, 4dmr.1.A, 6qc5.1.C, 5tmx.1.A, 6amk.1.A, 5c0t.2.A, 1dmr.1.A, 6sdr.1.A, 2v45.1.A, 2fug.2.C, 5tmx.1.B, 2vpz.1.A, 6ib8.1.C, 6b9t.1.A, 1wcl.1.A, 1tmo.1.A, 6rfr.1.A, 6qcf.1.C, 1u9l.1.A, 2ivf.1.A, 3fn8.1.A, 3ir6.1.A, 2xbl.1.A, 6b9s.1.B, 5by2.1.A, 1g8k.1.A, 5e7o.1.A, 1h0h.1.A, 6tg9.1.A, 4x3q.1.B, 3trj.1.A, 6b9s.1.A, 1wlo.1.A, 5lnk.1.C, 6s6y.1.B, 1b0n.1.B, 2vpx.1.D, 1dms.1.A, 1fdo.1.A, 3f2g.1.A, 1u9l.2.A, 4ydd.1.A, 2r63.1.A, 2iv2.1.A, 3g68.1.A, 1e60.1.A, 1y4z.1.A, 4v4c.1.A, 1q16.1.A, 5gpn.24.A, 1g8j.1.A, 6rfq.1.A, 3knz.1.A, 4k9q.1.A, 2o3f.1.A, 6amk.1.B, 3fkj.1.A, 3egw.1.A, 2v3v.1.A, 3m9s.1.C, 6cz7.1.A, 3f2h.1.A, 5xtb.1.L, 4u6n.1.A
