# Supplementary material for: In silico analysis of phylogeny, structure, and function of arsenite oxidase from unculturable microbiome of arsenic contaminated soil
Source: J Genet Eng Biotechnol. 2021 Mar 29;19:47. doi: 10.1186/s43141-021-00146-x (PMC8006529; doi:10.1186/s43141-021-00146-x)
Supplement: Supplementary file 6 — Additional file 6. Graphical representation for amino acid distribution in Ramachandran plot of three predicted model. [file 43141_2021_146_MOESM6_ESM.pptx]

## Slide 1
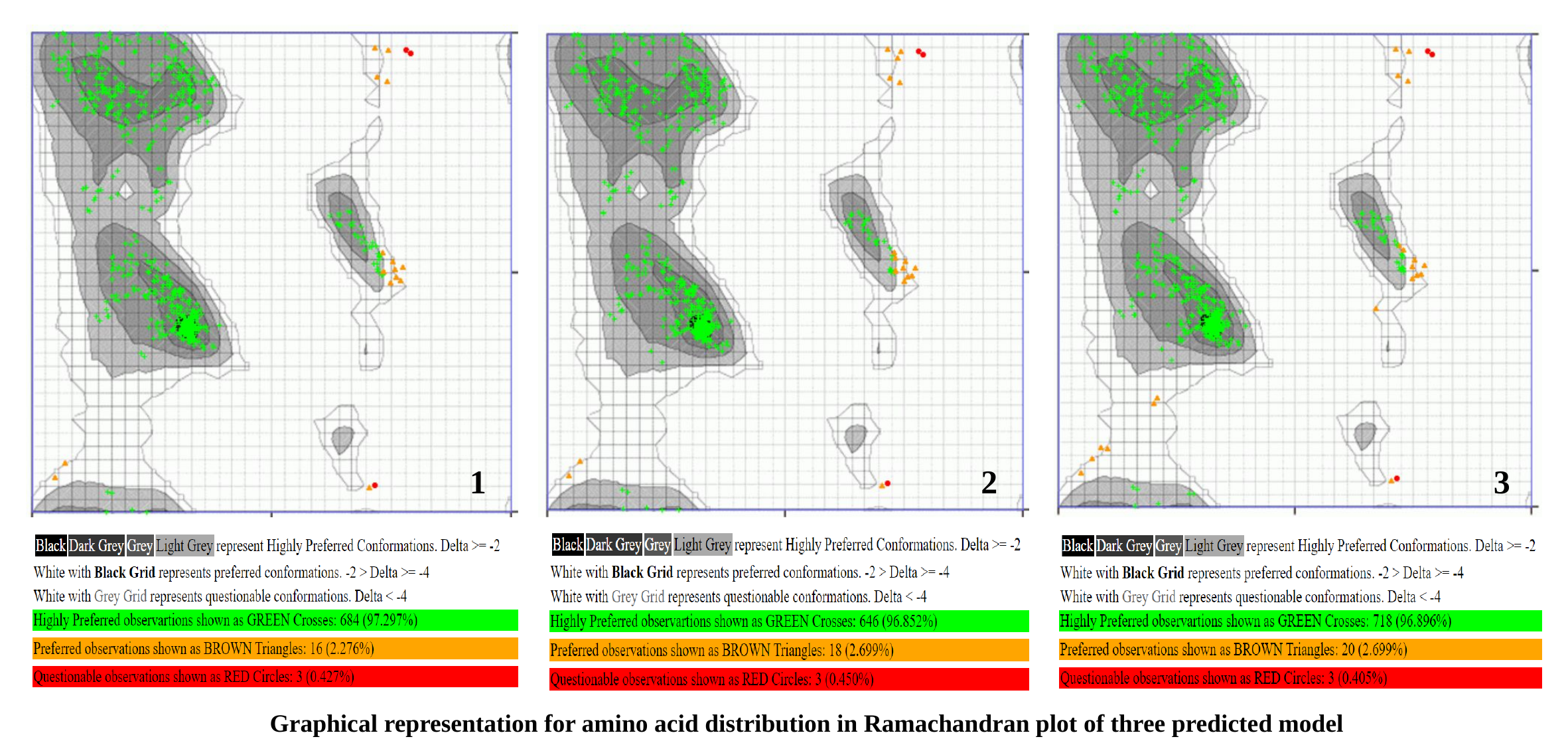

2
3
1
Graphical representation for amino acid distribution in Ramachandran plot of three predicted model
