# Supplementary material for: In silico analysis of phylogeny, structure, and function of arsenite oxidase from unculturable microbiome of arsenic contaminated soil
Source: J Genet Eng Biotechnol. 2021 Mar 29;19:47. doi: 10.1186/s43141-021-00146-x (PMC8006529; doi:10.1186/s43141-021-00146-x)
Supplement: Supplementary file 7 — Additional file 7. Visualization of alignments and QMEAN score (shown in blue bars) of model 1, 2, and 3 with template 5NQD. [file 43141_2021_146_MOESM7_ESM.pptx]

## Slide 1
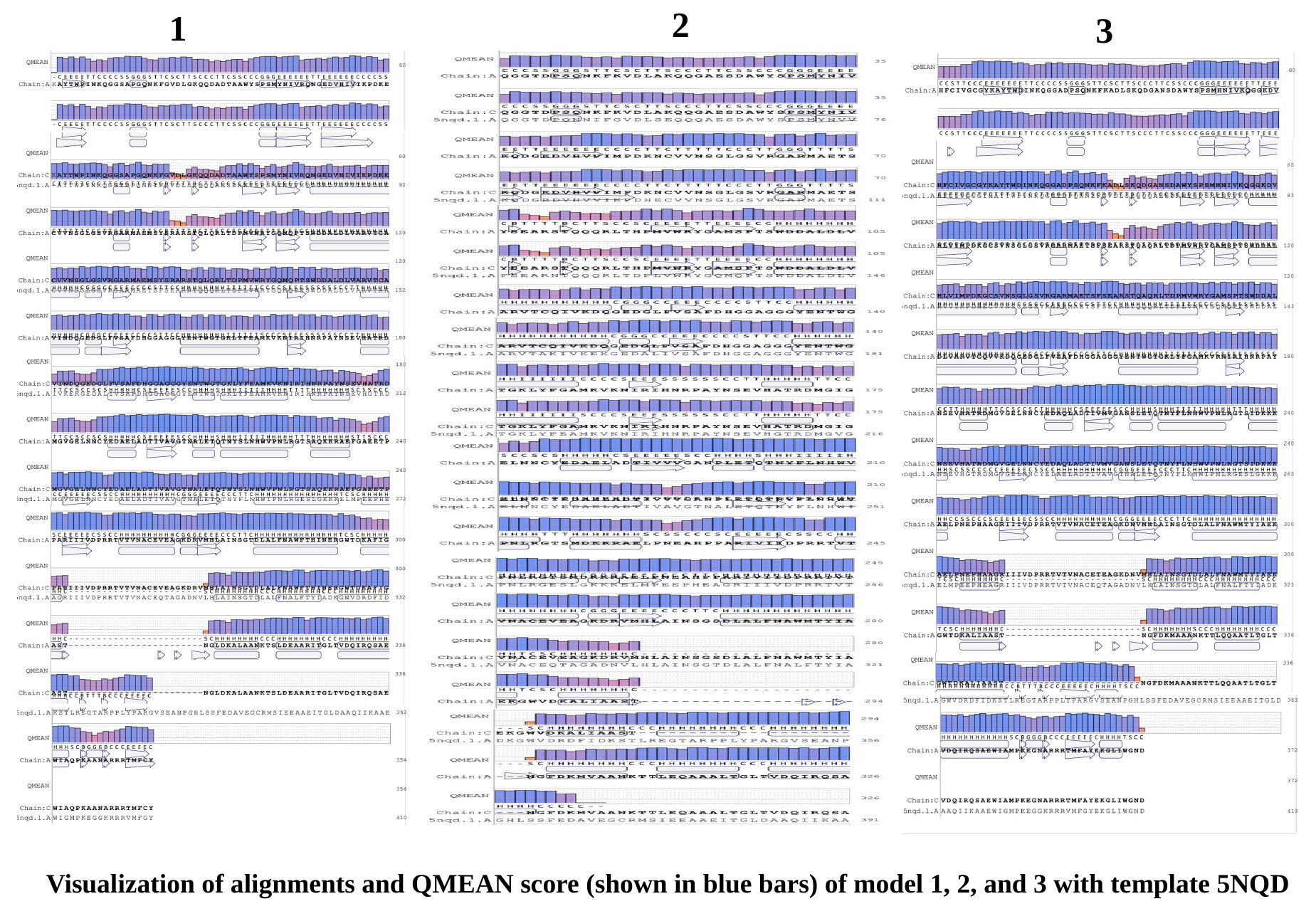

1
2
3
Visualization of alignments and QMEAN score (shown in blue bars) of model 1, 2, and 3 with template 5NQD
