# Supplementary material for: In silico analysis of phylogeny, structure, and function of arsenite oxidase from unculturable microbiome of arsenic contaminated soil
Source: J Genet Eng Biotechnol. 2021 Mar 29;19:47. doi: 10.1186/s43141-021-00146-x (PMC8006529; doi:10.1186/s43141-021-00146-x)
Supplement: Supplementary file 8 — Additional file 8. PROCHECK model evaluation report for query proteins. [file 43141_2021_146_MOESM8_ESM.pdf]

Ramachandran plots for all residue types

8866963

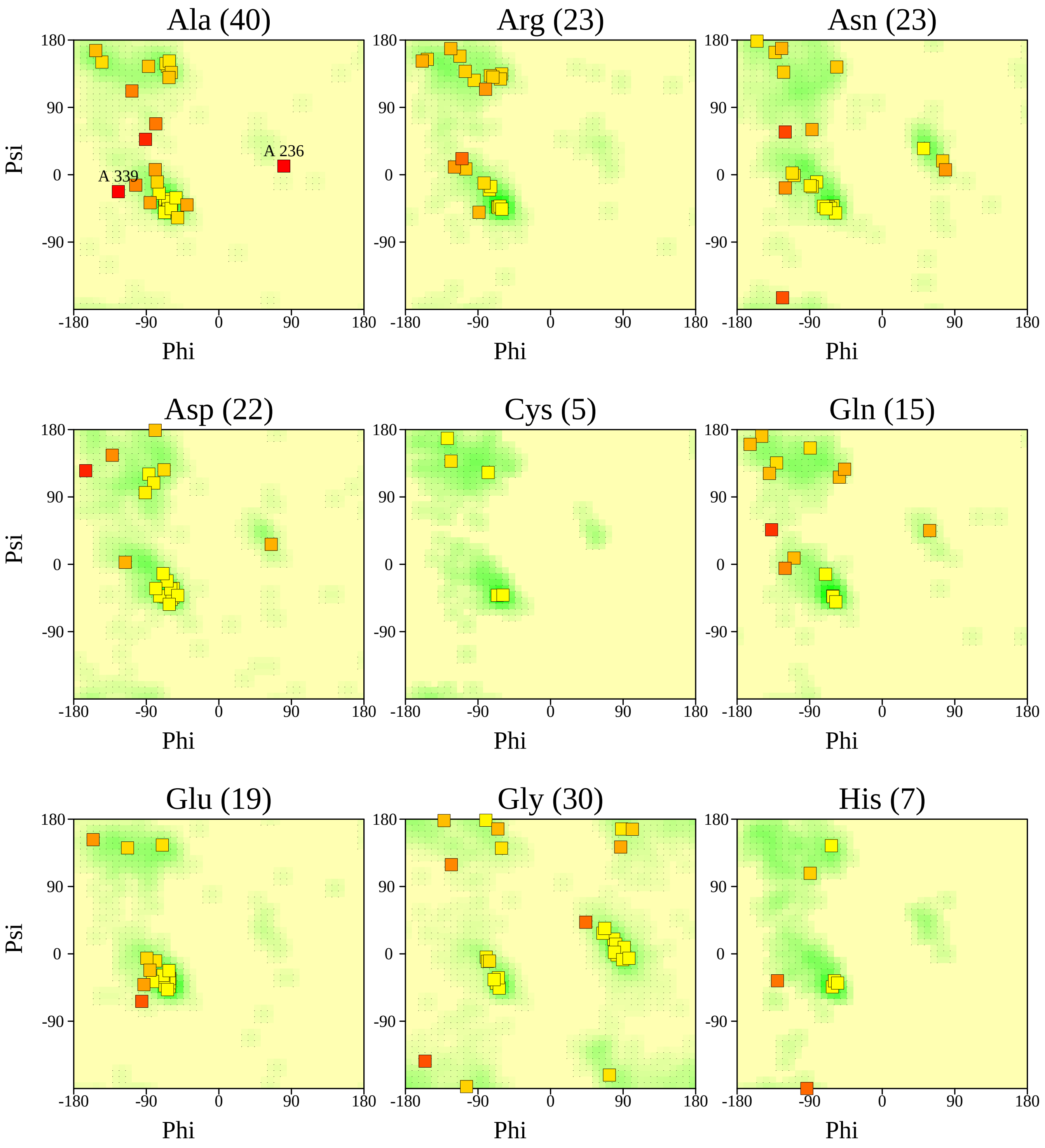

Numbers of residues are shown in brackets. Those in unfavourable conformations (score < -3.00) are labelled.
Shading shows favourable conformations as obtained from an analysis of 163 structures at resolution 2.0A or better.

Ramachandran plots for all residue types

8866963

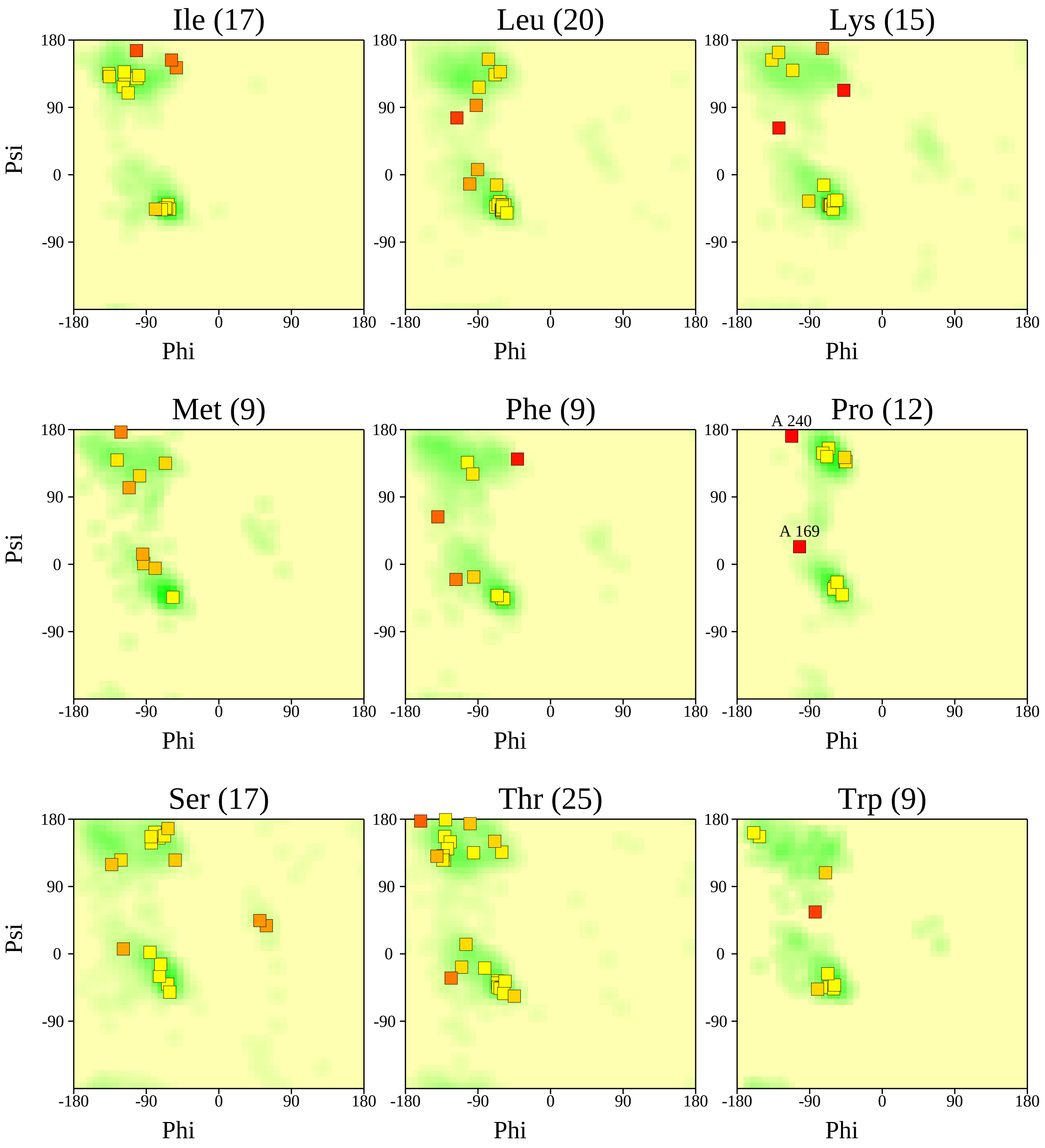

Numbers of residues are shown in brackets. Those in unfavourable conformations (score < -3.00) are labelled.
Shading shows favourable conformations as obtained from an analysis of 163 structures at resolution 2.0A or better.

Ramachandran plots for all residue types

8866963

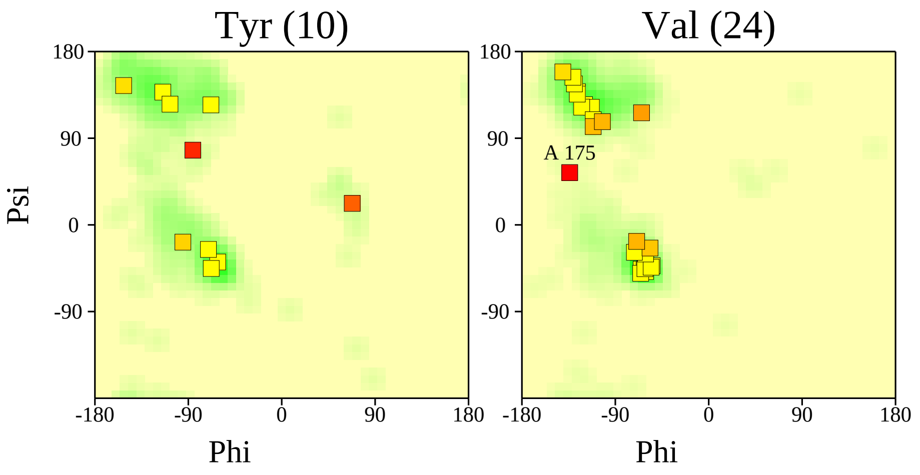

Numbers of residues are shown in brackets. Those in unfavourable conformations (score < -3.00) are labelled.
Shading shows favourable conformations as obtained from an analysis of 163 structures at resolution 2.0A or better.

PROCHECK

Chi1-Chi2 plots

8866963

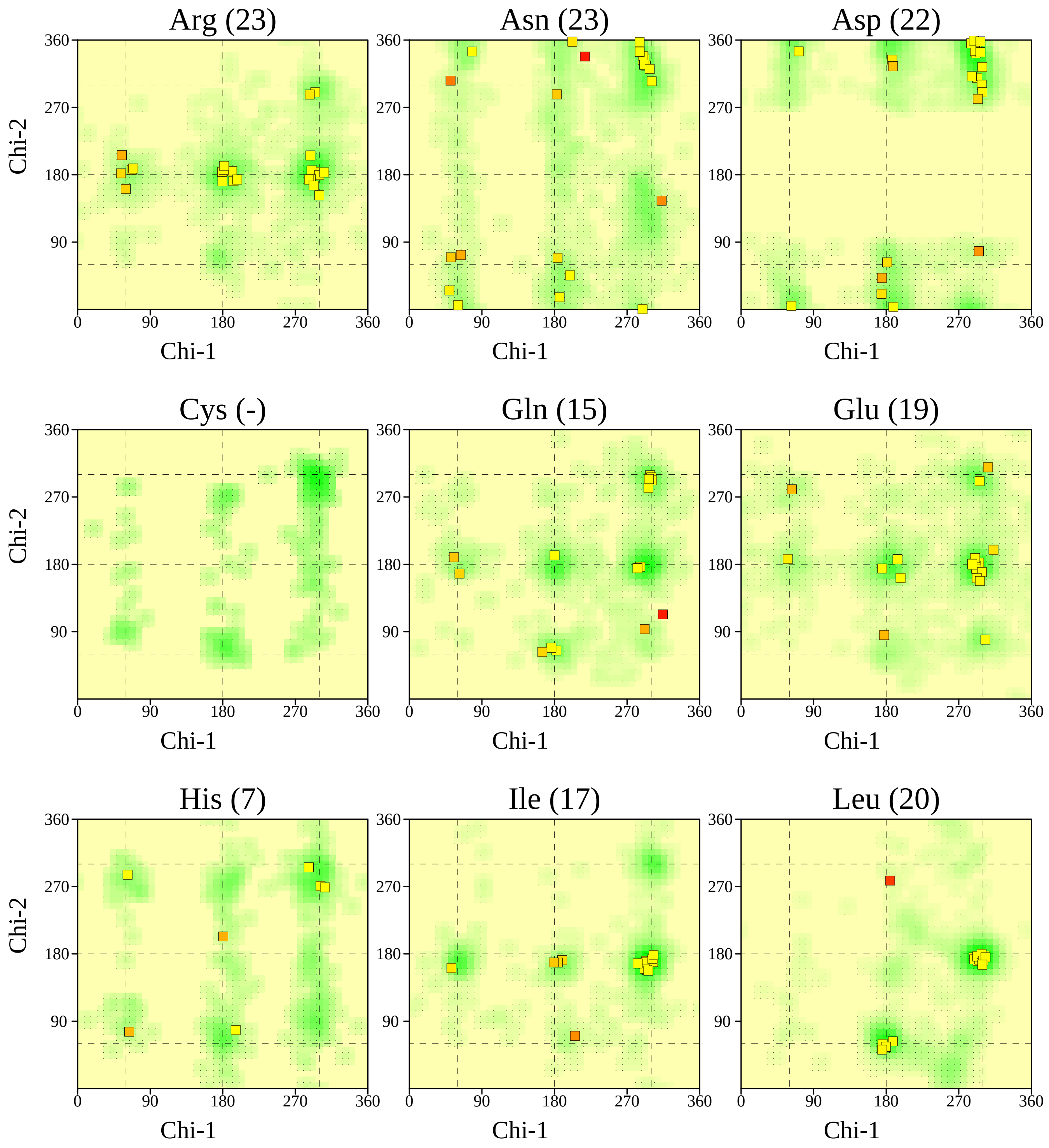

Numbers of residues are shown in brackets. Those in unfavourable conformations (score < -3.00) are labelled.
Shading shows favourable conformations as obtained from an analysis of 163 structures at resolution 2.0A or better.

Chi1-Chi2 plots

8866963

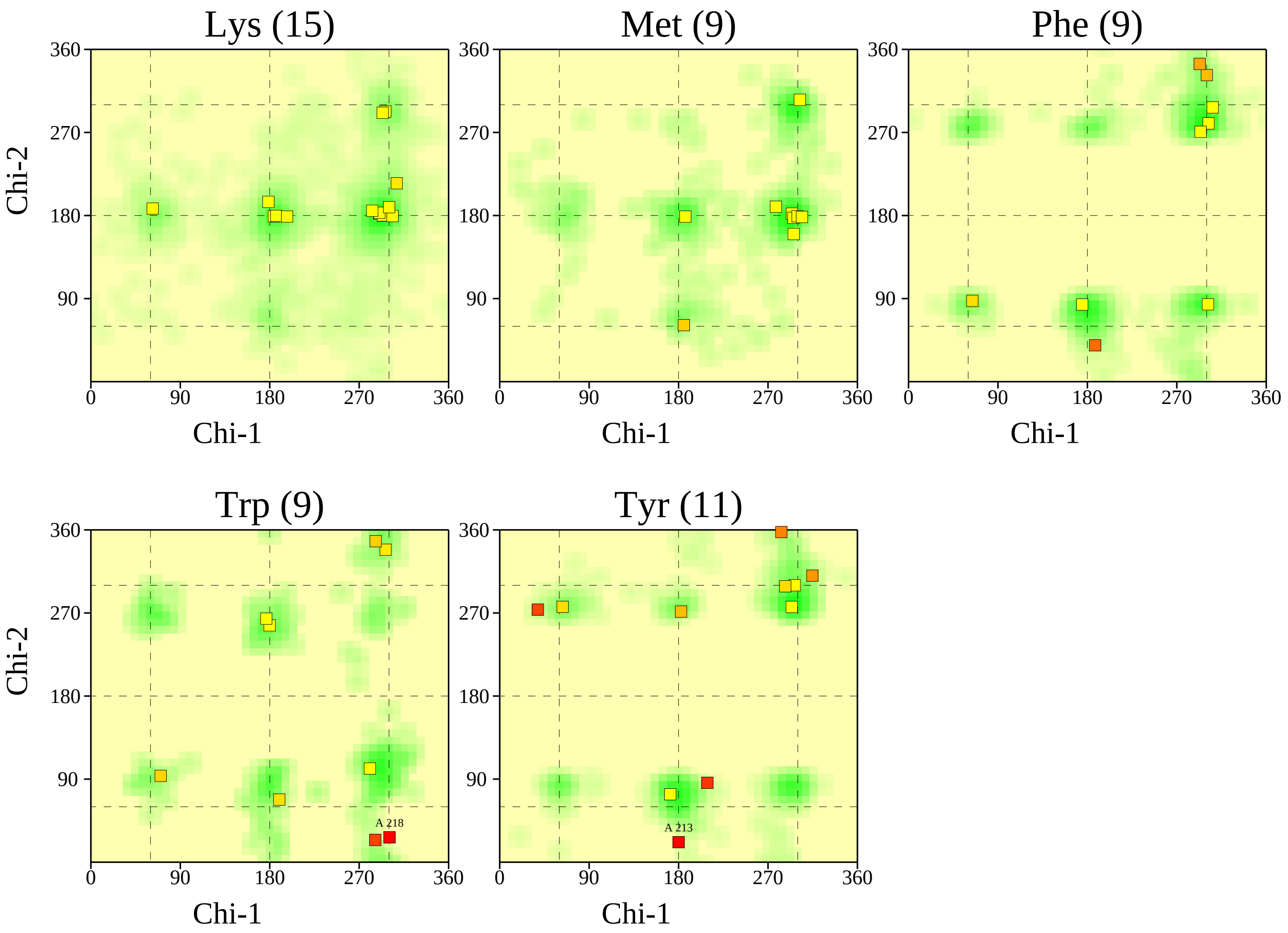

Numbers of residues are shown in brackets. Those in unfavourable conformations (score < -3.00) are labelled.
Shading shows favourable conformations as obtained from an analysis of 163 structures at resolution 2.0A or better.

PROCHECK

RMS distances from planarity

8866963

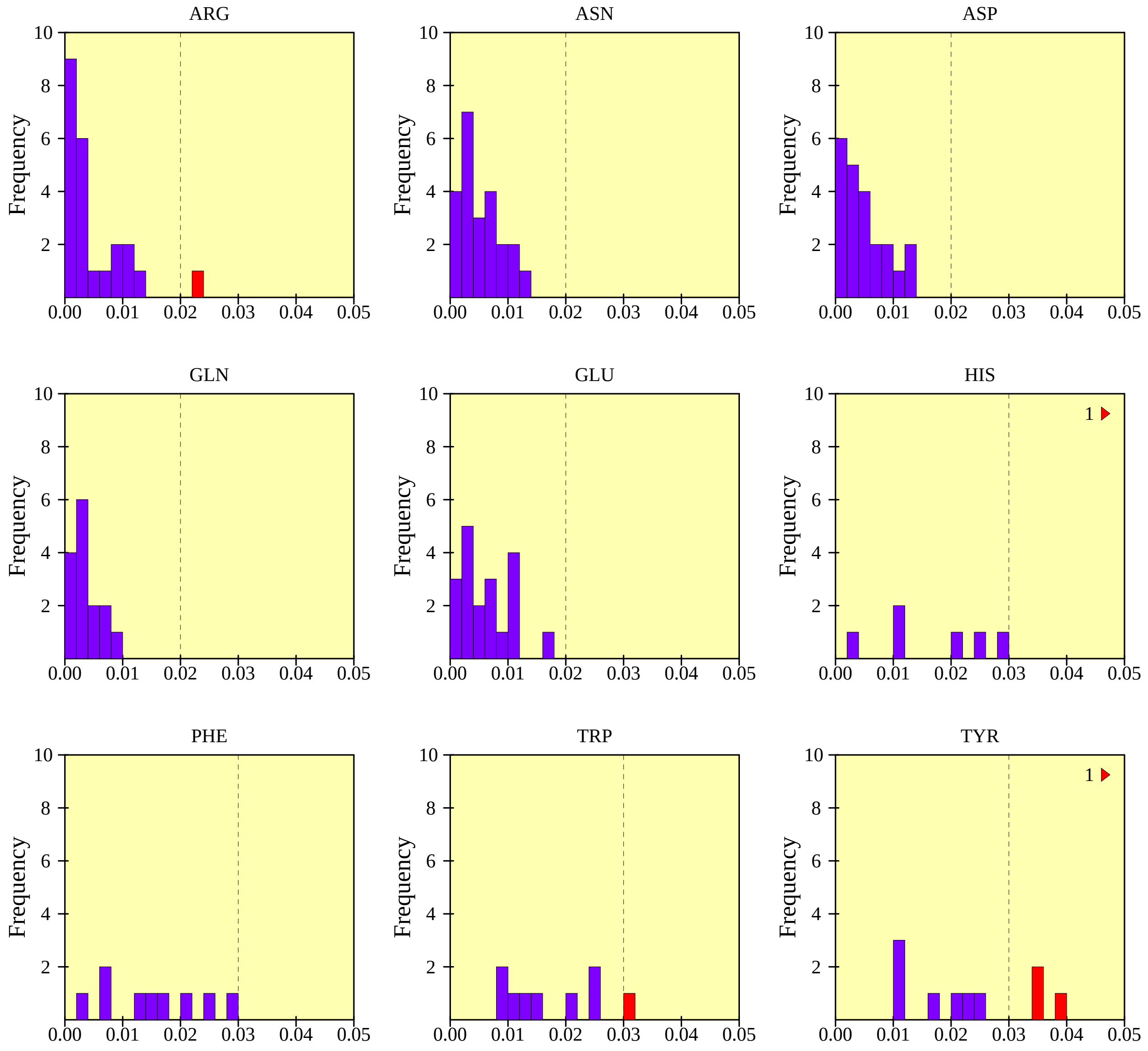

Histograms showing RMS distances of planar atoms from best-fit plane.
Black bars indicate large deviations from planarity: RMS dist > 0.03 for rings, and > 0.02 otherwise.

▸ signifies data points off the graph in the direction shown.

PROCHECK

Ramachandran Plot

3870781

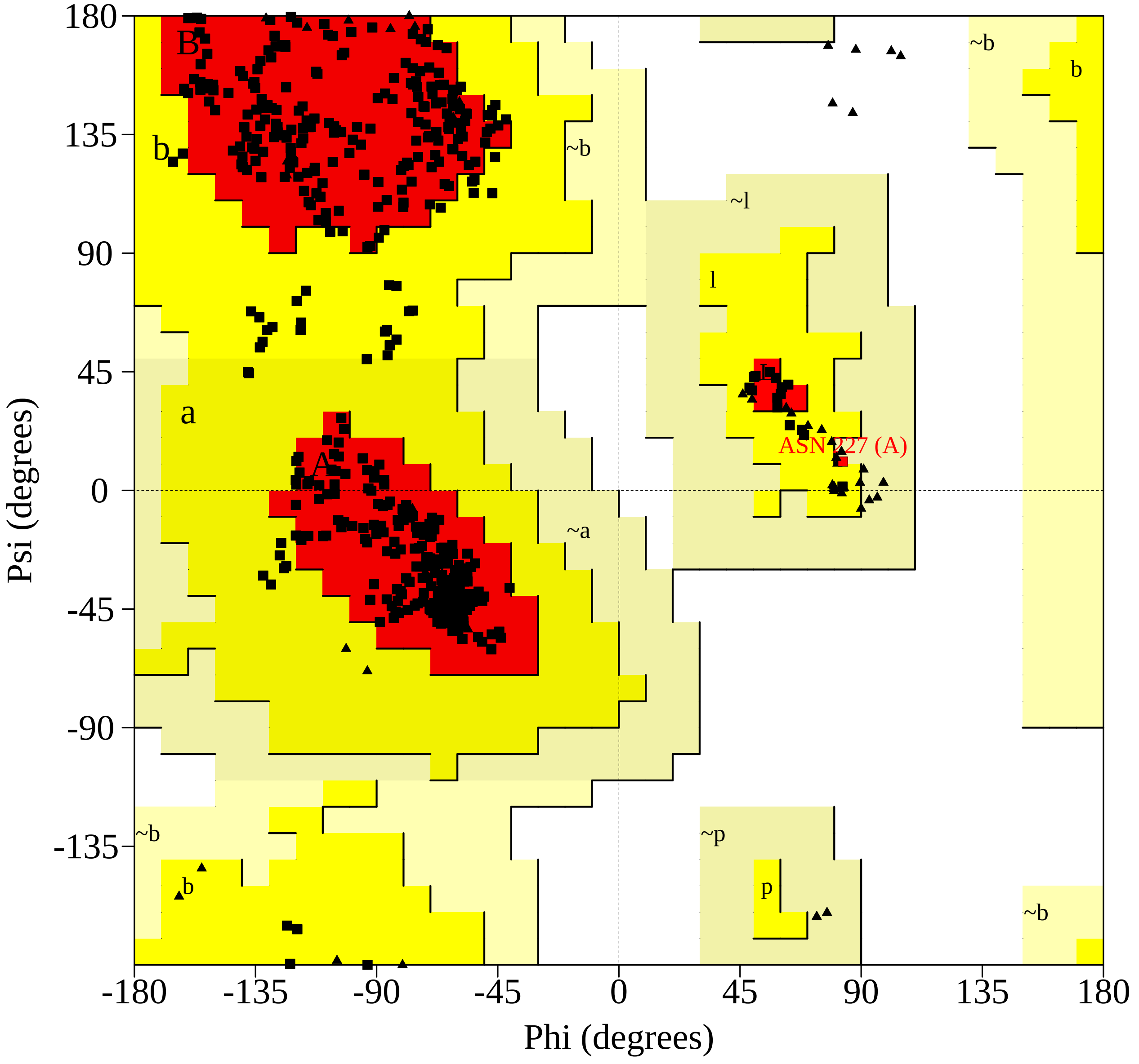

Plot statistics

Residues in most favoured regions [A,B,L]	530	90.4%
Residues in additional allowed regions [a,b,l,p]	55	9.4%
Residues in generously allowed regions [~a,~b,~l,~p]	1	0.2%
Residues in disallowed regions	0	0.0%
	----	------
Number of non-glycine and non-proline residues	586	100.0%
Number of end-residues (excl. Gly and Pro)	2	
Number of glycine residues (shown as triangles)	56	
Number of proline residues	26	

Total number of residues	670	

Based on an analysis of 118 structures of resolution of at least 2.0 Angstroms and R-factor no greater than 20%, a good quality model would be expected to have over 90% in the most favoured regions.

Ramachandran plots for all residue types

3870781

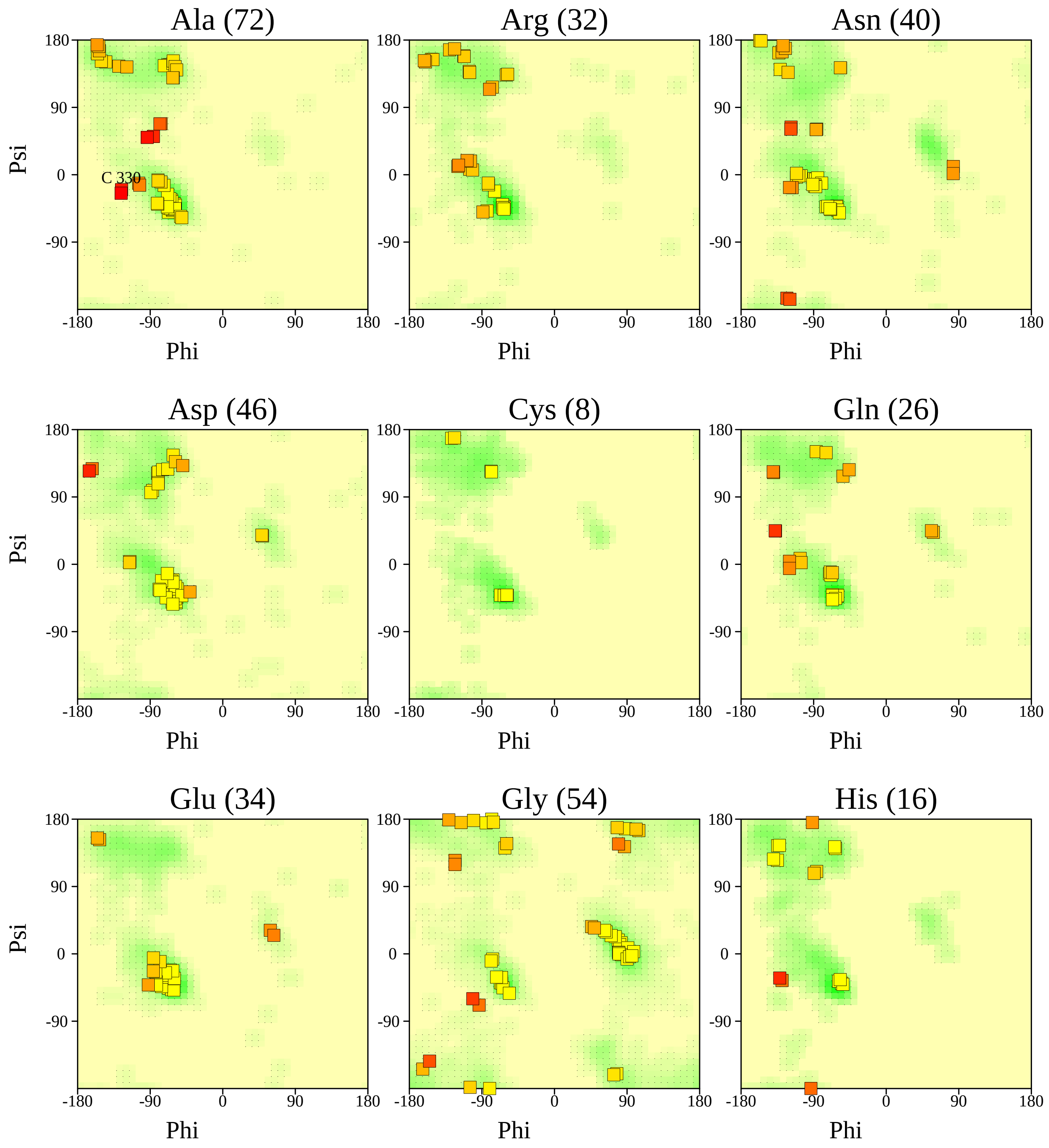

Numbers of residues are shown in brackets. Those in unfavourable conformations (score < -3.00) are labelled.
Shading shows favourable conformations as obtained from an analysis of 163 structures at resolution 2.0A or better.

Ramachandran plots for all residue types

3870781

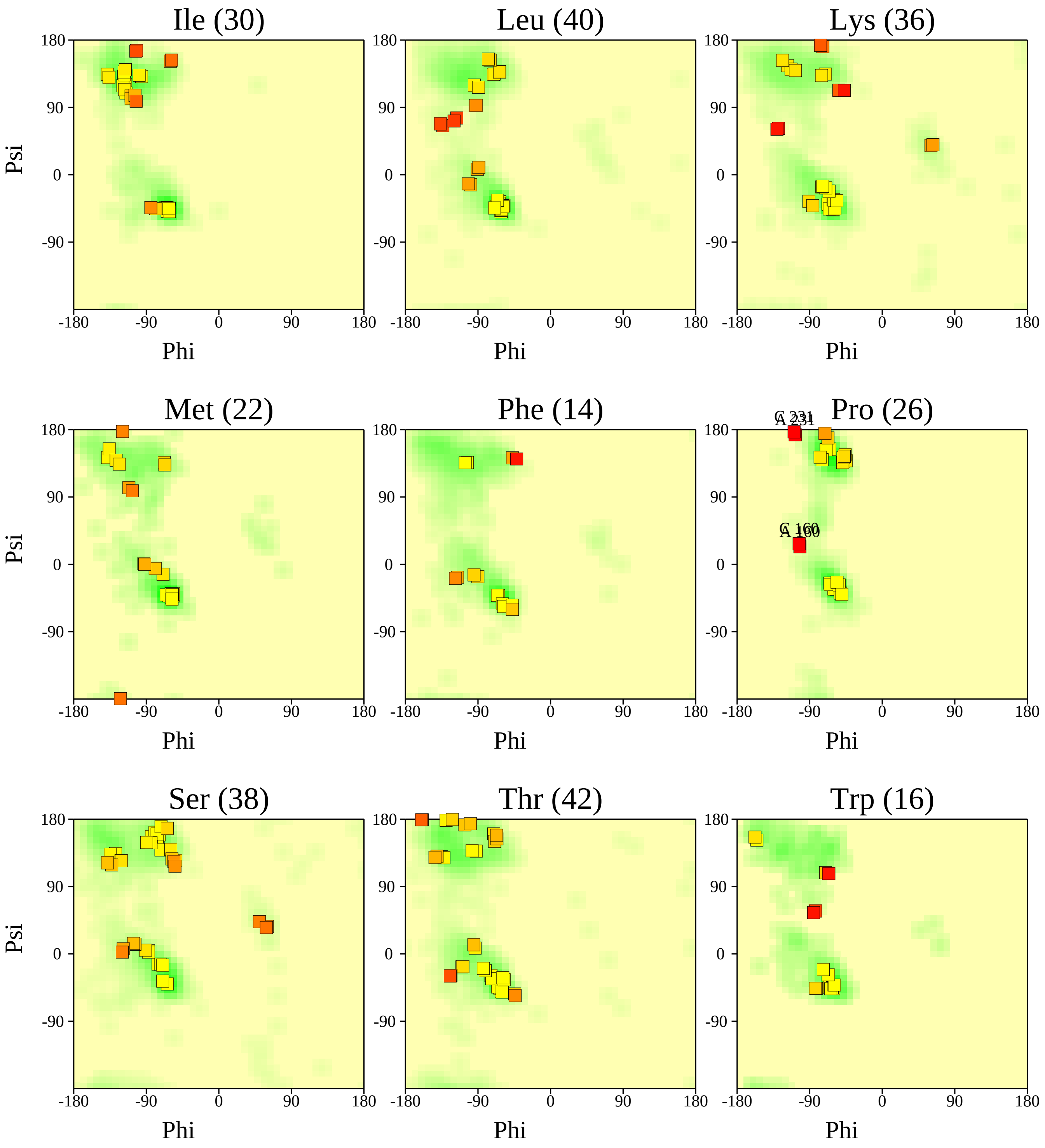

Numbers of residues are shown in brackets. Those in unfavourable conformations (score < -3.00) are labelled.
Shading shows favourable conformations as obtained from an analysis of 163 structures at resolution 2.0A or better.

Ramachandran plots for all residue types

3870781

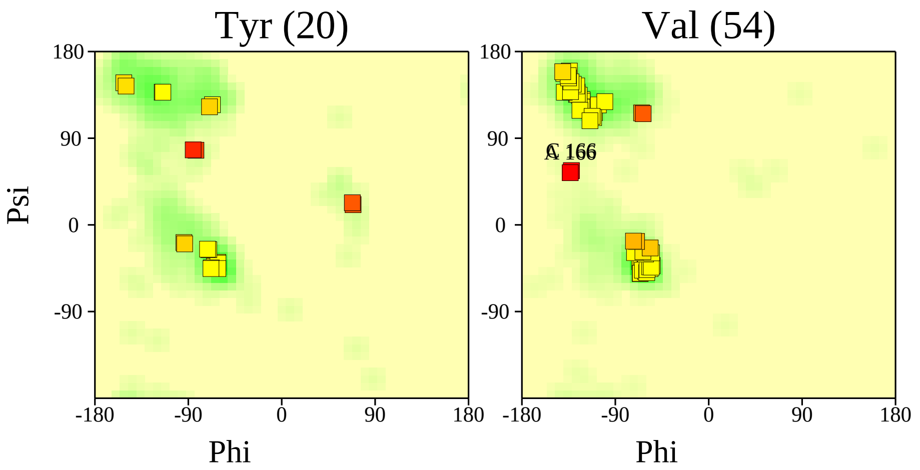

Numbers of residues are shown in brackets. Those in unfavourable conformations (score < -3.00) are labelled.
Shading shows favourable conformations as obtained from an analysis of 163 structures at resolution 2.0A or better.

PROCHECK

Chi1-Chi2 plots

3870781

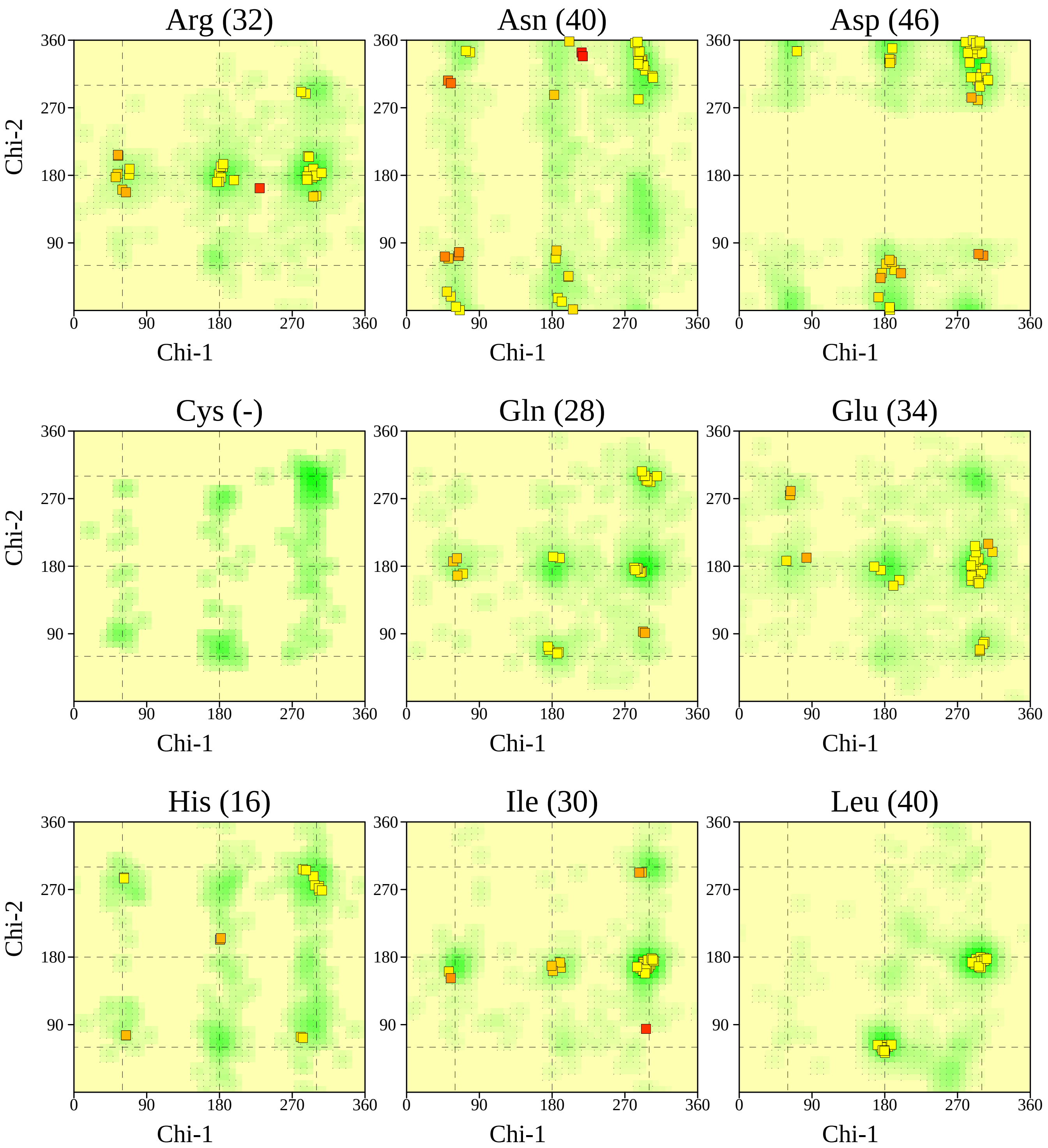

Numbers of residues are shown in brackets. Those in unfavourable conformations (score < -3.00) are labelled.
Shading shows favourable conformations as obtained from an analysis of 163 structures at resolution 2.0A or better.

Chi1-Chi2 plots

3870781

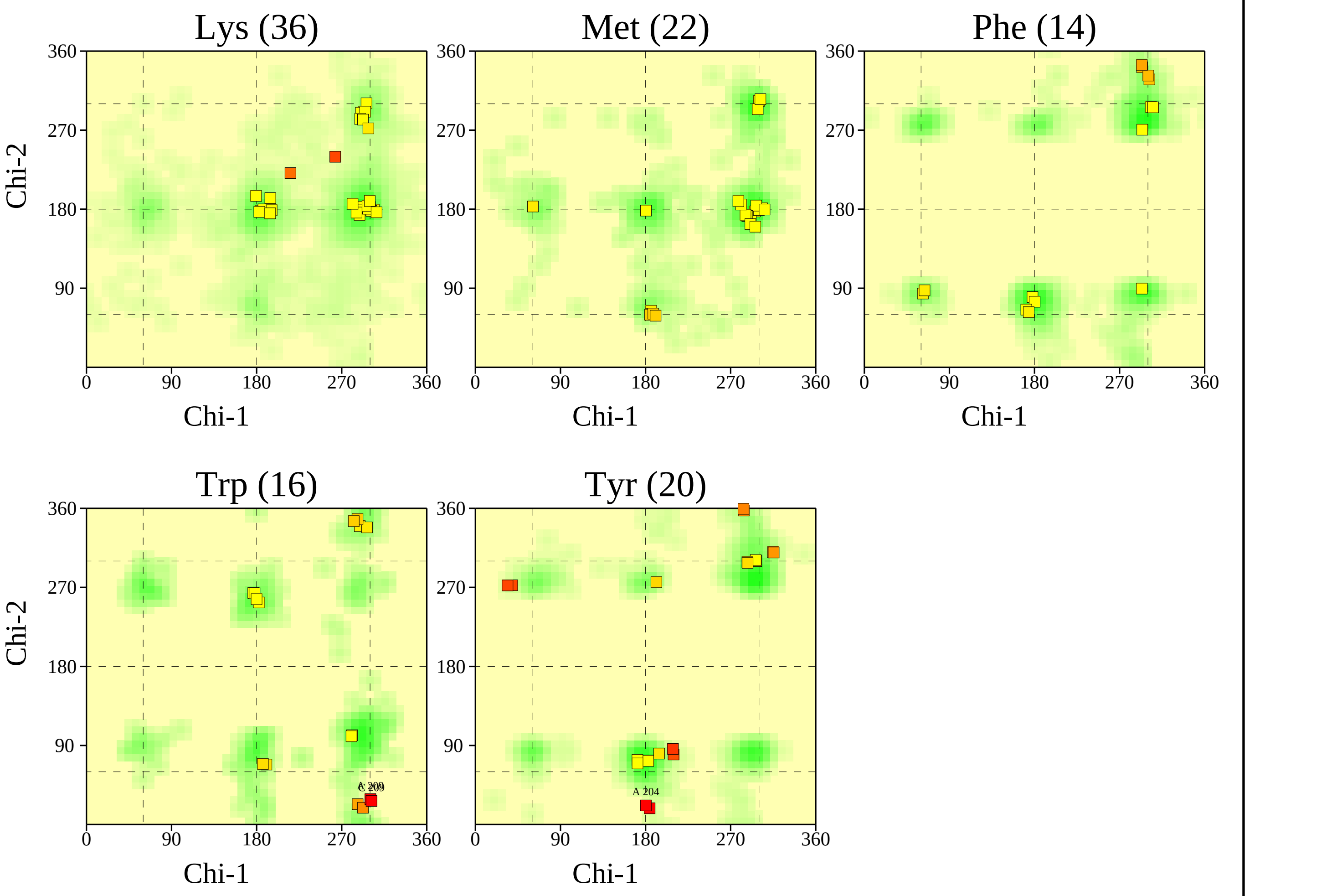

Numbers of residues are shown in brackets. Those in unfavourable conformations (score < -3.00) are labelled.
Shading shows favourable conformations as obtained from an analysis of 163 structures at resolution 2.0A or better.

PROCHECK

Ramachandran Plot

7403075

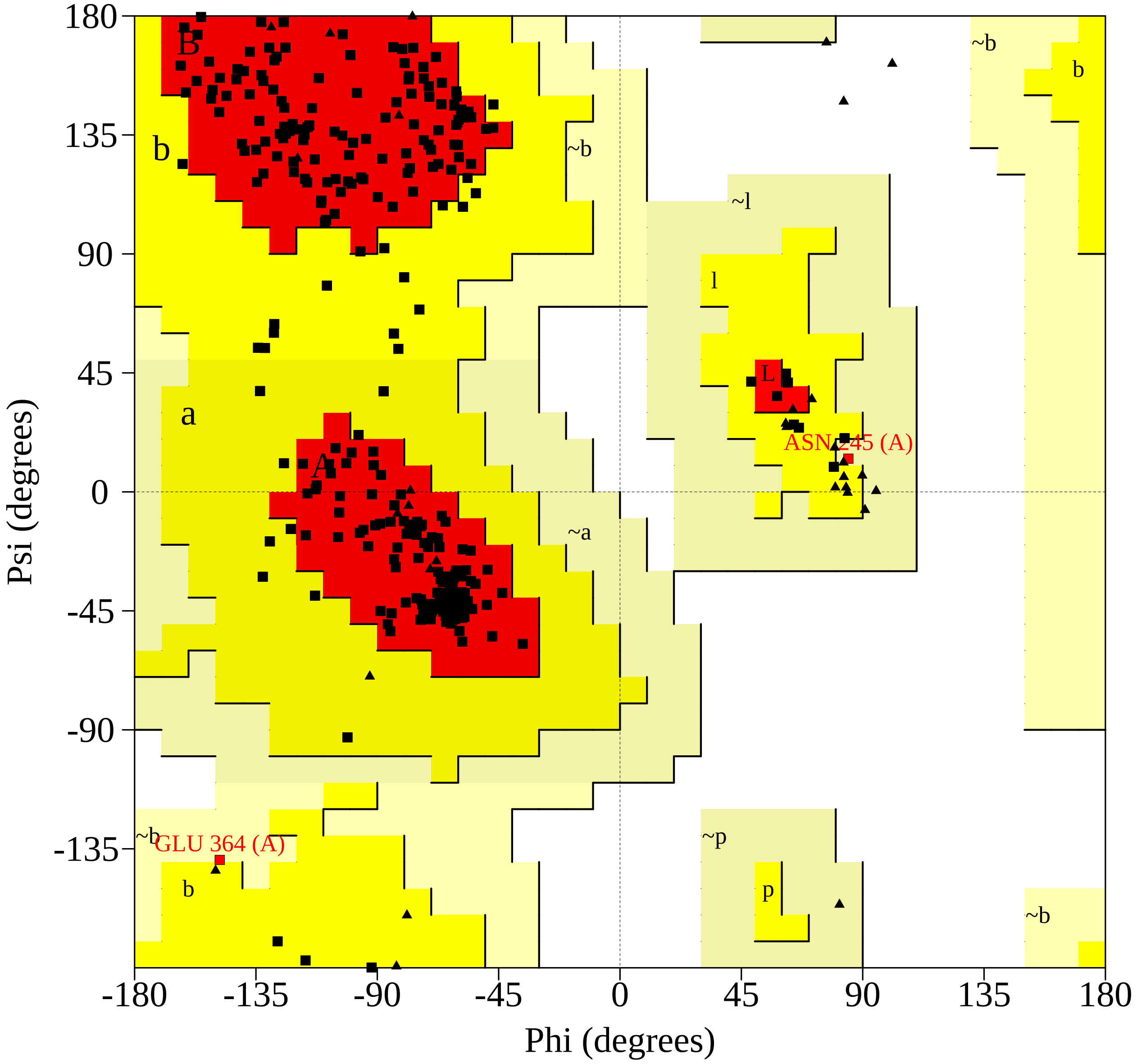

Plot statistics

Residues in most favoured regions [A,B,L]	289	89.2%
Residues in additional allowed regions [a,b,l,p]	33	10.2%
Residues in generously allowed regions [~a,~b,~l,~p]	2	0.6%
Residues in disallowed regions	0	0.0%
	----	------
Number of non-glycine and non-proline residues	324	100.0%
Number of end-residues (excl. Gly and Pro)	3	
Number of glycine residues (shown as triangles)	35	
Number of proline residues	11	

Total number of residues	373	

Based on an analysis of 118 structures of resolution of at least 2.0 Angstroms and R-factor no greater than 20%, a good quality model would be expected to have over 90% in the most favoured regions.

7403075_01.ps

PROCHECK

Ramachandran plots for all residue types

7403075

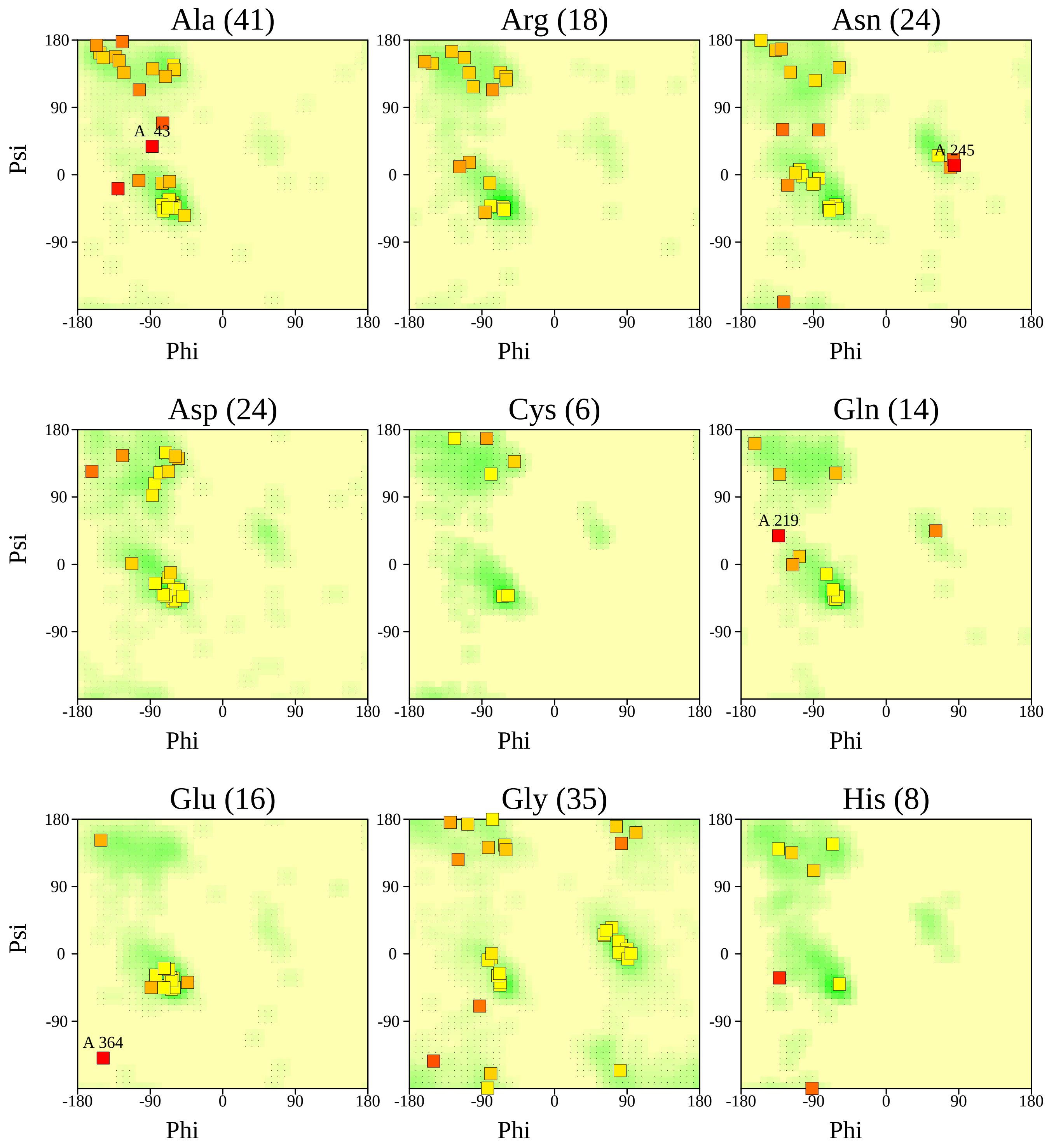

Numbers of residues are shown in brackets. Those in unfavourable conformations (score < -3.00) are labelled.
Shading shows favourable conformations as obtained from an analysis of 163 structures at resolution 2.0A or better.

Ramachandran plots for all residue types

7403075

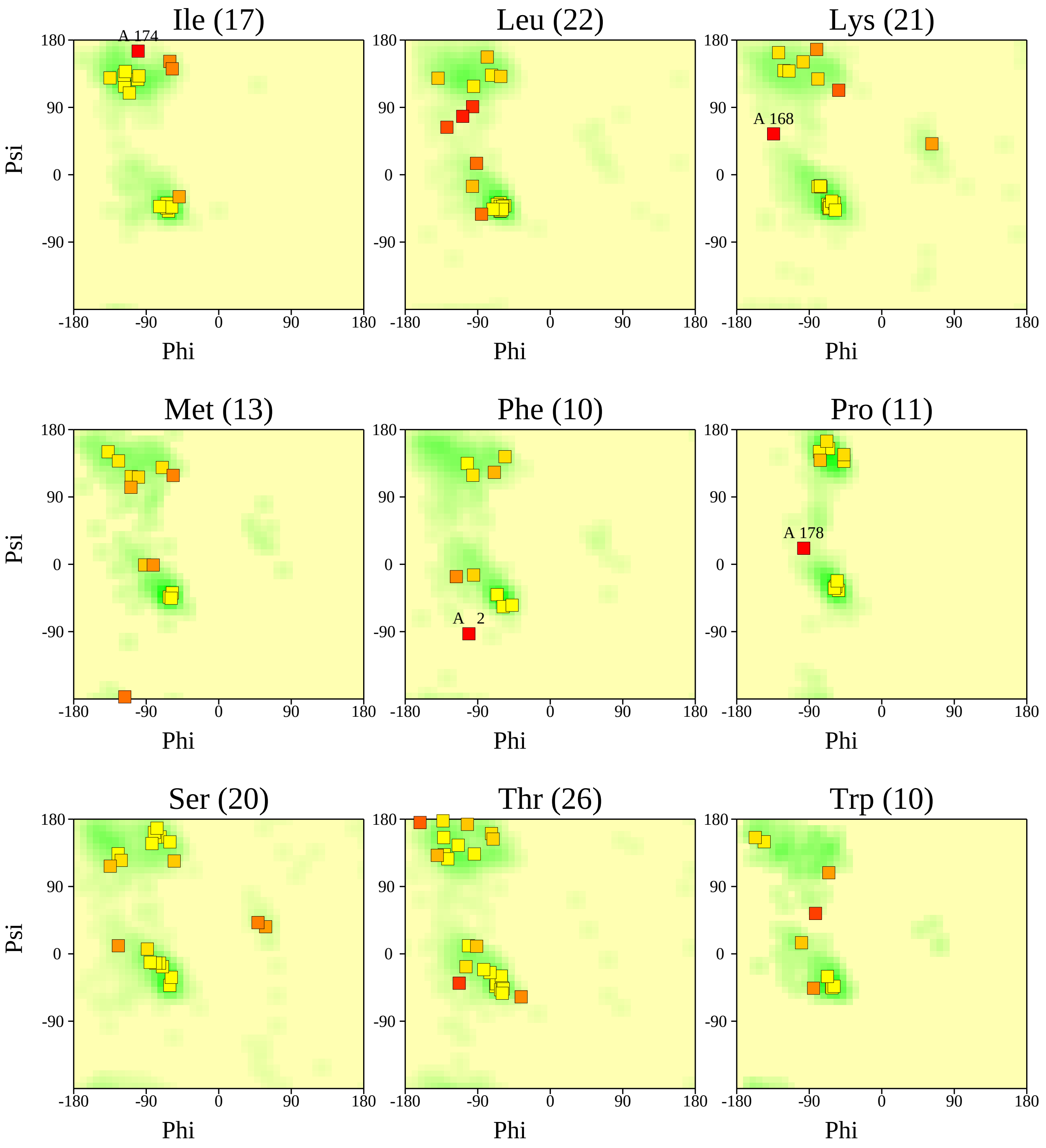

Numbers of residues are shown in brackets. Those in unfavourable conformations (score < -3.00) are labelled.
Shading shows favourable conformations as obtained from an analysis of 163 structures at resolution 2.0A or better.

Ramachandran plots for all residue types

7403075

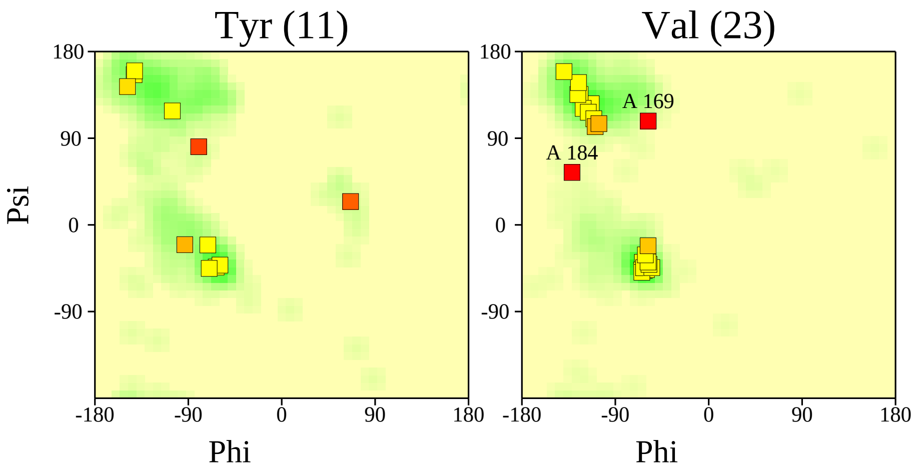

Numbers of residues are shown in brackets. Those in unfavourable conformations (score < -3.00) are labelled.
Shading shows favourable conformations as obtained from an analysis of 163 structures at resolution 2.0A or better.

PROCHECK

Chi1-Chi2 plots

7403075

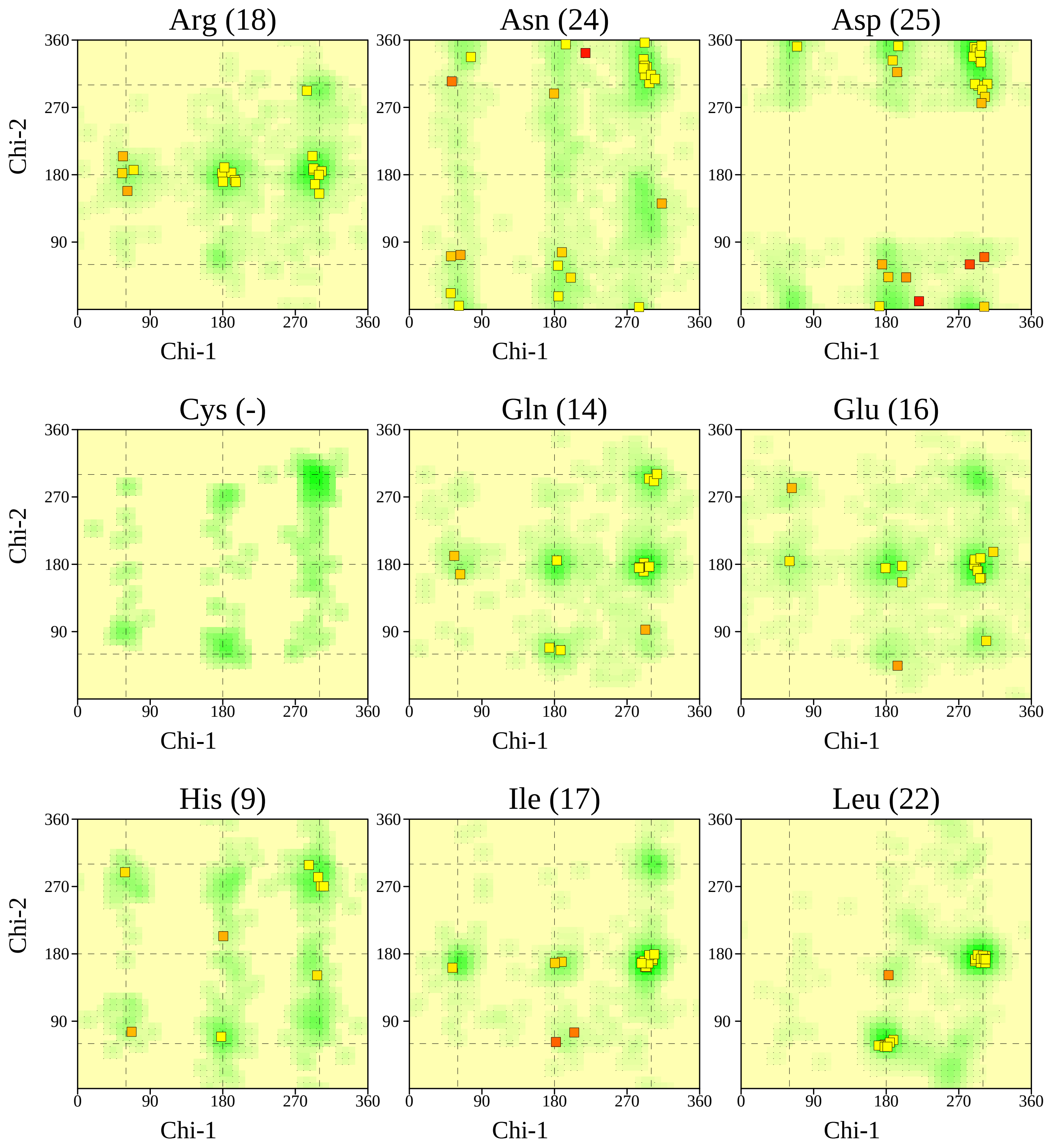

Numbers of residues are shown in brackets. Those in unfavourable conformations (score < -3.00) are labelled.
Shading shows favourable conformations as obtained from an analysis of 163 structures at resolution 2.0A or better.

Chi1-Chi2 plots

7403075

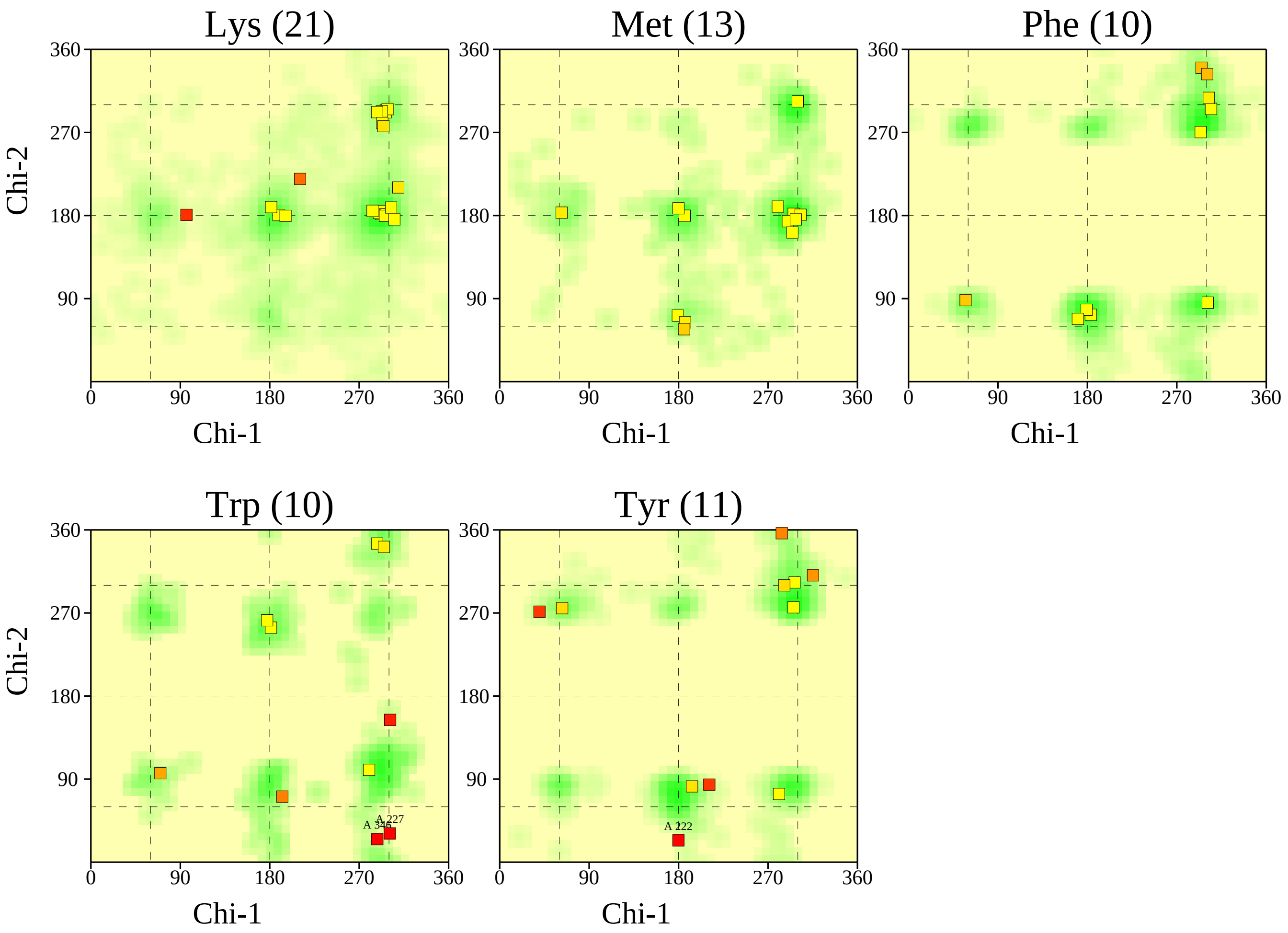

Numbers of residues are shown in brackets. Those in unfavourable conformations (score < -3.00) are labelled.
Shading shows favourable conformations as obtained from an analysis of 163 structures at resolution 2.0A or better.

PROCHECK

RMS distances from planarity

7403075

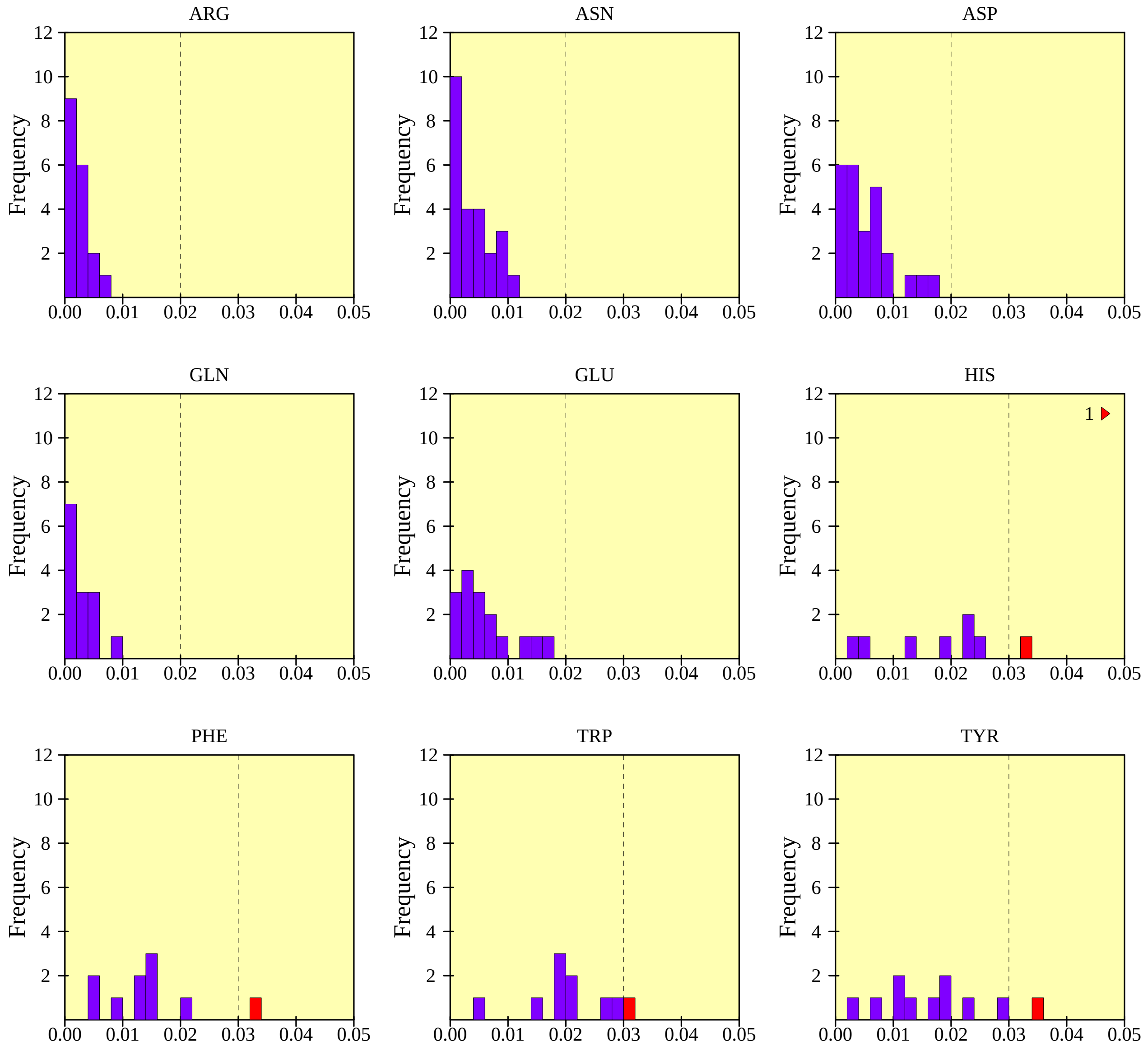

Histograms showing RMS distances of planar atoms from best-fit plane.
Black bars indicate large deviations from planarity: RMS dist > 0.03 for rings, and > 0.02 otherwise.

signifies data points off the graph in the direction shown.
