# Supplementary material for: In silico analysis of phylogeny, structure, and function of arsenite oxidase from unculturable microbiome of arsenic contaminated soil
Source: J Genet Eng Biotechnol. 2021 Mar 29;19:47. doi: 10.1186/s43141-021-00146-x (PMC8006529; doi:10.1186/s43141-021-00146-x)
Supplement: Supplementary file 9 — Additional file 9. Signal peptide prediction using SignalP 5.0 server in query proteins and their closest phylogenetic members. [file 43141_2021_146_MOESM9_ESM.pptx]

## Slide 1
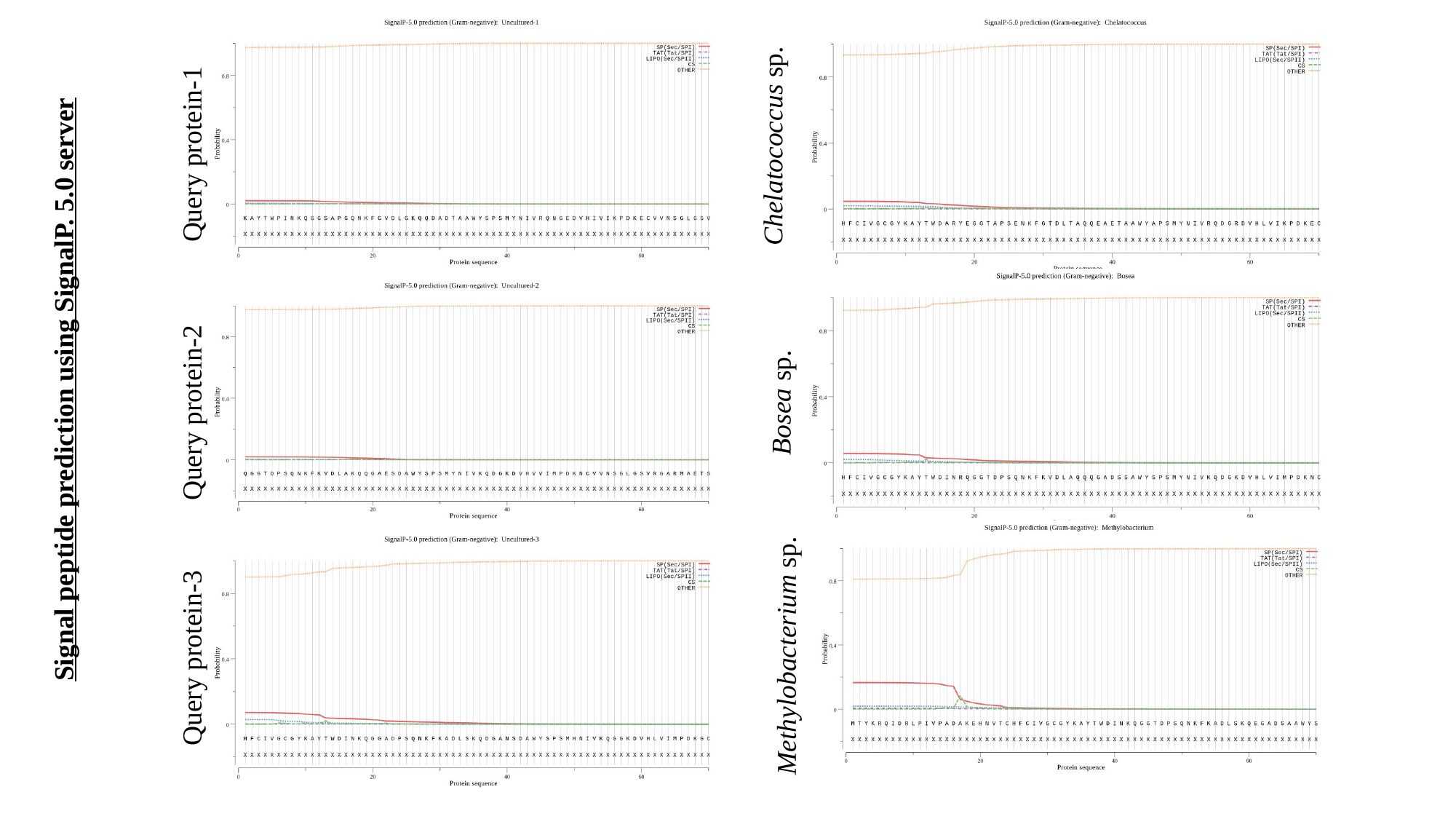

Query protein-1
Chelatococcus sp.
Bosea sp.
Query protein-2
Methylobacterium sp.
Query protein-3
Signal peptide prediction using SignalP. 5.0 server
